# Relative efficacy of interventions in the treatment of second-line non-small cell lung cancer: a systematic review and network meta-analysis

**DOI:** 10.1186/s12885-019-5569-5

**Published:** 2019-04-15

**Authors:** Adrian D. Vickers, Katherine B. Winfree, Gebra Cuyun Carter, Urpo Kiiskinen, Min-Hua Jen, Donald Stull, James A. Kaye, David P. Carbone

**Affiliations:** 1 RTI Health Solutions, The Pavilion, Towers Business Park, Wilmslow Road, Didsbury, Manchester, M20 2LS UK; 2 Eli Lilly and Company Limited, Indianapolis, IN USA; 3 Eli Lilly and Company Limited, Helsinki, Finland; 4 Eli Lilly and Company Limited, Windlesham, Surrey UK; 5 RTI Health Solutions, Research Triangle Park, NC USA; 6 RTI Health Solutions, Waltham, MA USA; 7 Ohio State University Medical Center, Columbus, OH USA

**Keywords:** Advanced/metastatic non-small cell lung cancer, Chemotherapy, Randomized clinical trials, Histology, PD-L1, TKI, EGFR, Systematic review, Network meta-analysis

## Abstract

**Background:**

Locally advanced or metastatic non-small cell lung cancer (NSCLC) that has progressed after first-line treatment has a poor prognosis. Recent randomized clinical trials (RCTs) have demonstrated survival benefits of alternative treatments to docetaxel. However, information is lacking on which patients benefit the most and what drug or regimen is optimal. We report a systematic review and network meta-analysis (NMA) of second-line treatments in all subgroup combinations determined by histology, programmed death ligand 1 (PD-L1) expression, and epidermal growth factor receptor (EGFR) mutation.

**Methods:**

MEDLINE, PubMed, EMBASE, Biosciences Information Service (using the Dialog Platform), Cochrane Library, and abstracts from scientific meetings were searched for RCTs published up to September 2015. Key outcomes were overall survival (OS) and progression-free survival (PFS). Bayesian hierarchical exchangeable NMAs were conducted to calculate mean survival times and relative differences for eight subgroups, using docetaxel as the reference comparator. For OS, the NMA was based on hazard ratios applied to a first-order fractional polynomial model fitted to the reference treatment. For PFS, a second-order fractional polynomial model was fitted to reconstructed patient-level data for the entire network of evidence.

**Results:**

The search identified 30 studies containing 17 different treatment regimens. Docetaxel plus ramucirumab was associated with a significant improvement in OS and PFS, relative to docetaxel, regardless of patient type. Docetaxel plus nintedanib showed similar efficacy to docetaxel plus ramucirumab in the nonsquamous populations. EGFR tyrosine kinase inhibitors (TKIs) erlotinib and gefitinib showed superior levels of efficacy in EGFR mutation-positive populations and the one PD-1 immunotherapy (nivolumab) studied showed superior efficacy in the populations exhibiting high PD-L1 expression.

**Conclusions:**

In the absence of head-to-head comparisons, we performed a mixed-treatment analysis to synthesize evidence of the efficacy of each treatment. Benefits are optimized by targeting specific treatments to individual patients guided by histology, PD-L1 expression, and EGFR mutation status.

**Systematic review registration:**

This review is registered in PROSPERO (registration number: CRD42014013780 available at www.crd.york.ac.uk/PROSPERO).

**Electronic supplementary material:**

The online version of this article (10.1186/s12885-019-5569-5) contains supplementary material, which is available to authorized users.

## Background

Lung cancer is the most commonly occurring life-threatening cancer in the world, with an estimated 1.8 million new cases diagnosed in 2012 (12.9% of all new cancers) [1]. Lung cancer is estimated to be the leading cause of cancer-related deaths in men in Europe (25% of all cancer-related deaths) and represents the second most common cause of cancer-related deaths in women in Europe (14% of all cancer-related deaths) [2]. In the United States, lung cancer is the leading cause of cancer-related deaths in both men and women (26% of all cancer-related deaths) [3]. Approximately 85% of newly diagnosed cases are classified as non-small cell lung cancer (NSCLC), while the remaining 10 to 15% are small cell lung cancer and fewer than 5% are lung carcinoid tumors [4]. NSCLC includes squamous cell carcinoma (approximately 30% of all lung cancers), adenocarcinoma (approximately 30–40% of all lung cancers), and large cell carcinoma (approximately 10–15% of all lung cancers) [5]. Because symptoms of the disease are nonspecific (e.g., cough, dyspnea, fatigue, weight loss) or absent, approximately 65% of patients globally present with advanced-stage disease (i.e., stage IIIB or stage IV) [5, 6].

The natural history of untreated advanced NSCLC is poor, with a median survival of 2 to 5 months from diagnosis [7]. In the first-line treatment setting, due to the introduction of new drugs and patient selection based on histological subtypes and driver mutations that influence the biology of these malignancies, the median survival for patients with advanced NSCLC receiving platinum-based chemotherapy in combination with agents targeting specific histologies and mutations has improved to 12 months or longer in randomized controlled trial populations [8]. However, despite the availability of multiple treatment options in the second-line setting, clinical outcomes remain poor. Response rates are, on average, less than 10%, and median survival is 7 to 9 months from the start of second-line therapy [9]. Only a fraction of patients diagnosed with this disease are alive and fit for trials of second-line therapy.

A number of novel agents targeting specific pathways associated with apoptosis, cell proliferation, angiogenesis, and other antineoplastic mechanisms have recently emerged. This research has led to the regulatory approval of regimens that offer improved clinical outcomes for patients with NSCLC, and these regimens are now reflected in current guidelines from the National Comprehensive Cancer Network in the United States [10] and from the European Society for Medical Oncology in Europe [11].

The inclusion of information on biomarkers such as epidermal growth factor receptor (EGFR) mutations and programmed death ligand 1 (PD-L1) expression in clinical trials and the evidence of how particular interventions behave according to tumor histology means that the evidence base in NSCLC is becoming increasingly complex. Previous meta-analytic studies have tended to either focus on the entire population such as Jansen [12], only one subgroup (adenocarcinoma; Popat et al. [13]), or have split the evidence base into subgroups such as the Institute for Clinical and Economic Review [14] (tyrosine kinase inhibitors [TKIs] and programmed death 1 [PD-1] immunotherapies) and Liang et al. [15] (TKIs in EGFR mutation populations). However, one network meta-analysis (NMA) on maintenance treatments was conducted using a hierarchical model with covariates for EGFR mutation, histology, and response to previous induction [16], and a study by Vale et al. [17] investigated the efficacy of TKIs with EGFR status. A range of different models also have been used to fit NMAs in NSCLC. Jansen [12] used fractional polynomials, the Institute for Clinical and Economic Review [14] used hazard ratio and parametric survival models (Weibull and Gompertz), and the remainder have relied on hazard ratios.

The aim of this study was to provide comprehensive evidence on the efficacy of available second-line treatment options for patients with advanced NSCLC of clinically meaningful subtypes defined by histology, PD-L1 expression, and EGFR mutation. For this purpose, we conducted an NMA with Bayesian hierarchical exchangeable structures which allow treatment effects to vary by covariates representing these subtypes.

## Methods

### Systematic literature review

A systematic literature review was undertaken to identify all relevant publications of phase 2/3 randomized controlled trials that were conducted in adult patients who had locally advanced or metastatic NSCLC and whose disease had progressed after first-line chemotherapy through 2 September 2015. There were no date, language, or geographical restrictions on the medical reference database searches. The search strategy involved searches of MEDLINE (using PubMed platform), MEDLINE In-Process (using PubMed platform), Embase (using Elsevier platform), Biosciences Information Service (using the Dialog Platform) and the Cochrane Library (using the Wiley platform). Additionally, the American Society of Clinical Oncology, the European Society of Medical Oncology, and the International Association for the Study of Lung Cancer were searched for relevant new evidence. The search included regimens containing the following interventions: docetaxel (any dose), erlotinib (150 mg), gefitinib (250 mg), gemcitabine (any dose), nintedanib (200 mg), nivolumab (3 mg/kg), pembrolizumab (any dose), pemetrexed (500 mg/m^2^), ramucirumab (10 mg/kg), vinorelbine (any dose), and best supportive care. Two further interventions also were included from this literature review due to growing interest in the Asian market. These were S-1 (40 mg/m^2^) and bevacizumab (15 mg/kg). After the systematic literature review had been completed, the trial results for pembrolizumab [18] were published online in December 2015 and made fully available in April 2016. That study was considered in addition to the 30 clinical trials identified for inclusion in the NMA.

Two independent reviewers performed the screening; where there was any uncertainty about inclusion, the study was checked by a third researcher. Data were extracted from full-text versions of studies, where available (i.e., abstracts or posters were not used unless an abstract or poster was the terminal source document). Where mature data from a single trial were reported later, the most recent data were used in the analysis.

### Outcome evaluation

The outcomes studied were OS and PFS, which were evaluated in terms of relative mean survival compared to the reference treatment (docetaxel 75 mg/m^2^). Although clinical trials typically report hazard ratios and median survival times, the fitting of parametric models to survival data lends itself more readily to the estimation of mean survival. Mean survival times are of particular importance to cost-effectiveness analyses, which are part of the health technology assessment process. Mean survival was estimated as the area under the probability of survival curve with a horizon of 60 years. Relative differences were assessed and mean estimates with 95% credible intervals presented. In addition, significance tests were performed on the difference between two distributions. The minimum of the proportions greater or less than 0 was used as a probability and converted to a two-tailed test by multiplying it by 2 [19]. These values can be interpreted in the same way as *P* values from a frequentist analysis, although they do not have the same statistical definition. Rank probability charts, cumulative probability charts and surface under the cumulative ranking (SUCRA) were also derived following the methods described by Salanti et al. [20]. However, caution is needed when interpreting SUCRA scores or associated rankings because they can be biased when interventions are not represented equally in a network and/or, interventions are not connected to the same reference treatment [21, 22]. The efficacy of each intervention was evaluated for each combination of NSCLC factors, namely EGFR mutation positive (versus EGFR mutation negative), squamous versus nonsquamous, and PD-L1 expression < 5% versus PD-L1 expression ≥5% (PD-L1 expression < 1% vs. PD-L1 expression ≥1% was also studied, but only the full results for the 5% cut-off are presented), which together defined 16 distinct subgroups. Studies investigating TKI regimens included only TKI-naïve patients, and studies investigating nivolumab included only PD-L1 immunotherapy-–naïve patients.

### Statistical analysis

Kaplan-Meier charts were digitized using the DigitizeIt software package [23], and the method described by Guyot et al. [24] was used to reconstruct patient-level data. All of the data were plotted to check for evidence of nonproportional hazards and tests of significance performed using the method described by Grambsch and Therneau [25] based on a Kaplan-Meier transformation. The method presented by Jansen [12] was used to fit first- and second-order fractional polynomial models. In addition, hazard ratios and confidence intervals also were extracted and summarized as log (hazard ratios) and standard errors. The log (hazard ratios) were used to estimate Higgin’s I^2^ [26], which provides a measure of the percentage of variance explained by heterogeneity and/or inconsistency. Pairwise meta-analyses of duplicate comparisons and two types of node-splitting (traditional node-splitting [27], which detects only inconsistency for a closed loop in the network of evidence, and an experimental node-splitting [28], which assumes inconsistency is another measure of heterogeneity) also were conducted. The log (hazard ratios) were used to perform NMAs; this followed the method described by Woods et al. [29]. If a study did not provide complete information on hazard ratio estimates, the hazard ratios were estimated using the reconstructed patient-level data where possible; if not available, the hazard ratios were derived from median survival times and sample size following the method described by Hackshaw [30]. All the NMAs were extended using hierarchical exchangeable structures by adapting the code presented by Owen et al. [31]. Hierarchical exchangeable structures allow treatment effects to vary by covariates independently of the other treatments in the network of evidence. The treatment effect remains constant for any treatment not specified within a hierarchical exchangeable structure. The efficacy of EGFR TKIs was allowed to vary by EGFR mutation status (evidence supported by Wang et al. [32]; Lim et al. [33]; Sun et al. [34]; Urata et al. [35]; Vale et al. [17]). Further, pemetrexed was allowed to vary by histology (supported by evidence from Kubota et al. [36]; Scagliotti et al. [37]), nintedanib was allowed to vary by histology (supported by evidence from Reck et al. [38]), and nivolumab was allowed to vary by PD-L1 expression and histology (supported by evidence from Borghaei et al. [39]; Brahmer et al. [40]). Predictions were made for all combinations of these subgroups. In addition, where possible, different doses also were included as a hierarchical structure with an overall treatment class effect. Constraints were imposed to ensure that the efficacy increased with dose intensity (docetaxel 75 mg/m^2^ every 3 weeks → docetaxel 60 mg/m^2^ every 3 weeks → weekly lower-dose docetaxel) and where there was external supporting evidence for the treatment covariate interactions (EGFR TKIs with EGFR mutation status and pemetrexed with histology). Where there was sufficient evidence for subpopulation results within a trial, these were used instead of the overall study-level results. Subgroup data were used in the NMA only where we had a reason to suspect there should be a difference between subgroups, or there was supporting evidence in the literature, or a treatment had an indication for a subgroup. Studies that did not present the relevant proportion of patients with a given tumor subtype were not included in the NMA, i.e., pemetrexed and proportion of patients with nonsquamous tumors not reported, TKIs and proportion of patients with tumors that were EGFR mutation positive not reported, and PD-L1 immunotherapies with proportion of patients with PD-L1 expression not reported. The following study-level covariates were included one at a time to see if they improved model fit: time since publication, proportion of patients with nonsquamous tumors, mean age, proportion of patients with Eastern Cooperative Oncology Group (ECOG) ≥ 1, proportion of patients with metastatic disease, and proportion of Asian patients.

Noninformative priors were used for treatment efficacy and for between-study variances. For the fractional polynomial model, four random-effects models were conducted: first-order fractional polynomial NMA with an additional heterogeneity parameter for scale, first-order fractional polynomial NMA with additional heterogeneity parameters for scale and shape, second-order fractional polynomial NMA with an additional heterogeneity parameter for scale, and second-order fractional polynomial NMA with additional heterogeneity parameters for scale and the two shape parameters. A range of power functions were fitted for first- and second-order fractional polynomial models, which followed that of Jansen [12].

Posterior sampling was performed using Markov Chain Monte Carlo, with three chains of 70,000 iterations per chain, each with a burn-in of 30,000 iterations for the fractional polynomial models and a total of 120,000 iterations for the hazard ratio models. Convergence was assessed through iteration plots, the shape of the posterior distributions, and Gelman-Rubin diagnostics [41]. Bayesian NMAs were performed using JAGS software [42] within R 3.1.3 [43]. Heterogeneity tests were performed using the netmeta package in R [44] and node-splitting was performed using the geMTC package in R [45]. Further details and the JAGS code are provided in the Additional file 1.

Further analyses were required to estimate mean survival times. For the hazard ratio approach, all of the reconstructed patient-level data for the reference data were collated. Bayesian first-order and second-order fractional polynomial models were fitted to these data with heterogeneity parameters for all scale and shape parameters. The same number of iterations and chains was used as stated for the hazard ratio NMA. A grid search for the best-fitting model with up to two covariates was performed.

The efficacy of study treatments in each tumor subgroup was estimated by linear extrapolation of the parameters available for each proportion of the relevant tumor type. If not all of the required information was available, the efficacy of a combination regimen was estimated using the separate information from monotherapy regimens.

## Results

### Search results

**Fig. 1:**
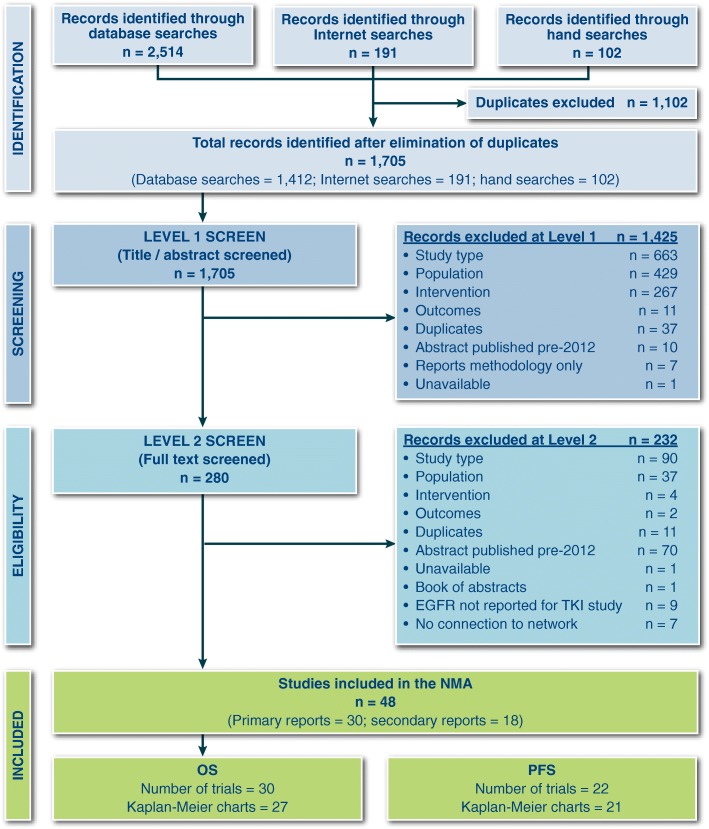
PRISMA Chart. PRISMA = Preferred Reporting Items for Systematic Reviews and Meta-Analyses. Note: Some studies were reported in multiple publications; in such cases, the main study report was classed as the “primary” publication, and any other articles reporting on the same trial were classed as “secondary reports.” Therefore, the primary reports are all unique trials

**Fig. 2:**
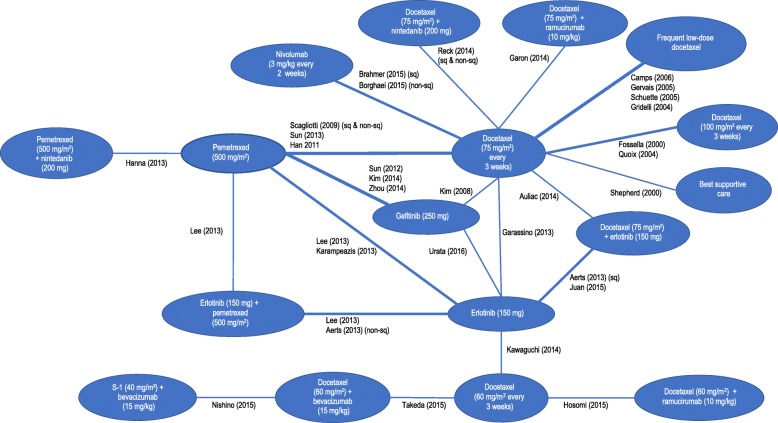
Network of Evidence for Overall Survival

**Fig. 3:**
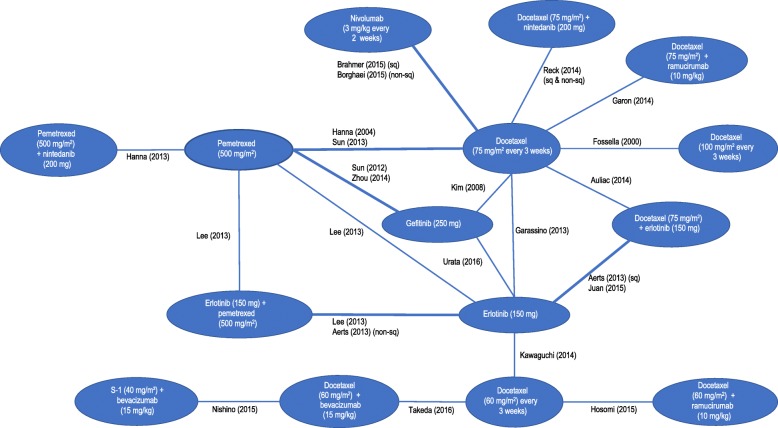
Network of Evidence for Progression-Free Survival

We screened 2601 records and 30 trials evaluating 17 interventions for inclusion in the NMA (Fig. 1 and Table 1). Characteristics of included studies are provided in Table 1. The network diagrams for OS and PFS are shown in Figs. 2 and 3, respectively. A bias assessment of the included studies is presented in Additional file 1: Table S1. The bias assessment showed that a high proportion of studies were open label, with physicians and patients not blinded to treatment. An additional cause of concern was treatment switching in the study by Kawaguchi et al. [46], which was the only study that connected docetaxel (60 mg/m^2^) to the rest of the network. This study used erlotinib (150 mg) as the comparator in a patient population with 16% EGFR mutation-positive tumors in the erlotinib arm and 25% in the docetaxel arm. The treatment switching, therefore, was likely to have caused confounding by potentially increasing the perceived relative efficacy for docetaxel (60 mg/m^2^), although the use of constraints prevented this reversal from being observed. The data for OS, including hazard ratios, median survival times, and covariate data, are presented in Additional file 1: Table S2. The covariate data from this table shows some differences between studies for the proportion of patients restricted in activity (performance status (ECOG) ≥ 1 = 0.48–0.94), stage of disease (stage IV = 0.53–1.0), and proportion of Asian patients (0.01–1.0); although none of these covariates appeared to improve the fit of the NMAs.

**Table 1:** Characteristics of included studies in the network of evidence

Study	Restriction	Interventions	N
Aerts et al. (2013) [49] (NVALT-10)	Nonsquamous histology	Erlotinib (150 mg)	73
		Erlotinib (150 mg) + pemetrexed (500 mg/m^2^)	82
Aerts et al. (2013) [49] (NVALT-10)	Squamous histology	Erlotinib (150 mg)	42
		Docetaxel (75 mg/m^2^) + erlotinib (150 mg)	34
Auliac et al. (2014) [50]	EGFR mutation negative or unknown	Docetaxel (75 mg/m^2^)	74
		Docetaxel (75 mg/m^2^) + erlotinib (150 mg)	73
Borghaei et al. (2015) [39] (CheckMate 057)	Not restricted	Docetaxel (75 mg/m^2^)	224
		Nivolumab 3 mg/kg	231
Brahmer et al. (2015)e [40] (CheckMate 017)	Not restricted	Docetaxel (75 mg/m^2^)	108
		Nivolumab 3 mg/kg	117
Camps et al. (2006) [51]	Not restricted	Docetaxel (75 mg/m^2^)	129
		Frequent low-dose docetaxel	125
Fossella (2000) [52]	Not restricted	Docetaxel (75 mg/m^2^)	125
		Docetaxel (100 mg/m^2^)	125
Garassino et al. (2013) [53] (TAILOR)	EGFR mutation negative	Docetaxel (75 mg/m^2^)	110
		Erlotinib 150 mg	109
Garon et al. (2014) [54] (REVEL)	Not restricted	Docetaxel (75 mg/m^2^)	618
		Docetaxel (75 mg/m^2^) + ramucirumab (10 mg/kg)	622
Gervais et al. (2005) [55]	Not restricted	Docetaxel (75 mg/m^2^)	62
		Frequent low-dose docetaxel	63
Gridelli et al. (2004) [56] (DISTAL 01)	Not restricted	Docetaxel (75 mg/m^2^)	110
		Frequent low-dose docetaxel	110
Han et al. (2011) [57]	Not restricted	Docetaxel (75 mg/m^2^)	40
		Pemetrexed (500 mg/m^2^)	44
Hanna et al. (2004) [58]; Scagliotti et al. (2009) [37]	Not restricted	Docetaxel (75 mg/m^2^)	288
		Pemetrexed (500 mg/m^2^)	283
Hanna et al. (2013) [59] (LUME-Lung 2)	Not restricted	Pemetrexed (500 mg/m^2^)	360
		Pemetrexed (500 mg/m^2^) + nintedanib (200 mg)	353
Hosomi et al. (2015) [60]	Asian population	Docetaxel (60 mg/m^2^)	81
		Docetaxel (60 mg/m^2^) + ramucirumab (10 mg/kg)	76
Juan et al. (2015) [61]	Not restricted	Erlotinib (150 mg)	35
		Docetaxel (75 mg/m^2^) + erlotinib (150 mg)	33
Karampeazis et al. (2013) [62] (NCT00440414)	Not restricted	Erlotinib (150 mg)	39
		Pemetrexed (500 mg/m^2^)	36
Katakami et al. (2014) [63] / Urata et al. (2016) [35]	Asian population	Erlotinib (150 mg)	252
		Gefitinib (250 mg)	250
Kawaguchi et al. (2014) [46] (DELTA)	Asian population	Erlotinib (150 mg)	150
		Docetaxel (60 mg/m^2^)	151
Kim et al. (2008) [64]	Not restricted	Docetaxel (75 mg/m^2^)	710
		Gefitinib (250 mg)	723
Kim et al. (2014) [65]	Asian population	Pemetrexed (500 mg/m^2^)	45
		Gefitinib (250 mg)	43
Lee et al. (2013) [66]	Not restricted	Erlotinib (150 mg)	82
		Pemetrexed (500 mg/m^2^)	77
		Erlotinib (150 mg) + pemetrexed (500 mg/m^2^)	75
Nishino et al. (2015) [67]	Nonsquamous histology Asian population	Docetaxel (60 mg/m^2^) + bevacizumab (15 mg/kg)	45
		S-1 (40 mg/m^2^) + bevacizumab (15 mg/kg)	45
Quoix et al. (2004) [68]	Not restricted	Docetaxel (75 mg/m^2^)	93
		Docetaxel (100 mg/m^2^)	89
Reck et al. (2014) [38] (LUME-Lung 1)	Not restricted	Docetaxel (75 mg/m^2^)	615
		Docetaxel (75 mg/m^2^) + nintedanib (200 mg)	598
Schuette et al. (2005) [69]	Not restricted	Docetaxel (75 mg/m^2^)	103
		Frequent low-dose docetaxel	105
Shepherd et al. (2000) [70]	Not restricted	Docetaxel (75 mg/m^2^)	55
		Best supportive care	49
Sun et al. (2012) [34] (KCSG-LU08–01)	Adenocarcinoma histology Asian population	Pemetrexed (500 mg/m^2^)	67
		Gefitinib (250 mg)	68
Sun et al. (2013) [71] (JMID)	Asian population	Docetaxel (75 mg/m^2^)	98
		Pemetrexed (500 mg/m^2^)	104
Takeda et al. (2015;2016) [72, 73]	Nonsquamous histology Progressed after treatment with bevacizumab plus a platinum-based doublet. Asian population	Docetaxel (60 mg/m^2^)	50
		Docetaxel (60 mg/m^2^) + bevacizumab (15 mg/kg)	50
Zhou et al. (2013;2014) [74, 75]	Nonsquamous histology EGFR mutation negative Asian population	Pemetrexed (500 mg/m^2^)	76
		Gefitinib (250 mg)	81

The study designs and reporting of trials studying PD-L1 immunotherapies also was an issue. Brahmer et al. [40] and Borghaei et al. [39] studied nivolumab versus docetaxel in patient populations without any restriction on PD-L1 expression and presented the results for < 1% versus ≥1%, < 5% versus ≥5%, and < 10% versus ≥10%. However, Herbst et al. [18] included only patients with PD-L1 expression of ≥1% and results were stratified as 1 to 49% and ≥ 50%. The approach undertaken for the NMA relied on being able to estimate the efficacy of regimens for the full range of patient types; in the absence of information on how pembrolizumab performed in a low PD-L1 expression population, the Herbst study was excluded from the NMA. This left us with the choice for which of the cut-offs presented by Brahmer et al. [40] and Borghaei et al. [39] to present in this study. The results for the 5 and 10% cut-offs were closer to each other and appeared to be more consistent than the 1% cut-off results, i.e., efficacy improved for both OS and PFS, with higher PD-L1 expression values for both studies, for the 5 and 10% cut-offs, whereas the 1% cut-off results reported by Brahmer et al. [40] appeared to show a reversal in this pattern, although this was not statistically significant. The 5% cut-off data therefore were selected because they represented the mid cut-off presented and appeared to give more consistent results. The overall results in which nivolumab showed a significant benefit over docetaxel (150 mg) were not affected by the choice between the 1 and 5% cut-offs for either the fixed-effects or random-effects models for OS and PFS.

### Proportional hazards, heterogeneity and inconsistency, and modeling issues

An assessment of the proportional hazards assumption and heterogeneity and inconsistency is provided in the Additional file 1. The assessment indicated that the proportional hazards assumption was mainly an issue for PFS (2 out of 32 studies had nonproportional hazards for OS, and 7 out of 29 studies had nonproportional hazards for PFS) and that the exchangeable hierarchical structures were needed for all the NMAs fitted. The main evidence for the difference in efficacy of TKIs in the network of evidence came from the subgroup data presented by Urata et al. [35]. For OS, these data were presented only as median survival times compared to all Kaplan-Meier estimates available for PFS. The fitting of fractional polynomial models for OS was problematic because these models were not able to differentiate between the efficacy of TKIs for EGFR mutation-negative and mutation-positive tumor types. However, the hazard ratio NMAs were able to demonstrate a difference in efficacy of TKIs by EGFR status; and because the evidence for nonproportional hazards for OS was weak, the results presented for OS are those based on the hazard ratio NMA. The hazard ratios for OS were applied to fractional polynomial model fitted to the reference treatment (docetaxel 75 mg/m^2^) from the network of evidence. Details of this model are given in the Additional file 1 and predicted survival curves by tumor histology presented in Additional file 1: Figure S1. The fractional polynomial model for PFS did not have these issues, and so this was the model used to present PFS results. For both these NMAs, the random-effects models did not improve model fit over the fixed-effects results; only the fixed-effects results are presented in the main body of the text; random-effects results are presented in Additional file 1: Tables S3 and S4 for OS and PFS, respectively, for more details.

The fitting of fractional polynomial models to multiple treatment data can result in survival curves that flatten before reaching zero, especially for second-order models with time-varying treatment effects. (All predictions by treatment are shown in Additional file 1: Figure S2.) This was observed in the NMAs fitted using this technique. The hazard rates therefore were estimated up to the end of follow-up for the available evidence by intervention; after this point had been reached, the hazard rates from the reference treatment were used.

### Presentation of results

The results for the eight subgroups are presented in the following subsections and in the Additional file 1. Only main figures for the subgroup representing the largest population (nonsquamous, EGFR mutation negative, and PD-L1 expression < 5%) are presented in the manuscript. The full set of results for each subgroup can be in found in the Additional file 1: Figures S3–S48). The Additional file 1 provides survival probability curves, mean survival times, and relative differences in mean survival for OS and PFS, by subgroup. A summary table for all the results by subgroups is provided in Table 2. SUCRA scores are not presented due to apparent bias with this network of evidence and because they were found to not be consistent with relative differences and credible intervals. This was particularly the case for treatments represented by a small sample size and located on the periphery of the network of evidence.

**Table 2:** Summary of interventions that showed a significant (*P* < 0.05) benefit over single-agent docetaxel (75 mg/m^2^): fixed-effects NMA

Histology	PD-L1 expression	EGFR mutation	Occurrence (Non-Asian)	Occurrence (Asian)	OS (Hazard ratio NMA)	PFS (Fractional polynomial NMA)
Non-squamous	< 5%	Negative	32.8%	21.2%	Docetaxel + nintedanib: 2.6 (0.1, 5.6) Docetaxel + ramucirumab: 2.3 (0.3, 4.6)	Docetaxel + ramucirumab: 1.2 (0.6, 1.9)
Squamous	< 5%	Negative	21.0%	20.2%	Nivolumab: 5.5 (0.7, 12.4) Docetaxel + ramucirumab: 2.0 (0.3, 4.0)	Nivolumab: 2.6 (0.0, 5.8)^a^ Docetaxel + ramucirumab: 1.2 (0.6, 1.9)
Non-squamous	≥ 5%	Negative	20.5%	13.3%	**Nivolumab: 12.9 (5.6, 23.8)** Docetaxel + nintedanib: 2.6 (0.1, 5.6) Docetaxel + ramucirumab: 2.3 (0.3, 4.6)	**Nivolumab: 5.0 (2.2, 8.2)** Docetaxel + ramucirumab: 1.2 (0.6, 1.9)
Squamous	≥ 5%	Negative	13.2%	12.7%	Nivolumab 8.0 (1.6, 17.8) Docetaxel + ramucirumab: 2.0 (0.3, 4.0)	**Nivolumab: 5.7 (1.8, 10.1)** Docetaxel + ramucirumab: 1.2 (0.6, 1.9)
Non-squamous	< 5%	Positive	7.2%	18.8%	**Docetaxel + erlotinib: 13.4 (4.8, 27.0)** Erlotinib + pemetrexed: 8.0 (0.3, 28.5) Erlotinib: 7.4 (2.5, 14.9) Gefitinib: 4.4 (0.8, 10.5) Docetaxel + nintedanib: 2.6 (0.1, 5.6) Docetaxel + ramucirumab: 2.3 (0.3, 4.6)	**Docetaxel + erlotinib: 8.1 (4.9, 10.9)** Erlotinib + pemetrexed: 7.0 (1.2, 14.6) **Erlotinib: 6.8 (3.4, 11.3)** **Gefitinib: 5.4 (2.7, 8.6)** Docetaxel + ramucirumab: 1.2 (0.6, 1.9)
Squamous	< 5%	Positive	0.5%	1.3%	**Docetaxel + erlotinib: 11.9 (4.2, 23.8)** Erlotinib: 6.5 (2.2, 13.2) Nivolumab: 5.5 (0.7, 12.4) Gefitinib: 3.9 (0.7, 9.3) Docetaxel + ramucirumab: 2.0 (0.3, 4.0)	**Docetaxel + erlotinib: 8.1 (4.9, 10.9)** **Erlotinib: 6.8 (3.4, 11.3)** **Gefitinib: 5.4 (2.7, 8.6)** Nivolumab: 2.6 (0.0, 5.8)^a^ Docetaxel + ramucirumab: 1.2 (0.6, 1.9)
Non-squamous	≥ 5%	Positive	4.5%	11.8%	**Docetaxel + erlotinib: 13.4 (4.8, 27.0)** **Nivolumab: 12.9 (5.6, 23.8)** Erlotinib + pemetrexed: 8.0 (0.3, 28.5) Erlotinib: 7.4 (2.5, 14.9) Gefitinib: 4.4 (0.8, 10.5) Docetaxel + nintedanib: 2.6 (0.1, 5.6) Docetaxel + ramucirumab: 2.3 (0.3, 4.6)	**Docetaxel + erlotinib: 8.1 (4.9, 10.9)** Erlotinib + pemetrexed: 7.0 (1.2, 14.6) **Erlotinib: 6.8 (3.4, 11.3)** **Gefitinib: 5.4 (2.7, 8.6)** **Nivolumab: 5.0 (2.2, 8.2)** Docetaxel + ramucirumab: 1.2 (0.6, 1.9)
Squamous	≥ 5%	Positive	0.3%	0.8%	**Docetaxel + erlotinib: 11.9 (4.2, 23.8)** Nivolumab 8.0 (1.6, 17.8) Erlotinib: 6.5 (2.2, 13.2) Gefitinib: 3.9 (0.7, 9.3) Docetaxel + ramucirumab: 2.0 (0.3, 4.0)	**Docetaxel + erlotinib: 8.1 (4.9, 10.9)** **Erlotinib: 6.8 (3.4, 11.3)** **Nivolumab: 5.7 (1.8, 10.1)** **Gefitinib: 5.4 (2.7, 8.6)** Docetaxel + ramucirumab: 1.2 (0.6, 1.9)

Docetaxel = docetaxel (75 mg/m^2^) 3 times a week; difference in mean survival relative to docetaxel (75 mg/m^2^) after colon with 95% credible intervals in parentheses. Treatments shown in bold indicate relatively better performance in a group; (typically, the highest predicted mean survival) and performed better than one or more other treatments in the same group (*P* < 0.05). Occurrence of each tumor subgroup are only approximate and based on the following: 65% nonsquamous, 35% squamous [5]; non-Asian: 18% EGFR mutation positive in nonsquamous tumors; Asian: 47% EGFR mutation in nonsquamous tumors [76]; 8 times more likely to be EGFR positive if nonsquamous compared to squamous [77] and 38.5% PD-L1 ≥ 5% (combined data from Borghaei et al. [39]; Brahmer et al. [40]). Predictions from the NMA assumed relationships for each factor are the same across any other factor. This allowed predictions to be made across all subgroups, but where subgroups are rare, there may be little actual direct evidence for that patient population

^a^Borderline significance (*P* = 0.0508)

### Results by subgroup

#### Nonsquamous, PD-L1 expression < 5% and EGFR negative

**Fig. 4:**
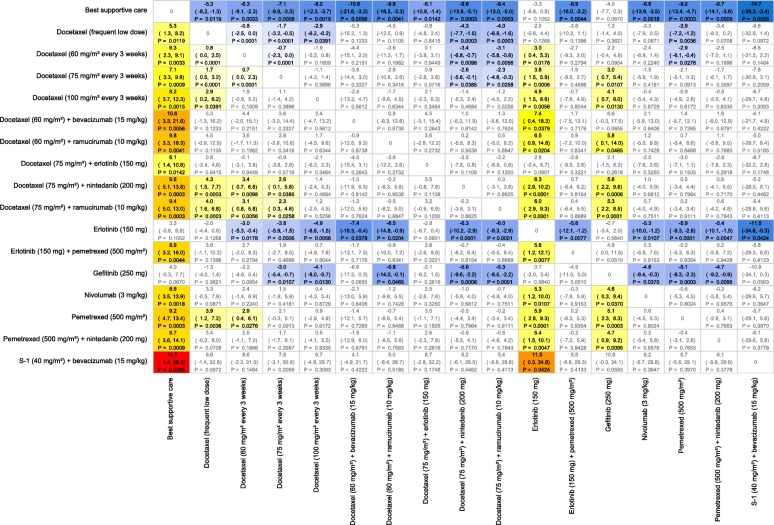
All Pairwise Difference in Predicted Mean Overall Survival Times for Nonsquamous, EGFR Mutation Negative, PD-L1 <  5%. Notes: Differences defined as row treatment minus column treatment with 95% Credible Intervals and probability of significant difference. Colors represent a “heat map” with blues representing large negative differences, increasing through to dark reds for large positive mean differences

**Fig. 5:**
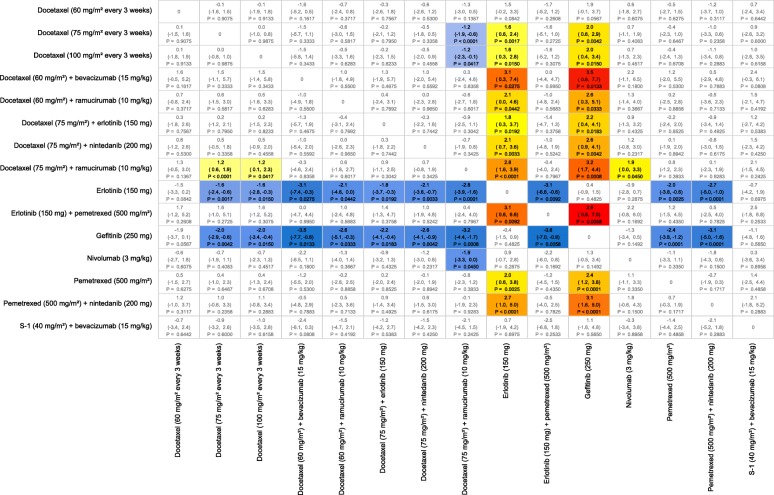
All Pairwise Difference in Predicted Mean Progression-Free Survival Times for Nonsquamous, EGFR Mutation Negative, PD-L1 <  5%. Notes: Differences defined as row treatment minus column treatment with 95% Credible Intervals and probability of significant difference. Colors represent a “heat map” with blues representing large negative differences, increasing through to dark reds for large positive mean differences

For OS, docetaxel plus ramucirumab and docetaxel plus nintedanib were the only interventions that showed a significant improvement in mean survival time over docetaxel (75 mg/m^2^) with gains of 2.3 and 2.6 months, respectively (Fig. 4). Survival curves and mean OS times are shown in Additional file 1: Figure S3. For PFS, only docetaxel plus ramucirumab gave a significant benefit, of 1.2 months, in mean survival (Fig. 5). Mean PFS survival times are presented in Additional file 1: Figure S6.

#### Squamous, PD-L1 expression < 5% and EGFR negative

For OS, docetaxel plus ramucirumab and nivolumab were the only interventions that showed a significant improvement in mean survival over docetaxel (75 mg/m^2^) with a gain of 2.0 and 5.5 months, respectively (Additional file 1: Figures S7–S9). For PFS, only docetaxel plus ramucirumab gave a significant benefit, of 1.2 months, in mean survival, and nivolumab showed borderline significance (*P* = 0.0508) with 2.6 months (Additional file 1: Figures S10–S12).

#### Nonsquamous, PD-L1 expression ≥5% and EGFR negative

For OS, nivolumab, docetaxel plus ramucirumab and docetaxel plus nintedanib showed a significant improvement in mean survival over docetaxel (75 mg/m^2^) with gains of 12.9, 2.3, and 2.6 months, respectively (Additional file 1: Figures S13–S15). For PFS, only nivolumab and docetaxel plus ramucirumab gave a significant benefit of 5.0 and 1.2 months, respectively (Additional file 1: Figures S16–S18). Nivolumab showed significant superiority over docetaxel plus ramucirumab and docetaxel plus nintedanib (*P* < 0.05) for OS and PFS.

#### Squamous, PD-L1 expression ≥5% and EGFR negative

For OS, nivolumab and docetaxel plus ramucirumab were the only interventions to show a significant improvement in mean survival over docetaxel (75 mg/m^2^), with a gain of 8.0 and 2.0 months, respectively (Additional file 1: Figures S19–S21). For PFS, nivolumab and docetaxel plus ramucirumab also showed statistically significant benefits of 5.7 and 1.2 months, respectively (Additional file 1: Figures S22–S24). Nivolumab showed significant superiority over docetaxel plus ramucirumab (*P* < 0.05) for PFS.

#### Nonsquamous, PD-L1 expression < 5% and EGFR positive

For OS, the following treatments showed a significant gain over docetaxel (75 mg/m^2^) (difference in months for mean survival shown in parentheses): docetaxel plus erlotinib (13.4), erlotinib plus pemetrexed (8.0), erlotinib (7.4), gefitinib (4.4), docetaxel plus nintedanib (2.6), and docetaxel plus ramucirumab (2.3) (Additional file 1: Figures S25–S27). For PFS, the following treatments showed a significant gain over docetaxel (75 mg/m^2^) (difference in months for mean survival shown in parentheses): docetaxel plus erlotinib (8.1), erlotinib plus pemetrexed (7.0), erlotinib (6.8), gefitinib (5.4), and docetaxel plus ramucirumab (1.2) (Additional file 1: Figures S28–S30). Docetaxel plus erlotinib showed significant superiority (*P* < 0.05) over gefitinib and non-TKI regimens for OS. Docetaxel plus erlotinib, erlotinib, and gefitinib showed significant superiority (*P* < 0.05) over docetaxel plus ramucirumab for PFS.

#### Squamous, PD-L1 expression < 5% and EGFR positive

For OS, the following treatments showed a significant gain over docetaxel (75 mg/m^2^) (difference in months for mean survival shown in parentheses): docetaxel plus erlotinib (11.9), erlotinib (6.5), nivolumab (5.5), gefitinib (3.9), and docetaxel plus ramucirumab (2.0) (Additional file 1: Figures S31–S33). For PFS, the following treatments showed a significant gain over docetaxel (75 mg/m^2^) (difference in months for mean survival shown in parentheses): docetaxel plus erlotinib (8.1), erlotinib (6.8), gefitinib (5.4), and docetaxel plus ramucirumab (1.2) (Additional file 1: Figures S34–S36). Nivolumab showed borderline significance with an increase of 2.6 months. Docetaxel plus erlotinib showed significant superiority (*P* < 0.05) over gefitinib and docetaxel plus ramucirumab for OS. Docetaxel plus erlotinib showed significant superiority (*P* < 0.05) over non-TKI regimens for PFS.

#### Nonsquamous, PD-L1 expression ≥5% and EGFR positive

For OS, the following treatments showed a significant gain over docetaxel (75 mg/m^2^) (difference in months for mean survival shown in parentheses): docetaxel plus erlotinib (13.4), nivolumab (12.9), erlotinib plus pemetrexed (8.0), erlotinib (7.4), gefitinib (4.4), docetaxel plus nintedanib (2.6), and docetaxel plus ramucirumab (2.3) (Additional file 1: Figures S37–S39). For PFS, the following treatments showed significant gains over docetaxel (75 mg/m^2^) (difference in months for mean survival shown in parentheses): docetaxel plus erlotinib (8.1), erlotinib plus pemetrexed (7.0), erlotinib (6.8), gefitinib (5.4), nivolumab (5.0), and docetaxel plus ramucirumab (1.2) (Additional file 1: Figures S40–S42). Docetaxel plus erlotinib and nivolumab showed significant superiority (*P* < 0.05) over docetaxel plus ramucirumab for OS. Docetaxel plus erlotinib, erlotinib, gefitinib, and nivolumab showed significant superiority (*P* < 0.05) over docetaxel plus ramucirumab for PFS.

#### Squamous, PD-L1 expression ≥5% and EGFR positive

For OS, the following treatments showed significant gains over docetaxel (75 mg/m^2^) (difference in months for mean survival shown in parentheses): docetaxel plus erlotinib (11.9), nivolumab (8.0), erlotinib (6.5), gefitinib (3.9), and docetaxel plus ramucirumab (2.0) (Additional file 1: Figures S43–S45). For PFS, the following treatments showed significant gains over docetaxel (75 mg/m^2^) (difference in months shown in parentheses): docetaxel plus erlotinib (8.1), erlotinib (6.8), nivolumab (5.7), gefitinib (5.4), and docetaxel plus ramucirumab (1.2) (Additional file 1: Figures S46–S48). Docetaxel plus erlotinib showed significant superiority (*P* < 0.05) over docetaxel plus ramucirumab for OS. Docetaxel plus erlotinib, erlotinib, nivolumab, and gefitinib showed significant superiority (*P* < 0.05) over non-TKI regimens and non-PD-1 immunotherapies for PFS.

#### Summary of results by subgroup

Table 2 summarizes all the interventions that showed a significant gain over docetaxel (75 mg/m^2^) by subgroup, including the difference in mean survival. Sensitivity analyses for random-effects models also were performed, and these are presented in Additional file 1: Table S5. These tables show how the optimal treatments change with each subgroup. TKI based regimens consistently showed the greatest benefit over docetaxel (75 mg/m^2^) in patients with EGFR mutation positive tumor types regardless of histology. However, in the PD-L1 high expression group nivolumab showed a similar benefit to the TKI based regimens in patients with EGFR mutation positive tumor types. For the nonsquamous, low PD-L1 expression EGFR mutation negative subgroups, ramucirumab plus docetaxel and nintedanib plus docetaxel provided the greatest benefit. Combination treatments such as docetaxel plus erlotinib appeared to give the greatest benefit in certain subgroups but there was a greater uncertainty in these predictions which was reflected in the large credible intervals.

## Discussion

The results from this study provide further evidence that it is important to appropriately target specific treatments for particular patient groups to optimize outcomes. The mean survival times reported in this study also were in agreement with previous NMAs. The improvement in OS, using the hazard ratio model applied to a fractional polynomial model for the reference treatment in this study, matched closely those reported by Jansen [12] for a second-order fractional polynomial model fitted to reconstructed data for results where patient groups were not a factor (docetaxel vs. best supportive care: Jansen [12], 6.8 months; this study, 7.1 months for nonsquamous and 6.3 months for squamous); mean OS for docetaxel: Jansen [12], 13.0 months; this study, nonsquamous, 14.2 months and squamous, 12.7 months). The fractional polynomial models fitted to OS, which were not presented, also showed comparable mean survival times with those from the hazard ratio model, with the exception of the TKIs.

The main strengths of this study were that data were identified by a systematic literature review and that model flexibility allowed particular treatments to vary by histology, PD-L1 expression, and EGFR mutation. In addition, the survival curves for the reference treatment were validated using the data from Penrod et al. [47]. Heterogeneity and inconsistency were thoroughly investigated using pairwise meta-analyses of duplicate comparisons and node-splitting. The inclusion of hierarchical exchangeable structures in the models appeared to have sufficiently explained the heterogeneity and inconsistency observed to the point where fixed-effects models produced better goodness of fit statistics than random-effects models. This is the first study we know of that models the efficacy of interventions for NSCLC by histology, PD-L1 expression, and EGFR mutation status within an NMA. Some of these interactions have been only recently discovered, and it is likely that our understanding will continue to increase as more research is conducted.

The main study limitation was the variable length of follow-up time between studies, which meant there was some uncertainty in the extrapolation of OS and PFS survival curves. For OS, the hazard ratios models were simply applied to the reference model by histology, so treatment effects were assumed to continue after follow-up. However, to ensure PFS survival curves were plausible, the hazard rates from the reference treatment were used once the time exceeded the maximum follow-up data for a given intervention. Interventions with shorter follow-up times but that appeared to have lower hazard rates than the reference treatments therefore may have been at a disadvantage. However, the approach used in this study did make full use of the available data. The proportion of patients tested for EGFR mutation status varied across studies and may have been a source of bias. EGFR mutation also covers different genetic markers and other factors, such as EGFR copy number, although these may not be independent predictive markers [32]. The predictions for combination treatments were partly based on extrapolation from single-interventions results, so there is additional uncertainty around these estimates. This uncertainty mainly concerns docetaxel plus erlotinib because the studies that included this regimen contained low proportions of EGFR mutation-positive patients. The increased survival benefit predicted for patients receiving docetaxel plus erlotinib who have tumors that are EGFR mutation positive was based on the associated efficacy of erlotinib monotherapy with EGFR mutation status. Assumptions were also needed to make predictions for each of the tumor subtypes. Some studies reported results for nonsquamous tumors and squamous whilst other studies were restricted to adenocarcinoma. For this study, we assumed that nonsquamous and adenocarcinoma were equivalent to each other. Some of the tumor subtypes are rare such as squamous tumors that are EGFR mutation positive, and because only study level covariate data were reported we do not know whether all patient types existed in the data analyzed. The association of a change in efficacy for a particular treatment with a tumor characteristic was assumed to hold across all tumor types. Some caution may therefore be needed in interpreting the predictions for the rarer subgroups or where a tumor characteristic such as PD-L1 expression was only reported in a small number of studies because, for this example, only comparative data for nivolumab were presented by PD-L1 expression.

There were inconsistencies between the results for OS and PFS for nintedanib, nivolumab, and S-1 plus bevacizumab, which makes it difficult to be certain of the relative effectiveness of these interventions. The efficacy of docetaxel plus nintedanib and nivolumab were allowed to vary by histology in this study, although the actual trial results for these interventions had not shown there was a significant association with histology (e.g., for nintedanib plus docetaxel: OS, *P* = 0.1194; PFS, *P* = 0.4700, as stated by Reck et al. [38]). If the efficacy of these regimens had not been allowed to vary by histology, then the efficacy found for nintedanib would have been poorer than the results presented from this study for the nonsquamous subgroup for OS, and also we may not have seen a significant result for nivolumab in the squamous PD-L1 expression population. The evidence for S-1 and bevacizumab was weak in the network studied. This was partly due to small sample sizes and also due to the distance between these treatments and the reference treatment in the network of evidence. It is therefore difficult to draw conclusions for these treatments, especially considering that the results for OS and PFS gave conflicting results. It is also worth noting that some treatments gave a significant change in median survival when the mean survival was not significant, which can be found by viewing the survival curves and looking at the credible intervals at 50% survival. This was the case for docetaxel plus nintedanib for PFS of both nonsquamous and squamous tumor subtypes, for docetaxel plus nintedanib for the random-effects NMA for PFS of the nonsquamous tumor subtype, and for docetaxel plus ramucirumab for the random-effects NMA for PFS. The focus of mean survival times in this study is therefore considered to be more conservative than if the focus had been on median survival times.

A further more general limitation with NMAs conducted in NSCLC is the rapidly changing landscape of possible therapies. For example, the results from studies of two more PD-1 immunotherapies have been published since the completion of the literature review conducted for our study. Herbst et al. [18] presented the results for pembrolizumab versus docetaxel, and Fehrenbacher et al. [48] presented the results for atezolizumab versus docetaxel. Where comparable data exists for these two studies and for the nivolumab study, they are presented in Additional file 1: Table S6. A more thorough comparison of these treatments has been presented by the Institute for Clinical and Economic Review [14]. The evidence suggested that nivolumab (3 mg/kg), pembrolizumab (10 mg/kg), and atezolizumab (1200 mg) show similar efficacy. The evidence for whether nivolumab is effective in patients with squamous NSCLC who have PD-L1 expression of < 1% or <  5% appears to be inconclusive. Also, it should be noted that some of the included treatments or combinations may not be approved in specific markets or for specific subpopulations of interest in this study and that some treatments may not be used or recommended to be used in real-world clinical practice. Another limitation is that this study does not address the relative improvements in quality of life compared to survival time or toxicity.

## Conclusions

In conclusion, the overall trends across OS and PFS indicated that there was always at least one intervention that performed better than single-agent docetaxel. Docetaxel plus ramucirumab gave a consistent significant benefit across all NSCLC subtypes. Docetaxel plus nintedanib showed a similar efficacy to docetaxel plus ramucirumab in the nonsquamous population. Superiority was observed for regimens containing erlotinib or gefitinib compared to non-TKI regimens when used in patients whose tumors have EGFR mutations, which was expected given the evidence in the literature [17, 32–35]. For patients whose tumors had a PD-L1 expression of ≥5%, superiority was observed in those patients who received nivolumab compared to non-PD-L1 immunotherapies. This was particularly evident for patients with nonsquamous NSCLC and PD-L1 expression ≥5%. It was not clear whether this was generally the case for patients whose tumor had high PD-L1 expression or whether nivolumab was effective across squamous tumor types, regardless of PD-L1 expression. There was insufficient evidence available to assess bevacizumab and S-1.

New treatments for NSCLC are being developed and studied; these treatments often are specific to particular biomarkers. This will add further complexity to NMAs conducted in this disease area. However, the results from this study should help inform the decision-making process for prescribing currently available treatments and could be used to help power future trials. Results also may be used to serve as a reference for the efficacy of existing treatments for patients with a particular tumor type, where only mixed population evidence so far exists. As far as we know, this is the only NMA in which investigators have attempted to model the treatment covariate interactions present in NSCLC for second-line treatment after disease progression has occurred.

## Additional file

Additional file 1: Risk of bias assessment, study characteristics, sensitivity analyses, subgroup results and Bayesian code. (DOCX 3820 kb)

## Abbreviations

ECOG: Eastern Cooperative Oncology Group
EGFR: Epidermal growth factor receptor
NMA: Network meta-analysis
NSCLC: Non-small cell lung cancer
OS: Overall survival
PD-L1: Programmed-death ligand-1
PFS: Progression-free survival
PRISMA: Preferred Reporting Items for Systematic Reviews and Meta-Analyses
RCT: Randomized clinical trials
SUCRA: Surface under the cumulative ranking
TKI: Tyrosine kinase inhibitors
