# Supplementary material for: Relative efficacy of interventions in the treatment of second-line non-small cell lung cancer: a systematic review and network meta-analysis
Source: BMC Cancer. 2019 Apr 15;19:353. doi: 10.1186/s12885-019-5569-5 (PMC6466705; doi:10.1186/s12885-019-5569-5)
Supplement: Supplementary file 1 — Risk of bias assessment, study characteristics, sensitivity analyses, subgroup results and Bayesian code. (DOCX 3820 kb) [file 12885_2019_5569_MOESM1_ESM.docx]

Additional file 1

Contents

[Tables of Data 2](#_Toc495931225)

[Proportional Hazards 9](#_Toc495931226)

[Heterogeneity and Inconsistency 9](#_Toc495931227)

[Model Fit Statistics 10](#_Toc495931228)

[Bayesian Fractional Polynomial Model Fitted to the Reference Treatment for Overall Survival 17](#_Toc495931229)

[Bayesian Fractional Polynomial Model Fitted to the Network of Evidence for Progression-Free Survival 19](#_Toc495931230)

[Results for Nonsquamous, PD-L1 Expression < 5%, EGFR Mutation Negative 21](#_Toc495931231)

[Results for Squamous, PD-L1 Expression < 5%, EGFR Mutation Negative 26](#_Toc495931232)

[Results for Nonsquamous, PD-L1 Expression ≥ 5%, EGFR Mutation Negative 33](#_Toc495931233)

[Results for Squamous, PD-L1 Expression ≥ 5%, EGFR Mutation Negative 40](#_Toc495931234)

[Results for Nonsquamous, PD-L1 Expression < 5%, EGFR Mutation Positive 47](#_Toc495931235)

[Results for Squamous, PD-L1 Expression < 5%, EGFR Mutation Positive 54](#_Toc495931236)

[Results for Nonsquamous, PD-L1 Expression ≥ 5%, EGFR Mutation Positive 61](#_Toc495931237)

[Results for Squamous, PD-L1 Expression ≥ 5%, EGFR Mutation Positive 68](#_Toc495931238)

[JAGS Code Used for the Reference Treatment for Overall Survival: First-Order Fractional Polynomial Survival Model With Random Effects for Shape and Scale and Correlation Between These Parameters 75](#_Toc495931239)

[JAGS Code Used for Overall Survival: Fixed-Effects Hazard Ratio Model With Hierarchical Exchangeable Structures 76](#_Toc495931240)

[JAGS Code Used for Overall Survival: Random-Effects Hazard Ratio Model With Hierarchical Exchangeable Structures 79](#_Toc495931241)

[JAGS Code Used for Progression-Free Survival: Second-Order Fixed-Effects Fractional Polynomial Survival Model With Hierarchical Exchangeable Structures 82](#_Toc495931242)

[JAGS Code Used for Progression-Free Survival: Second-Order Random Scale-Effects Fractional Polynomial Survival Model With Hierarchical Exchangeable Structures 85](#_Toc495931243)

[JAGS Code Used for Progression-Free Survival: Second-Order Random Scale- and Shape- Effects Fractional Polynomial Survival Model With Hierarchical Exchangeable Structures 89](#_Toc495931244)

## Tables of Data

Table S1. Risk of Bias Assessment of Trials Included in the Network Meta-Analysis

| Study | Randomization | Concealment of Treatment Allocation Adequate | Groups Similar at Study Outset | ITT Analysis Included | Unexpected Imbalances in Dropouts Between Groups | Selective Reporting |
| --- | --- | --- | --- | --- | --- | --- |
| Aerts (2013) [49] | Yes | No | Yes | Yes | No | No |
| Auliac (2014) [50] | Yes | No | Yes | Yes | No | No |
| Borghaei et al. (2015) [39] (CheckMate 057) | Yes | No | Yes | Yes | No | No |
| Brahmer et al. (2015) [40] (CheckMate 017) | Yes | No | Yes | Yes | No | No |
| Camps (2006) [51] | Yes | No | Yes | Yes | No | No |
| Fossella (2000) [52] | Yes | No | Yes | Yes | No | No |
| Garassino (2013) [53] | Yes | No | Not clear | Yes | Not clear | No |
| Garon (REVEL) [54] | Yes | Yes | Yes | Yes | No | No |
| Gervais (2005) [55] | Yes | No | Yes | Yes | No | No |
| Gridelli et al. (2004) [56] (DISTAL 01) | Yes | No | Yes | Yes | No | No |
| Han et al. (2011) [57] | Yes | Not clear | Yes | Yes | No | No |
| Hanna et al. (2004) [58]; Scagliotti et al. (2009) [37] | Yes | No | Yes | Yes | No | No |
| Hanna et al. (2013) [59] (LUME-Lung 2) | Yes | Yes | Yes | Yes | No | No |
| Hosomi et al. (2015) [60] | Yes | Yes | Yes | Yes | No | No |
| Juan et al. (2015) [61] | Yes | No | Yes | Yes | No | No |
| Karampeazis et al. (2013) [62] (NCT00440414) | Yes | No | Yes | Yes | Treatment switching occurred in both arms (19% and 4%) | No |
| Katakami et al. (2014) [63] / Urata et al. (2016) [35] | Yes | No | Yes | Yes | No | No |
| Kawaguchi et al. (2014) (DELTA) [46] | Yes | No | Yes | Yes | Treatment switching occurred in both arms (40% and 40%) | No |
| Kim et al. (2008) [64] | Yes | No | Yes | Yes | No | No |
| Kim et al. (2014) [65] | Yes | No | Not clear | Yes | Not clear | No |
| Lee et al. (2013) [66] | Yes | No | Yes | Yes | No | No |
| Nishino et al. (2015) [67] | Yes | Not clear | Yes | Yes | No | No |
| Quoix et al. (2004) [68] | Yes | No | More patients who received docetaxel 75 mg had metastatic disease than those who received 100 mg (91% vs. 79%) | Yes | No | No |
| Reck et al. (2014) [38] (LUME-Lung 1) | Yes | Yes | Yes | Yes | No | No |
| Schuette et al. (2005) [69] | Yes | No | Yes | Yes | No | No |
| Shepherd et al. (2000) [70] | Yes | No | Yes | Yes | No | No |
| Sun et al. (2012) [34] (KCSG-LU08-01) | Yes | No | Yes | Yes | No | No |
| Sun et al. (2013) [71] (JMID) | Yes | No | Yes | Yes | No | No |
| Takeda et al. (2015;2016) [72,73] | Yes | No | Yes | Yes | No | No |
| Zhou et al. (2014) [75] | Yes | No | Yes | Yes | No | No |

Table S2. Characteristics of Studies Included in the Network of Evidence

| Study | Rx1 | Rx2 | HR | OS  log(HR) | SE | Median Rx1 | Median Rx2 | n1 | n2 | Age | ECOG ≥ 1 | Stage IV | Asian | Nonsquamous^a^ | EGFR Mutation Positive^b^ |
| --- | --- | --- | --- | --- | --- | --- | --- | --- | --- | --- | --- | --- | --- | --- | --- |
| Aerts (2013) [49] non-sq | Erlotinib 150 mg + pemetrexed 500 | Erlotinib (150 mg) | 0.74 | -0.3 | 0.18 | 9.1 | 5.5 | 82 | 73 | 63 | 0.62 | 0.78 | NA | 1.00 | 0.00 |
| Aerts (2013) [49] sq | Docetaxel (75 mg/m^2^) + erlotinib (150 mg) | Erlotinib (150 mg) | 0.76 | -0.28 | 0.25 | 6.1 | 6.2 | 34 | 42 | 63 | 0.62 | 0.78 | NA | 0.00 | 0.05 |
| Auliac (2014) [50] | Docetaxel (75 mg/m^2^) + erlotinib (150 mg) | Docetaxel (75 mg/m^2^) | NA | NA | NA | 6.5 | 8.3 | 73 | 74 | 59 | 0.70 | NA | NA | 0.86 | 0.00 |
| Borghaei (2015) [39] PD-L1 < 5% | Nivolumab | Docetaxel (75 mg/m^2^) | 1.01 | 0.01 | 0.141 | 9.8 | 10.1 | 136 | 138 | 62 | 0.69 | 0.92 | 0.03 | 1.00 | 0.14 |
| Borghaei (2015) [39]  PD-L1 ≥ 5% | Nivolumab | Docetaxel (75 mg/m^2^) | 0.43 | -0.84 | 0.189 | 19.4 | 8.1 | 95 | 86 | 62 | 0.69 | 0.92 | 0.03 | 1.00 | 0.14 |
| Brahmer (2015) [40] PD-L1 < 5% | Nivolumab | Docetaxel (75 mg/m^2^) | 0.70 | -0.36 | 0.198 | NA | NA | 75 | 69 | 63 | 0.76 | 0.80 | 0.02 | 0.00 | NA |
| Brahmer (2015) [40] PD-L1 ≥ 5% | Nivolumab | Docetaxel (75 mg/m^2^) | 0.53 | -0.63 | 0.269 | NA | NA | 42 | 39 | 63 | 0.76 | 0.80 | 0.02 | 0.00 | NA |
| Camps (2006) [51] | Frequent low-dose docetaxel | Docetaxel (75 mg/m^2^) | 1.31 | 0.27 | 0.13 | 5.4 | 6.6 | 125 | 129 | 62 | 0.82 | 0.84 | NA | NA | NA |
| Fossella (2000) [52] | Docetaxel (100 mg/m^2^) | Docetaxel (75 mg/m^2^) | NA | NA | NA | 5.5 | 5.7 | 125 | 125 | 60 | NA | 0.34 | NA | 0.74 | NA |
| Garassino (2013) [53] | Erlotinib (150 mg) | Docetaxel (75 mg/m^2^) | 1.28 | 0.25 | 0.184 | NA | NA | 109 | 110 | 67 | 0.52 | NA | 0.01 | 0.76 | 0.00 |
| Garon (2014) [54] | Docetaxel (75 mg/m^2^) + ramucirumab (10 mg/kg) | Docetaxel (75 mg/m^2^) | 0.86 | -0.15 | 0.068 | NA | NA | 622 | 618 | 62 | 0.48 | 1.00 | 0.13 | 0.74 | 0.03 |
| Gervais (2005) [55] | Frequent low-dose docetaxel | Docetaxel (75 mg/m^2^) | 1.25 | 0.22 | 0.19 | 5.5 | 5.8 | 63 | 62 | 59 | 0.66 | 0.67 | NA | 0.62 | NA |
| Gridelli (2004) [56] | Frequent low-dose docetaxel | Docetaxel (75 mg/m^2^) | 1.04 | 0.04 | 0.151 | NA | NA | 110 | 110 | 60 | 0.68 | 0.84 | NA | 0.65 | NA |
| Hanna (2013) [59] | Pemetrexed (500 mg/m^2^) + nintedanib (200 mg) | Pemetrexed (500 mg/m^2^) | 1.03 | 0.03 | 0.096 | NA | NA | 353 | 360 | 60 | 0.62 | 0.64 | 0.29 | 1.00 | NA |
| Hosomi (2015) [60] | Docetaxel (60 mg/m^2^) + ramucirumab (10 mg/kg) | Docetaxel (60 mg/m^2^) | 0.77 | -0.26 | 0.242 | 15.15 | 13.93 | 76 | 81 | 65 | NA | 1.00 | 1.00 | 0.89 | NA |
| Juan (2015) [61] | Docetaxel (75 mg/m^2^) + erlotinib (150 mg) | Erlotinib (150 mg) | 0.70 | -0.36 | 0.272 | 7.5 | 5.2 | 33 | 35 | 60 | 0.94 | 0.87 | NA | 0.57 | 0.03 |
| Karampeazis (2013) [62] | Pemetrexed (500 mg/m^2^) | Erlotinib 150 | 1.00 | 0.00 | 0.129 | 10.1 | 8.2 | 36 | 39 | 66 | 0.76 | 0.91 | NA | 0.77 | 0.09 |
| Kawaguchi (2014) [46] | Docetaxel (60 mg/m^2^) | Erlotinib (150 mg) | 1.1 | 0.09 | 0.149 | 12.2 | 14.8 | 151 | 150 | 68 | 0.49 | 0.80 | 1.00 | 0.80 | 0.16 |
| Kim (2008) [64] | Gefitinib (250 mg) | Docetaxel (75 mg/m^2^) | 1.02 | 0.02 | 0.06 | 7.6 | 8 | 723 | 710 | 61 | 0.73 | 0.53 | 0.22 | 0.75 | 0.15 |
| Kim (2014) [65] | Pemetrexed (500 mg/m^2^) | Gefitinib (250 mg) | NA | NA | NA | 8.5 | 8.5 | 45 | 43 | NA | NA | NA | 1.00 | 0.81 | 0.06 |
| Lee (2013) [66] | Pemetrexed (500 mg/m^2^) | Erlotinib (150 mg) | 1.44 | 0.37 | 0.218 | NA | NA | 77 | 82 | 55 | NA | 0.86 | 0.55 | 1.00 | 0.56 |
| Lee (2013) [66] | Erlotinib (150 mg)+ pemetrexed (500 mg/m^2^) | Erlotinib (150 mg) | 1.08 | 0.08 | 0.225 | NA | NA | 75 | 82 | 55 | NA | 0.86 | 0.55 | 1.00 | 0.56 |
| Lee (2013) [66] | Erlotinib (150 mg) + pemetrexed (500 mg/m^2^) | Pemetrexed (500 mg/m^2^) | 0.75 | -0.29 | 0.213 | NA | NA | 75 | 77 | 55 | NA | 0.86 | 0.55 | 1.00 | 0.56 |
| Nishino (2015) [67] | S1_bevacizumab | Docetaxel (60 mg/m^2^) + bevacizumab | 0.80 | -0.22 | 0.274 | 21.7 | 16 | 45 | 45 | 64 | 0.71 | 0.94 | 1.00 | 1.00 | 0.32 |
| Quoix (2004) [68] | Docetaxel (100 mg/m^2^) | Docetaxel (75 mg/m^2^) | 0.73 | -0.32 | 0.16 | 6.7 | 4.7 | 89 | 93 | 59 | 0.84 | NA | NA | 0.65 | NA |
| Reck (2014) [38] non-sq | Docetaxel (75 mg/m^2^) + nintedanib (200 mg) | Docetaxel (75 mg/m^2^) | 0.83 | -0.19 | 0.088 | NA | NA | 322 | 336 | 60 | 0.71 | NA | 0.18 | 1.00 | NA |
| Reck (2014) [38] sq | Docetaxel (75 mg/m^2^) + nintedanib (200 mg) | Docetaxel (75 mg/m^2^) | 1.01 | 0.01 | 0.09 | NA | NA | 276 | 279 | 60 | 0.71 | NA | 0.18 | 0.00 | NA |
| Scagliotti (2009) [37] non-sq | Pemetrexed (500 mg/m^2^) | Docetaxel (75 mg/m^2^) | 0.78 | -0.25 | 0.126 | 9 | 9.2 | 205 | 194 | 58 | 0.81 | 0.75 | 0.17 | 1.00 | NA |
| Scagliotti (2009) [37] sq | Pemetrexed (500 mg/m^2^) | Docetaxel (75 mg/m^2^) | 1.56 | 0.45 | 0.188 | 6.2 | 7.5 | 78 | 94 | 58 | 0.81 | 0.75 | 0.17 | 0.00 | NA |
| Schuette (2005) [69] | Frequent low-dose docetaxel | Docetaxel (75 mg/m^2^) | 0.89 | -0.12 | 0.15 | 9.2 | 6.3 | 105 | 103 | 63 | 0.67 | NA | NA | 0.61 | NA |
| Shepherd (2000) [70] | Best supportive care | Docetaxel (75 mg/m^2^) | 2.08 | 0.73 | 0.229 | 4.6 | 7.5 | 49 | 55 | 61 | 0.84 | 0.77 | NA | NA | NA |
| Sun (2012) [34] | Pemetrexed (500 mg/m^2^) | Gefitinib (250 mg) | 1.25 | 0.22 | 0.24 | 18.9 | 22.2 | 67 | 68 | NA | 0.69 | 0.91 | 1.00 | 1.00 | 0.47 |
| Sun (2013) [71] | Pemetrexed (500 mg/m^2^) | Docetaxel (75 mg/m^2^) | 1.02 | 0.02 | 0.163 | NA | NA | 104 | 98 | 56 | 0.87 | 0.78 | 1.00 | 0.75 | NA |
| Takeda (2015) [72] | Docetaxel (60 mg/m^2^) + bevacizumab | Docetaxel (60 mg/m^2^) | 0.74 | -0.30 | 0.242 | 13.1 | 11 | 50 | 50 | 66 | 0.68 | 0.93 | 1.00 | 1.00 | 0.14 |
| Urata (2016) [35] e.neg_g.neg | Erlotinib (150 mg) | Gefitinib (250 mg) | NA | NA | NA | 13.08 | 13.44 | 26^c^ | 24 ^c^ | 67 | 0.55 | 0.92 | 1.00 | 1.00 | 0.00 |
| Urata (2016) [35] e.pos_g.pos | Erlotinib (150 mg) | Gefitinib (250 mg) | NA | NA | NA | 31.97 | 26.64 | 99 ^c^ | 102 ^c^ | 67 | 0.55 | 0.92 | 1.00 | 1.00 | 1.00 |
| Urata (2016) [35] e.neg_g.pos | Erlotinib (150 mg) | Gefitinib (250 mg) | NA | NA | NA | 13.08 | 26.64 | 26 ^c^ | 10 2 ^c^ | 67 | 0.55 | 0.92 | 1.00 | 1.00 | 0.00, 1.00 |
| Urata (2016) [35] e.pos_g.neg | Erlotinib (150 mg) | Gefitinib (250 mg) | NA | NA | NA | 31.97 | 13.44 | 99 ^c^ | 24 ^c^ | 67 | 0.55 | 0.92 | 1.00 | 1.00 | 1.00, 0.00 |
| Zhou (2014) [75] | Pemetrexed (500 mg/m^2^) | Gefitinib (250 mg) | 0.72 | -0.33 | 0.19 | 12.4 | 9.6 | 76 | 81 | 57 | 0.83 | 0.91 | 1.00 | 1.00 | 0.00 |

ECOG = Eastern Cooperative Oncology Group; EGFR = epidermal growth factor receptor; OS = overall survival, HR = hazard ratio; SE = standard error; NA = not available.

^a^ A hierarchical model was used so that nonsquamous information was only required for pemetrexed studies. Thus, the arm-based value for pemetrexed for the proportion of nonsquamous was used. All other studies have the mean value of each covariate for the study.

^b^ A hierarchical model was used so that EGFR status was only required for erlotinib and gefitinib studies. Thus, the arm-based value (if available) for erlotinib and gefitinib was used. All other studies have the mean value of each covariate for the study.

^c^ Sample sizes divided by 2 to avoid double counting. Urata (2016) [35] was treated as 4 separate studies due to the difficulty of modelling the efficacy of two TKIs in the same study which could both vary by EGFR mutation status.

## Proportional Hazards

Nonproportional hazard tests were conducted on reconstructed survival data. For OS, 2 out of the 32 studies or subgroups had significant (*P* < 0.05) nonproportional hazard ratios [39,70]. Shepherd et al. [70] (*P* = 0.002) showed a flattening off of the survival curve for best supportive care, and the study contained small samples (100 received best supportive care, 55 received docetaxel). It is possible that if the follow-up period had been longer the docetaxel arm also may have shown some flattening of the survival curve. The study by Borghaei et al. [39] (PD-L1 expression < 5%; *P* = 0.0065) showed a sudden drop in the survival probability for nivolumab at the start of the study, which the fractional polynomial model was not able to reproduce; the overall hazard ratio with docetaxel was 1.01. It is also worth noting that 2 out of 32 is close what we would expect to observe by chance.

For PFS, 7 out of the 29 studies or subgroups had significant nonproportional hazard ratios (Kawaguchi et al. [46]: *P* < 0.0001; Borghaei et al. [39]: < 5% PD-L1 expression: *P* < 0.0001, ≥ 5% PD-L1 expression: *P* = 0.0022; Brahmer et al. [40]: < 5% PD-L1 expression: *P* = 0. 0043; Kim et al. [64]: *P* = 0.0007; Garon et al. [54]: *P* = 0.0026; Zhou et al. [74]: *P* = 0.0060). The fractional polynomial models gave a good visual fit to these studies (see Appendix Figure S-2).

## Heterogeneity and Inconsistency

For OS, evidence of substantial heterogeneity existed in the network of evidence (Higgin’s I^2^ = 68.7%; test of heterogeneity: *P* < 0.0001). Pairwise meta-analysis of duplicate comparisons and the experimental node-splitting technique revealed significant heterogeneity for erlotinib and gefitinib by EGFR status (*P* < 0.0001; Higgin’s I^2^ = 80.8%), docetaxel versus pemetrexed by histology (*P* = 0.0238; Higgin’s I^2^ = 56.4%), and docetaxel versus nivolumab by PD-L1 expression and histology (*P* = 0.0024; Higgin’s I^2^ = 80.1%). Node-splitting revealed inconsistency for docetaxel versus erlotinib, which was likely to have been due to differences in EGFR mutation but could not be confirmed by this technique.

For PFS, evidence of considerable heterogeneity existed in the network of evidence (Higgin’s I^2^ = 86.3%; test of heterogeneity: *P* < 0.0001). Pairwise meta-analysis of duplicate comparisons and the experimental node-splitting technique revealed significant heterogeneity for erlotinib and gefitinib by EGFR status (*P* < 0.0001; Higgin’s I^2^ = 91.7%), gefitinib versus pemetrexed (*P* < 0.0001; Higgin’s I^2^ = 87.4%), and docetaxel versus nivolumab by PD-L1 expression (*P* < 0.0001; Higgin’s I^2^ = 87.7%). Node-splitting revealed inconsistency for docetaxel versus erlotinib and pemetrexed versus erlotinib, which was likely to have been due to differences in EGFR mutation but could not be confirmed by this technique.

## Model Fit Statistics

For models that did not take account of the treatment covariate interactions, i.e., that did not include hierarchical exchangeable structures, the random-effects model gave a lower deviance information criterion (DIC) scores than the fixed-effects models for both OS (Table S-3) and PFS (Table S-4). When hierarchical exchangeable structures were included, there was a large drop in DIC score and the fixed-effects models fitted slightly better than the random-effects models. Without the hierarchical exchangeable structure, the heterogeneity parameters for OS and PFS were bounded well away from zero. However, for the models that included hierarchical exchangeable structures, the heterogeneity parameters were not bounded away from zero and were considerably reduced. The addition of covariates (time since publication, age, proportion of patients with ECOG ≥ 1, proportion with stage IV disease, and proportion of Asian patients) did not result in a meaningful improvement in DIC score or reduce the value of the heterogeneity parameter. Performance status was not consistently reported. Where reported, the proportion of patients with ECOG ≥ 1 is presented in Appendix Table S-2. Studies also varied in inclusion criteria for brain metastases. Some studies provide detailed information. Other studies gave no information. This information, on brain metastases, was not collected for the literature review. Only LUME-Lung 1, provided subgroup data for the presence and absence of brain metastases.

All the models tested appeared to have converged properly (iteration plots and distribution charts) and without correlations between Bayesian draws (Gelman-Rubin diagnostics).

Table S3. Model Fit Statistics for OS

| Model | DIC | σ (Credible Intervals) |
| --- | --- | --- |
| Fixed effects, no hierarchical exchangeable structure | 37.5 |  |
| Random effects, no hierarchical exchangeable structure | 14.3 | 0.292 (0.174, 0.447) |
| Fixed effects, with hierarchical exchangeable structures | –4.4 |  |
| Random effects, with hierarchical exchangeable structures | –2.3 | 0.078 (0.003, 0.198) |

Table S4. Model Fit Statistics for PFS: Second-Order Fractional Polynomial NMAs

| Model | DIC | Parameter | σ (credible intervals) |
| --- | --- | --- | --- |
| Fixed effects, no hierarchical exchangeable structure | 5823.5 | — | — |
| Random scale, fixed shape, no hierarchical structure | 5810.1 | Scale | 0.277 (0.111, 0.519) |
| Fixed effects, with hierarchical exchangeable structure | 4208.6 | — | — |
| Random scale, fixed shape with hierarchical exchangeable structures | 4209.0 | Scale | 0.099 (0.003, 0.295) |
| Random scale, random shape parameters with hierarchical exchangeable structures | 4214.9 | Scale | 0.101 (0.039, 0.246) |
|  |  | 1st shape | 0.136 (0.041, 0.396) |
|  |  | 2nd shape | 0.153 (0.039, 0.499) |

Table S5. Summary of Interventions That Showed a Significant (P < 0.05) Benefit Over Single-agent Docetaxel (75 mg/m^2^): Random-Effects NMA

| Histology | PD-L1 Expression | EGFR Mutation | Occurrence (Non-Asian) | Occurrence (Asian) | OS (Hazard Ratio NMA) | PFS (Fractional Polynomial NMA)^a^ |
| --- | --- | --- | --- | --- | --- | --- |
| Nonsquamous | < 5% | Negative | 32.8% | 21.2% | — | — |
| Squamous | < 5% | Negative | 21.0% | 20.2% | Nivolumab: 5.7 (0.6, 13.1) | Nivolumab: 2.7 (0.1, 6.2)^b^ |
| Nonsquamous | ≥ 5% | Negative | 20.5% | 13.3% | Nivolumab: 12.5 (4.8, 23.9) | Nivolumab: 4.4 (0.8, 7.6) |
| Squamous | ≥ 5% | Negative | 13.2% | 12.7% | Nivolumab: 7.9 (1.4, 18.1) | Nivolumab: 5.4 (1.6, 9.6) |
| Nonsquamous | < 5% | Positive | 7.2% | 18.8% | Docetaxel + erlotinib: 13.4 (3.8, 28.8)  Erlotinib + pemetrexed: 8.3 (0.0, 29.1)^b^  Erlotinib: 7.1 (1.7, 15.4)  Gefitinib: 4.4 (0.4, 11.1) | Gefitinib: 7.2 (2.8, 14.0)  Docetaxel + erlotinib: 6.1 (2.6, 9.5)  Erlotinib: 3.4 (0.7, 7.2) |
| Squamous | < 5% | Positive | 0.5% | 1.3% | Docetaxel + erlotinib: 11.8 (3.4, 25.6)  Erlotinib: 6.3 (1.5, 13.5)  Nivolumab: 5.7 (0.6, 13.1)  Gefitinib: 3.9 (0.4, 9.8) | Gefitinib: 7.2 (2.8, 14.0)  Docetaxel + erlotinib: 6.1 (2.6, 9.5)  Erlotinib: 3.4 (0.7, 7.2)  Nivolumab: 2.7 (0.1, 6.2) ^b^ |
| Nonsquamous | ≥ 5% | Positive | 4.5% | 11.8% | Docetaxel + erlotinib: 13.4 (3.8, 28.8)  Nivolumab: 12.5 (4.8, 23.9)  Erlotinib + pemetrexed: 8.3 (0.0, 29.1) ^b^  Erlotinib: 7.1 (1.7, 15.4)  Gefitinib: 4.4 (0.4, 11.1) | Gefitinib: 7.2 (2.8, 14.0)  Docetaxel + erlotinib: 6.1 (2.6, 9.5)  Nivolumab: 4.4 (0.8, 7.6)  Erlotinib: 3.4 (0.7, 7.2) |
| Squamous | ≥ 5% | Positive | 0.3% | 0.8% | Docetaxel + erlotinib: 11.8 (3.4, 25.6)  Nivolumab: 7.9 (1.4, 18.1)  Erlotinib: 6.3 (1.5, 13.5)  Gefitinib: 3.9 (0.4, 9.8) | Gefitinib: 7.2 (2.8, 14.0)  Docetaxel + erlotinib: 6.1 (2.6, 9.5)  Nivolumab: 5.4 (1.6, 9.6)  Erlotinib: 3.4 (0.7, 7.2) |

^a^ PFS fractional polynomial model included a heterogeneity parameter for only random scale effect; shape parameters were treated as fixed effects.

^b^ Credible intervals sufficiently close to zero to not always be bounded away from zero for repeat runs of the models.

Docetaxel = docetaxel (75 mg/m^2^) 3 times a week; difference in mean survival relative to docetaxel (75 mg/m^2^) after colon with 95% credible intervals in parentheses. Occurrence of each tumor subgroup are only approximate and based on the following: 65% nonsquamous, 35% squamous [5]; non-Asian: 18% EGFR mutation positive in nonsquamous tumors; Asian: 47% EGFR mutation in nonsquamous tumors [75]; 8 times more likely to be EGFR positive if nonsquamous compared to squamous [76] and 38.5% PD-L1 ≥ 5% (combined data from Borghaei et al. [39]; Brahmer et al. [40]). Predictions from the NMA assumed relationships for each factor are the same across any other factor. This allowed predictions to be made across all subgroups, but where subgroups are rare there may be little actual direct evidence for that patient population.

Table S6. Available Comparable Data for PD-L1 Inhibitors

| Treatment | n1 | n2 | Histology | PD-L1 | OS (HR, CI) | PFS (HR, CI) |
| --- | --- | --- | --- | --- | --- | --- |
| Nivolumab (3mg/kg) | 54 | 52 | Sq | < 1% | 0.58 (0.37, 0.92) | 0.66 (0.43, 1.00) |
|  | 63 | 56 |  | ≥ 1% | 0.69 (0.45, 1.05) | 0.67 (0.44, 1.01) |
|  | 42 | 39 |  | < 5% | 0.70 (0.47, 1.02) | 0.75 (0.52, 1.08) |
|  | 42 | 39 |  | ≥ 5% | 0.53 (0.31, 0.89) | 0.54 (0.32, 0.90) |
|  | 108 | 101 | Non-sq | < 1% | 0.90 (0.66, 1.24) | 1.19 (0.88, 1.61) |
|  | 123 | 123 |  | ≥ 1% | 0.59 (0.43, 0.82) | 0.70 (0.53, 0.94) |
|  | 136 | 138 |  | < 5% | 1.01 (0.77, 1.34) | 1.31 (1.01, 1.71) |
|  | 95 | 86 |  | ≥ 5% | 0.43 (0.30, 0.63) | 0.54 (0.39, 0.76) |
| Pembrolizumab  (2 mg/kg) | 339 | 309 | ITT (76% non-sq) | ≥ 1% | 0.71 (0.58, 0.88) | 0.88 (0.74, 1.05) |
| Pembrolizumab (10 mg/kg) | 343 |  |  |  | 0.61 (0.49, 0.75) | 0.79 (0.66, 0.94) |
| Atezolizumab (1200 mg) | 51 | 41 | ITT (66% non-sq) | < 1% | 1.04 (0.62, 1.75) | 1.12 (0.72, 1.77) |
|  | 93 | 102 |  | ≥ 1% | 0.59 (0.40, 0.85) | 0.85 (0.63, 1.16) |
|  | 50 | 55 |  | ≥ 5% | 0.54 (0.33, 0.89) | 0.72 (0.47, 1.10) |

CI = 95% confidence intervals; HR = hazard ratio; ITT = intent-to-treat; n_1_ = sample size for comparator; n_2_ = sample size for docetaxel (75 mg/m^2^); non-sq = nonsquamous; OS = overall survival; PD-L1 = programmed death ligand 1; PFS = progression-free survival; sq = squamous.

Note: There may be differences in the methods used to assess PD-L1 expression between studies. The nivolumab studies assessed PD-L1 with the use of a validated automated immunohistochemical assay (Dako North America) that used a rabbit monoclonal antihuman PD-L1 antibody (clone 28–8, Epitomics) [39,40]. The atezolizumab study assessed PD-L1 expression with the VENTANA SP142 PD-L1 immunohistochemistry assay (Ventana Medical Systems, Tucson, AZ, USA) [48]. The pembrolizumab study assessed PD-L1 with an immunohistochemistry assay (Dako; Carpinteria, CA, USA) with the murine 22C3 anti-human PD-L1 antibody [18].

## Bayesian Fractional Polynomial Model Fitted to the Reference Treatment for Overall Survival

Fractional polynomial models with random scale and shape effects for study were fitted to the reference data (docetaxel 75 mg/m^2^). Second-order models would not converge but suggested that the second shape parameter was close to zero and was therefore redundant. The first-order model that gave the best fit in terms of DIC score was a model with a power function of –2 (DIC = 2190.3). The model was improved further with the addition of covariates for time since publication and proportion of nonsquamous patients (DIC = 2183.4). The hazard rates were predicted for time since publication equal to zero and for 0% and 100% nonsquamous populations. The resulting survival probability curves are shown in Figure S-1 and are similar to those presented by Penrod et al. [47].

Figure S1. First-Order Fractional Polynomial Model With Random Scale and Shape Effects Fitted to the Reference Treatment (Docetaxel 75mg/m^2^) by Histology


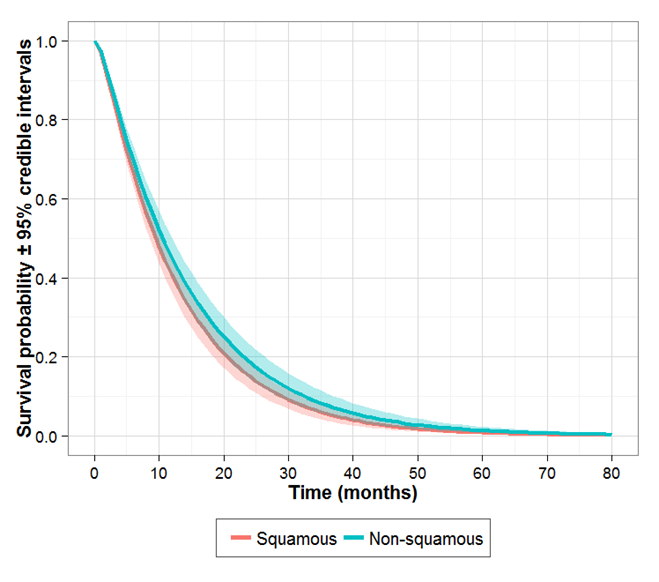


## Bayesian Fractional Polynomial Model Fitted to the Network of Evidence for Progression-Free Survival

Figure S2. Predicted Progression-Free Survival by Study Arm


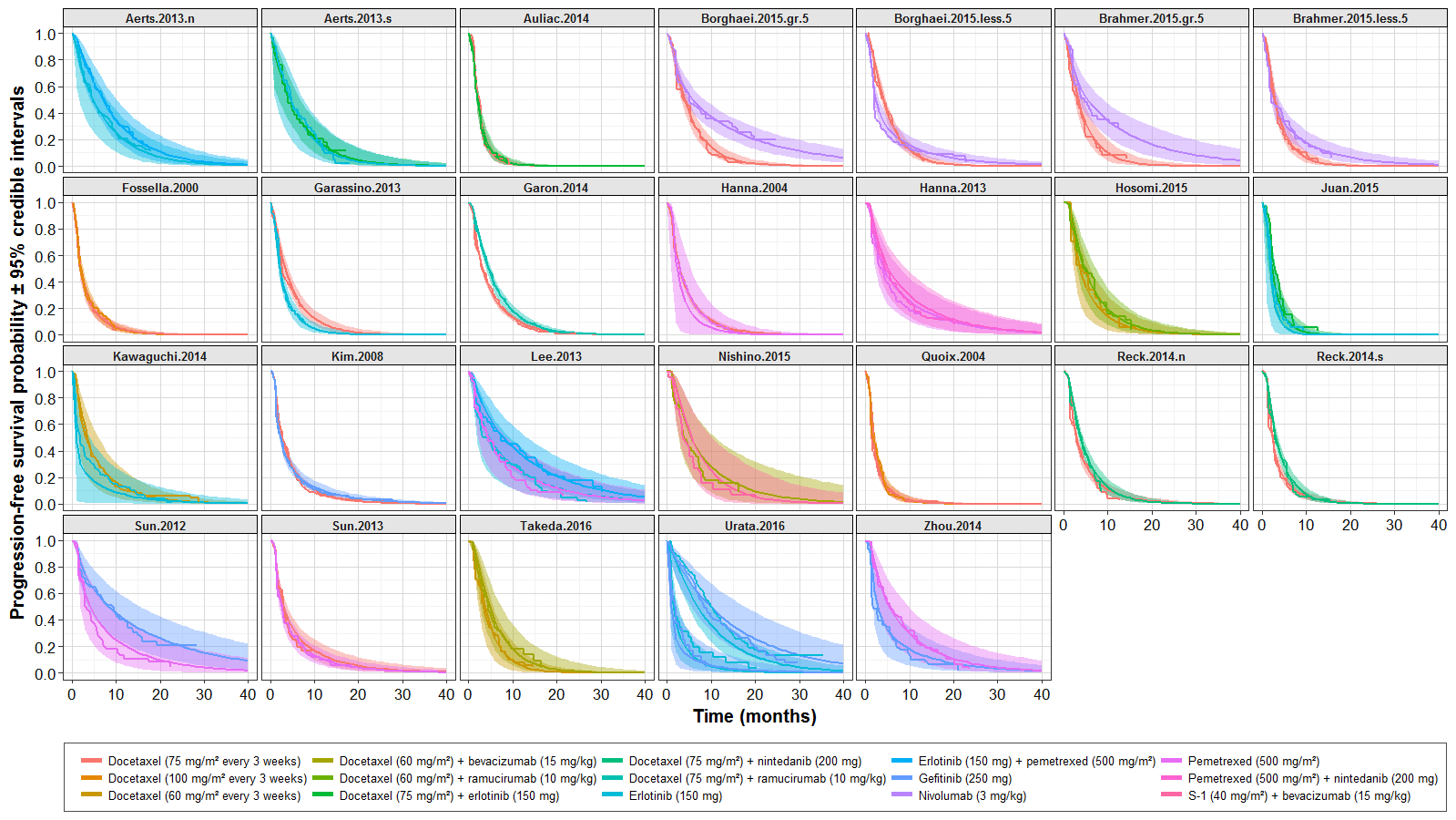


Note: Stepped line represents the Kaplan-Meier estimates for each trial arm. Smooth lines represent the predictions from the NMA and shaded areas represent 95% credible intervals from the NMA.

## Results for Nonsquamous, PD-L1 Expression < 5%, EGFR Mutation Negative


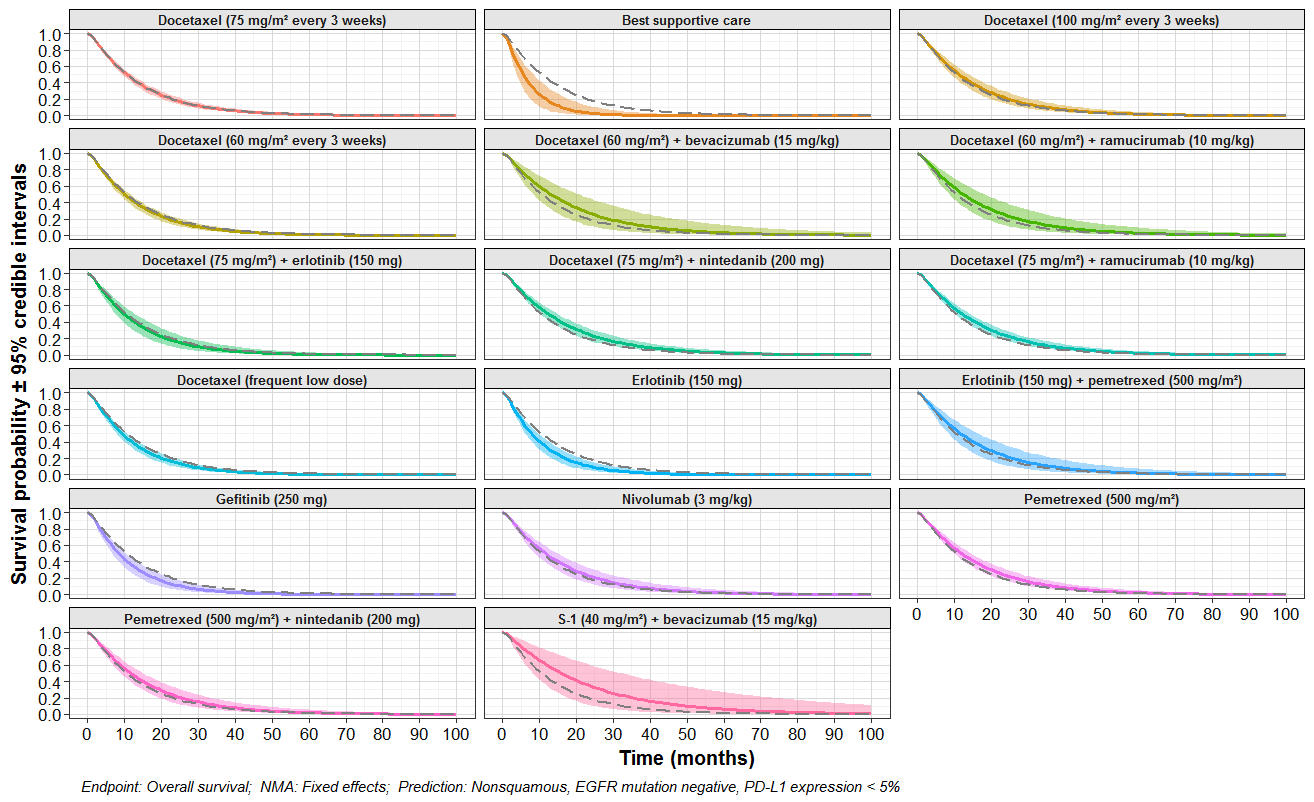
Figure S3. Probability of Overall Survival Curves: Nonsquamous, PD-L1 Expression < 5%, EGFR Mutation Negative

Note: Dotted line represents docetaxel (75 mg/m^2^ every 3 weeks).


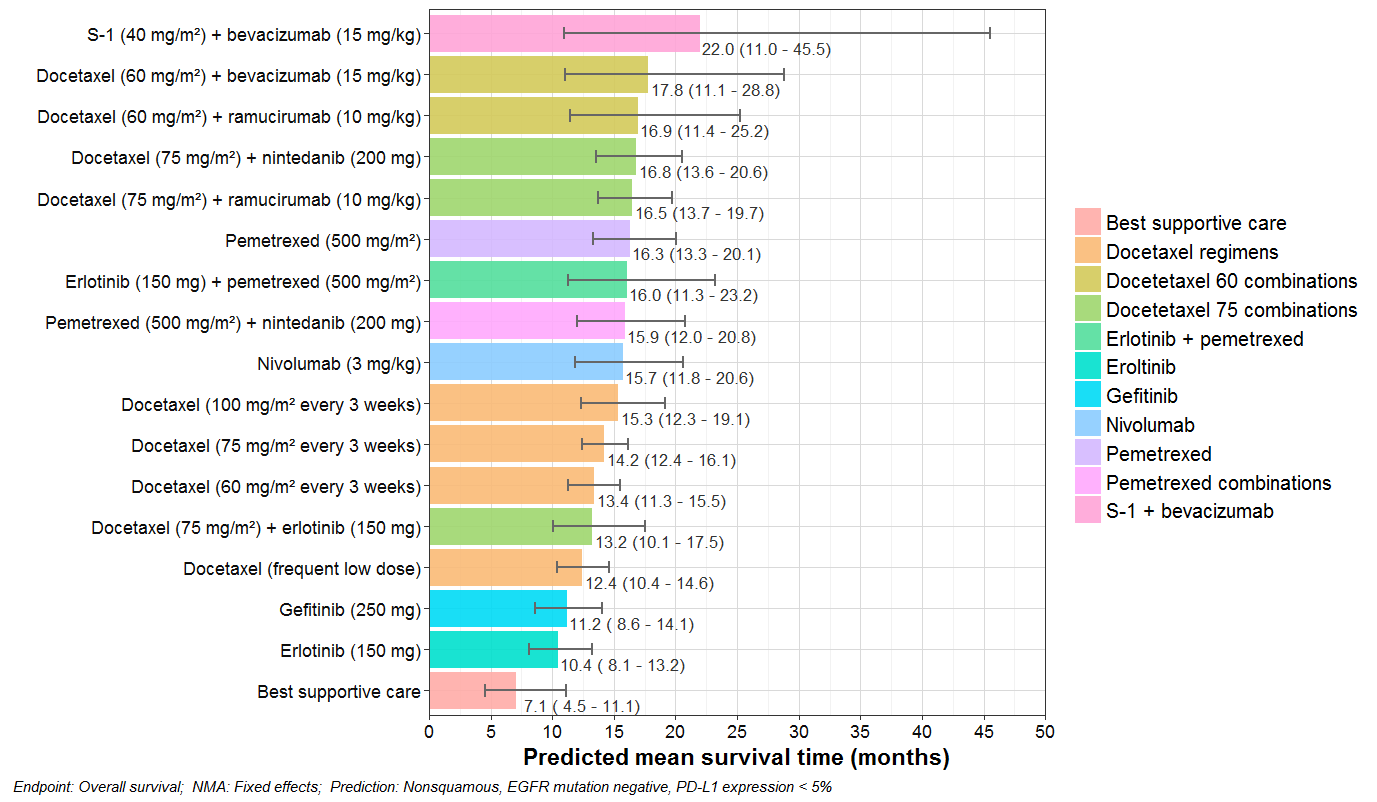
Figure S4. Predicted Mean Overall Survival Time (Months): Nonsquamous, PD-L1 Expression < 5%, EGFR Mutation Negative

Note: All pairwise comparisons of mean overall survival times (months) for nonsquamous, EGFR mutation negative, PD-L1 expression < 5% in main body of manuscript.


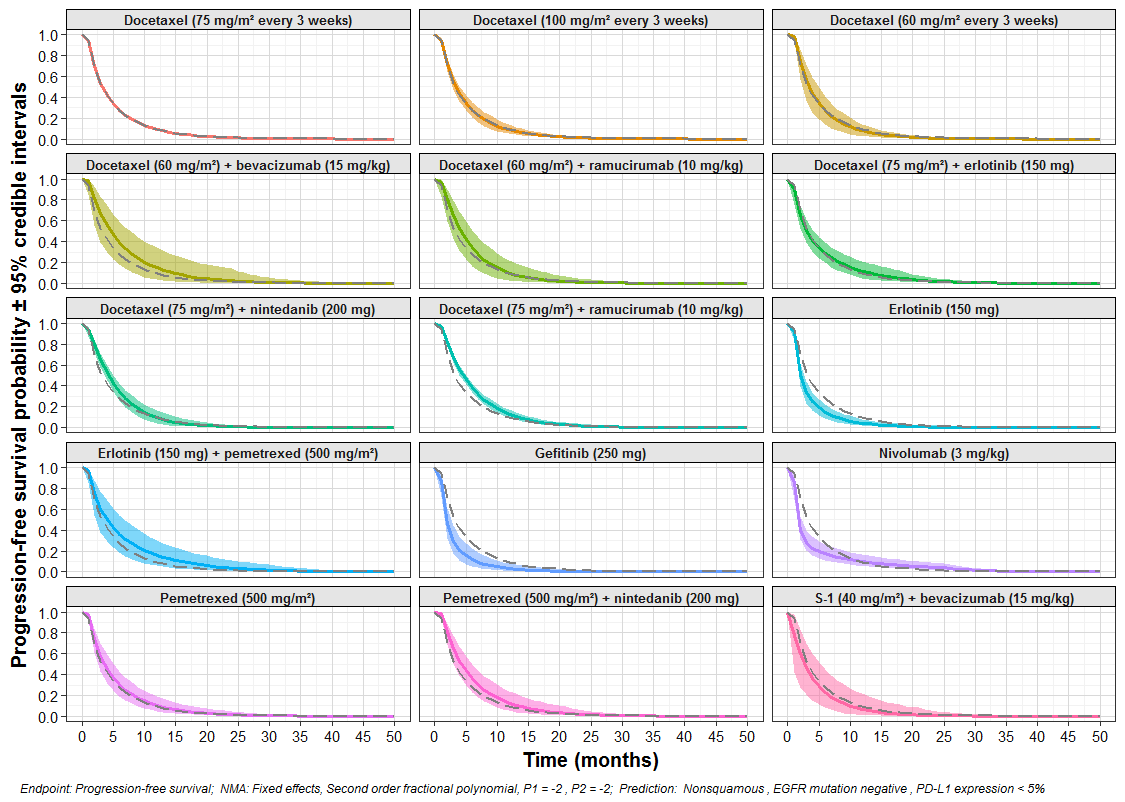
Figure S5. Probability of Progression-Free Survival Curves: Nonsquamous, PD-L1 Expression < 5%, EGFR Mutation Negative

Note: Dotted line represents docetaxel (75 mg/m^2^ every 3 weeks).


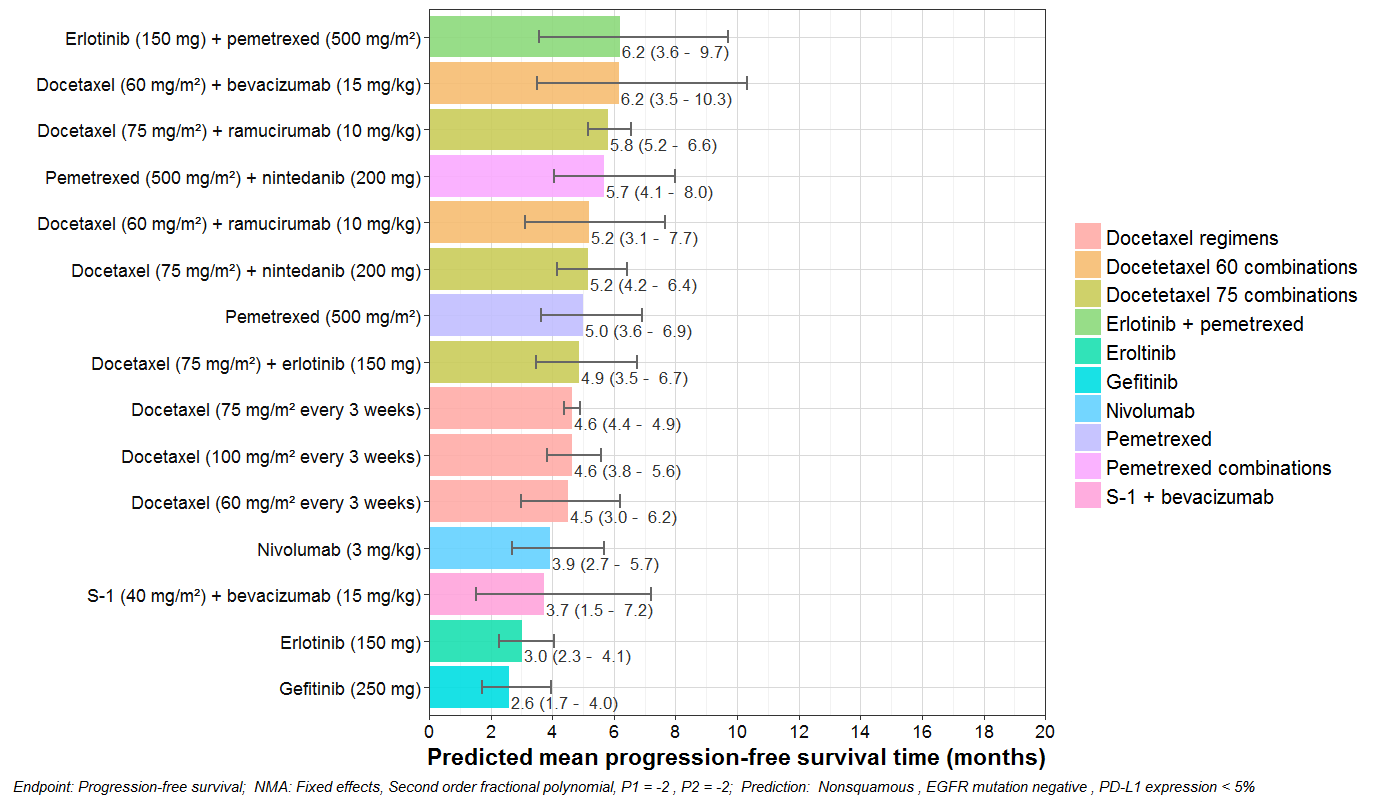
Figure S6. Predicted Mean Progression-Free Survival Time (Months): Nonsquamous, PD-L1 Expression < 5%, EGFR Mutation Negative

Note: All pairwise comparisons of mean progression-free survival times (months) for nonsquamous, EGFR mutation negative, PD-L1 <5% expression in main body of manuscript.

## Results for Squamous, PD-L1 Expression < 5%, EGFR Mutation Negative

Figure S7. Probability of Overall Survival Curves: Squamous, PD-L1 Expression < 5%, EGFR Mutation Negative


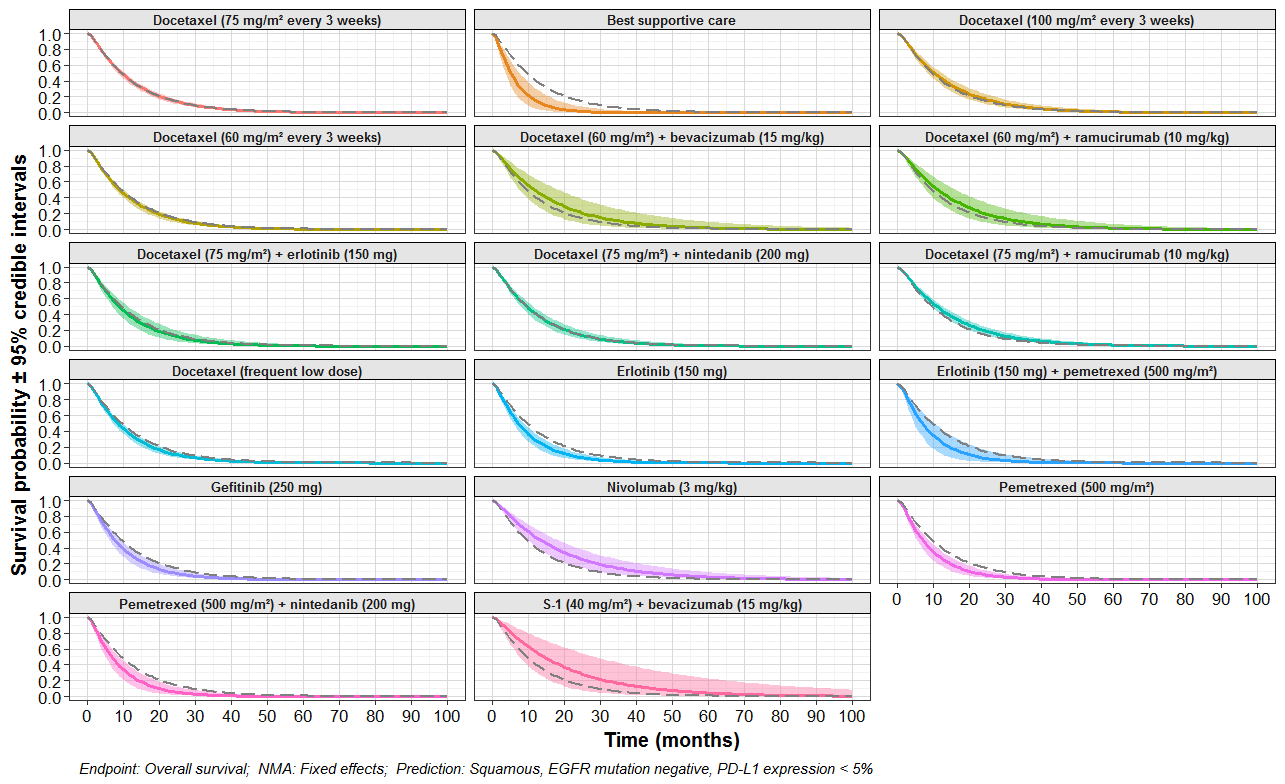


Note: Dotted line represents docetaxel (75 mg/m^2^ every 3 weeks).

Figure S8. Predicted Mean Overall Survival Time (Months): Squamous, PD-L1 Expression < 5%, EGFR Mutation Negative


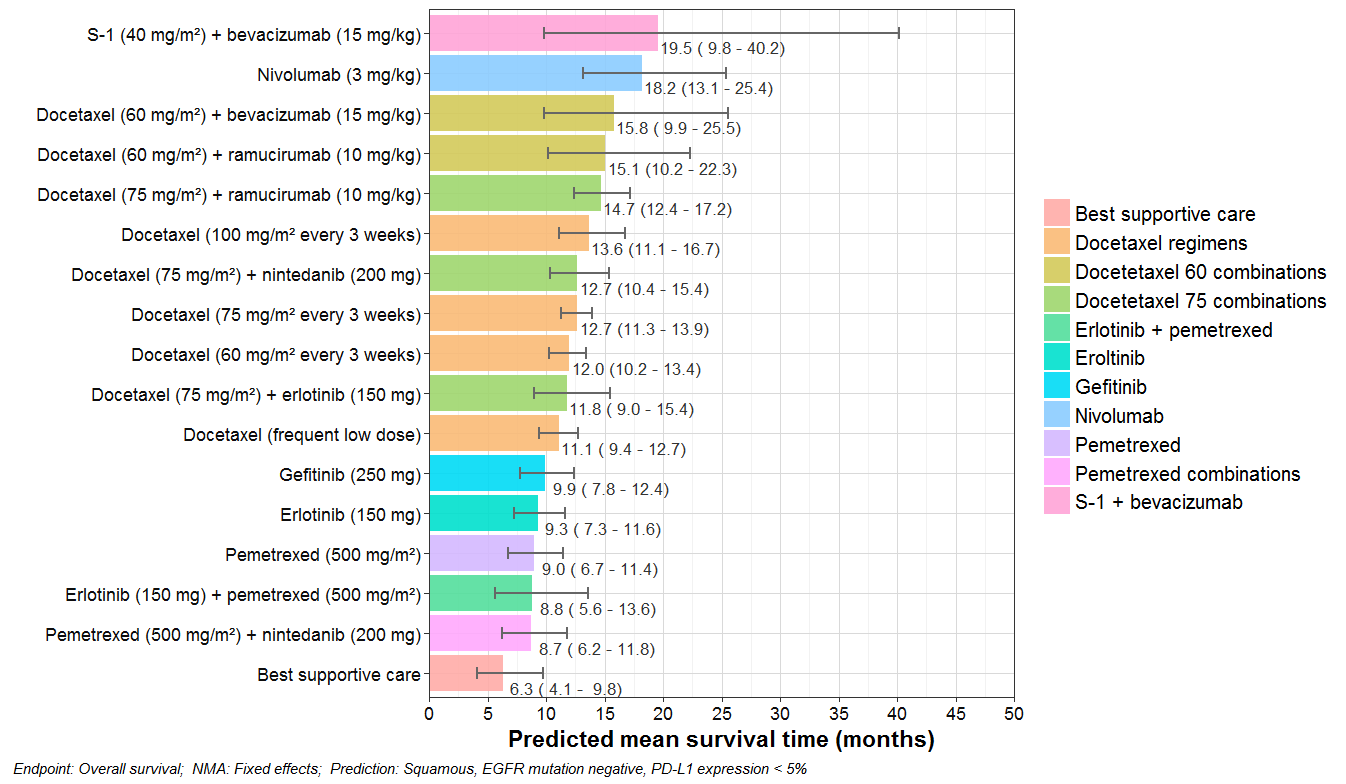

Figure S9. All Pairwise Comparisons of Mean Overall Survival Times (Months): Squamous, PD-L1 Expression < 5%, EGFR Mutation Negative


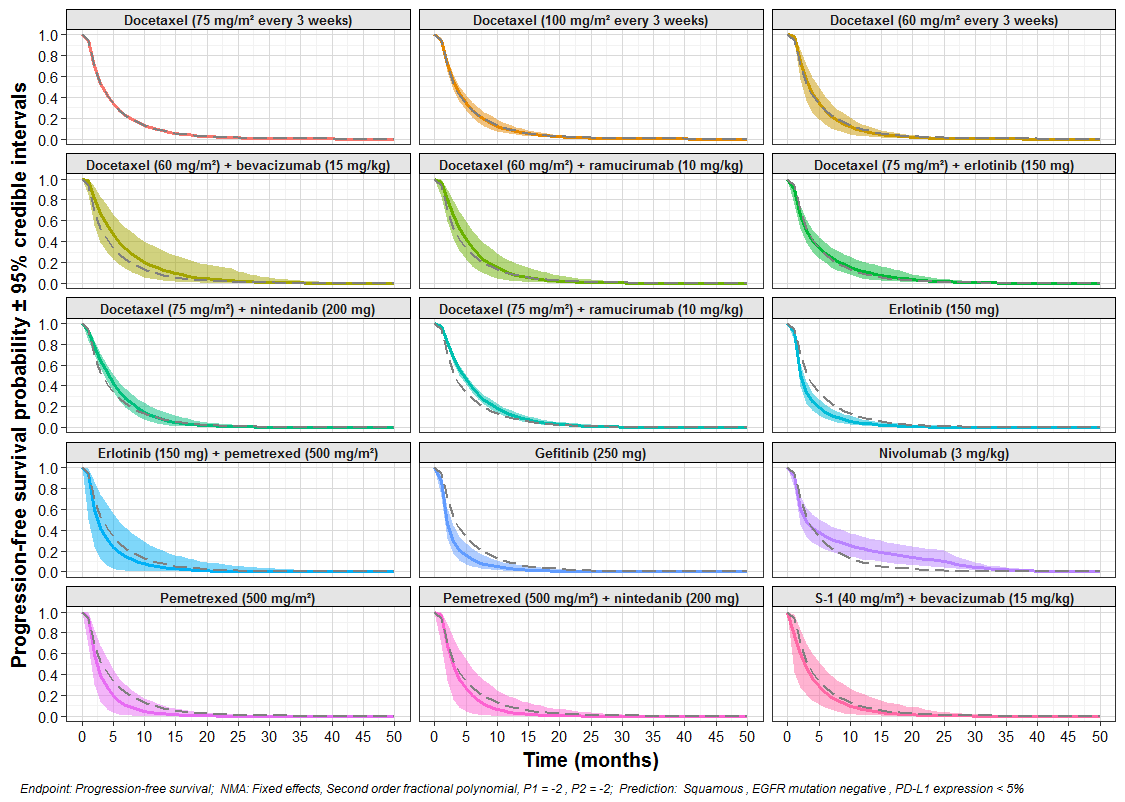
Figure S10. Probability of Progression-Free Survival Curves: Squamous, PD-L1 Expression < 5%, EGFR Mutation Negative

Note: Dotted line represents docetaxel (75 mg/m^2^ every 3 weeks).


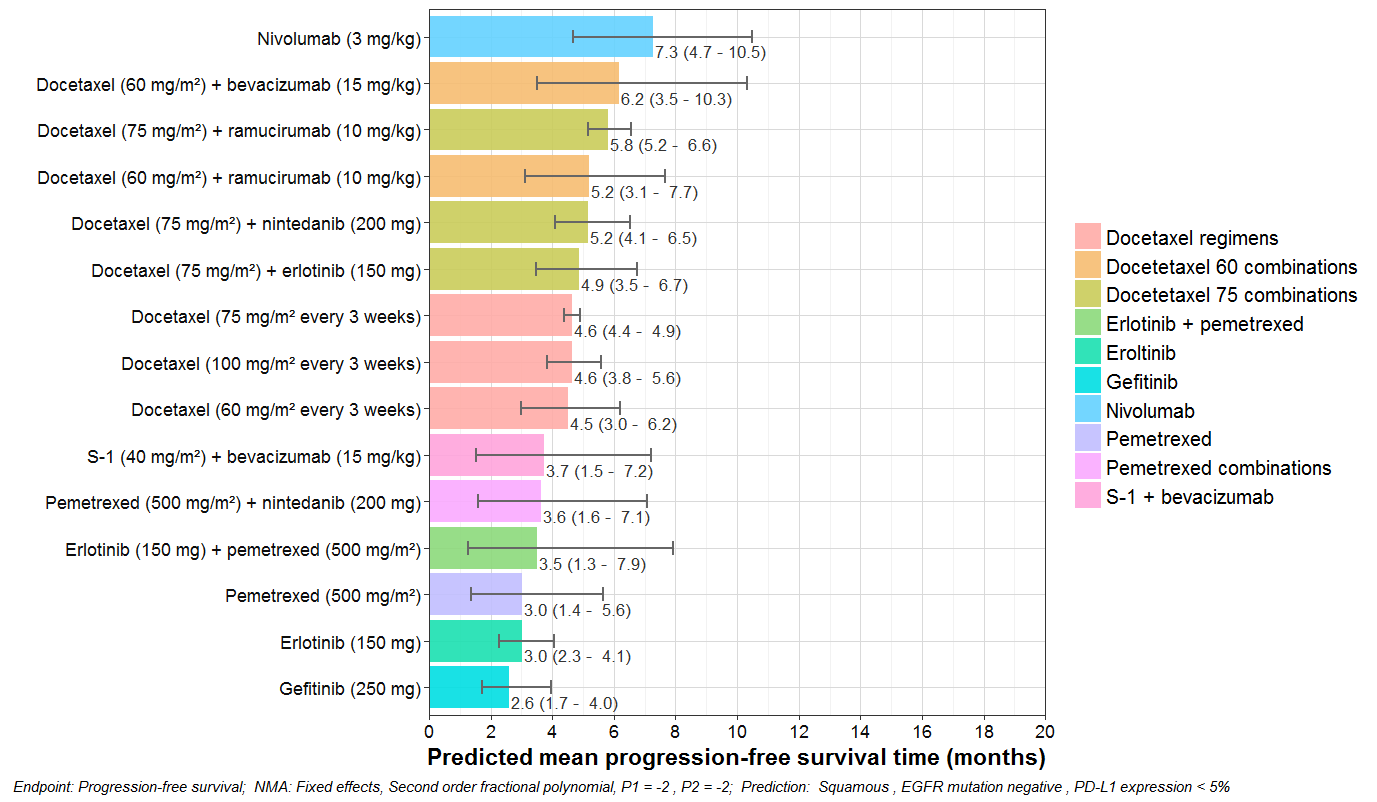
Figure S11. Predicted Mean Progression-Free Survival Time (Months): Squamous, PD-L1 Expression < 5%, EGFR Mutation Negative

Figure S12. All Pairwise Comparisons of Mean Progression-Free Survival Times (Months): Squamous, PD-L1 Expression < 5%, EGFR Mutation Negative

## Results for Nonsquamous, PD-L1 Expression ≥ 5%, EGFR Mutation Negative


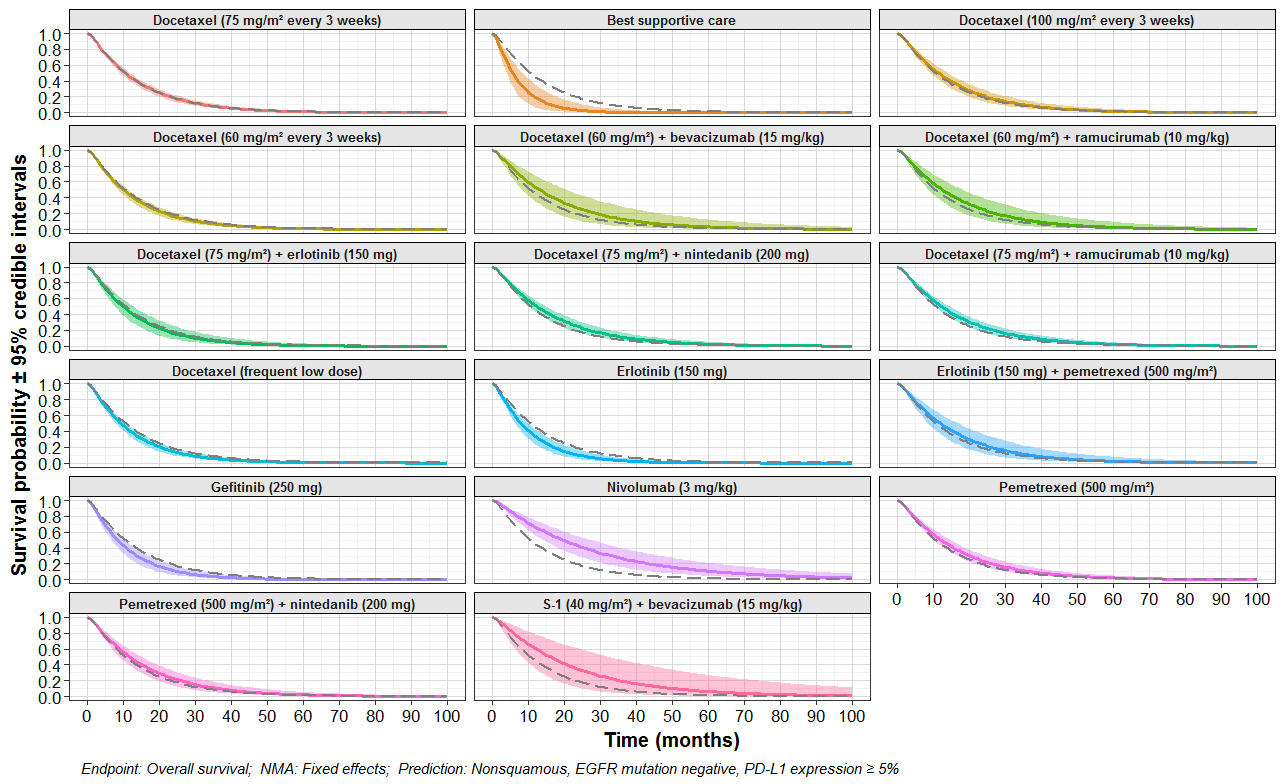
Figure S13. Probability of Overall Survival Curves: Nonsquamous, PD-L1 Expression ≥ 5%, EGFR Mutation Negative

Note: Dotted line represents docetaxel (75 mg/m^2^ every 3 weeks).

Figure S14. Predicted Mean Overall Survival Time (Months): Nonsquamous, PD-L1 Expression ≥ 5%, EGFR Mutation Negative


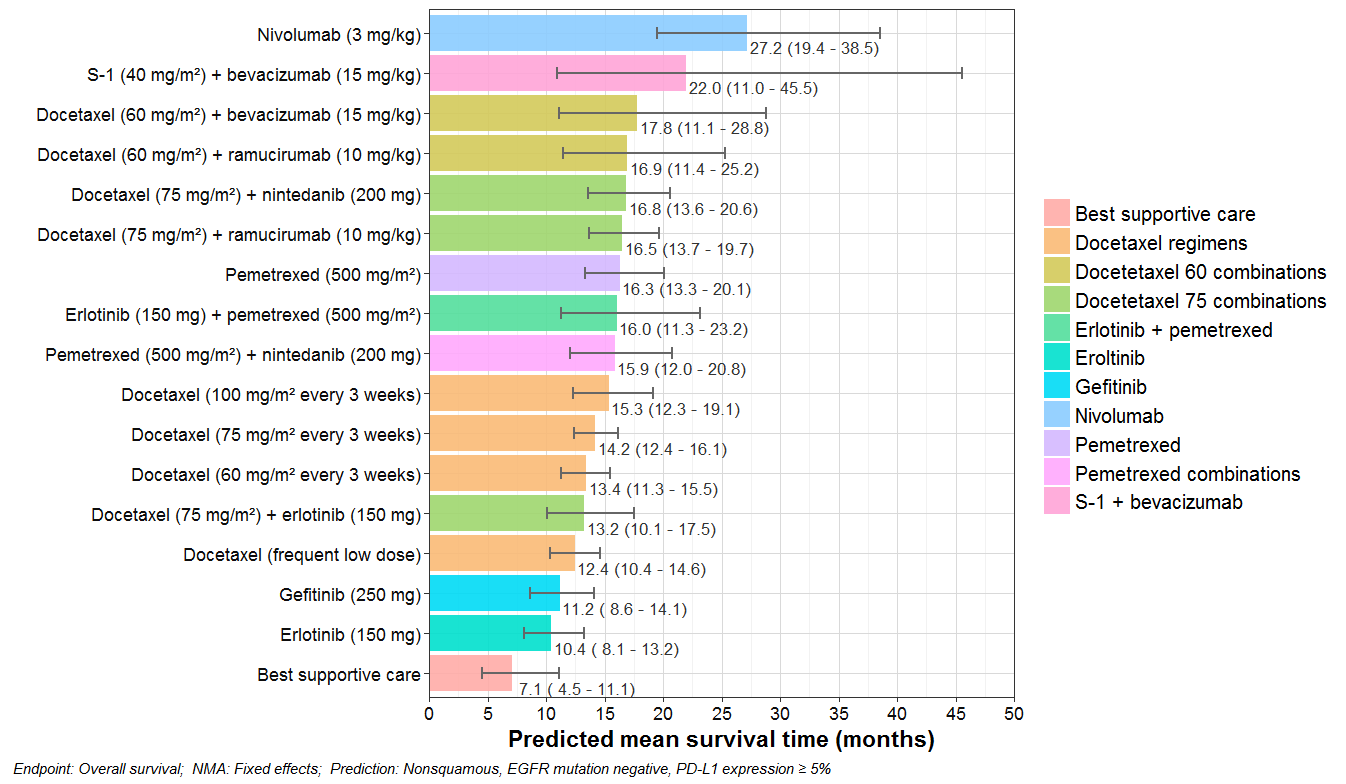

Figure S15. All Pairwise Comparisons of Mean Overall Survival Times (Months): Nonsquamous, PD-L1 Expression ≥ 5%, EGFR Mutation Negative


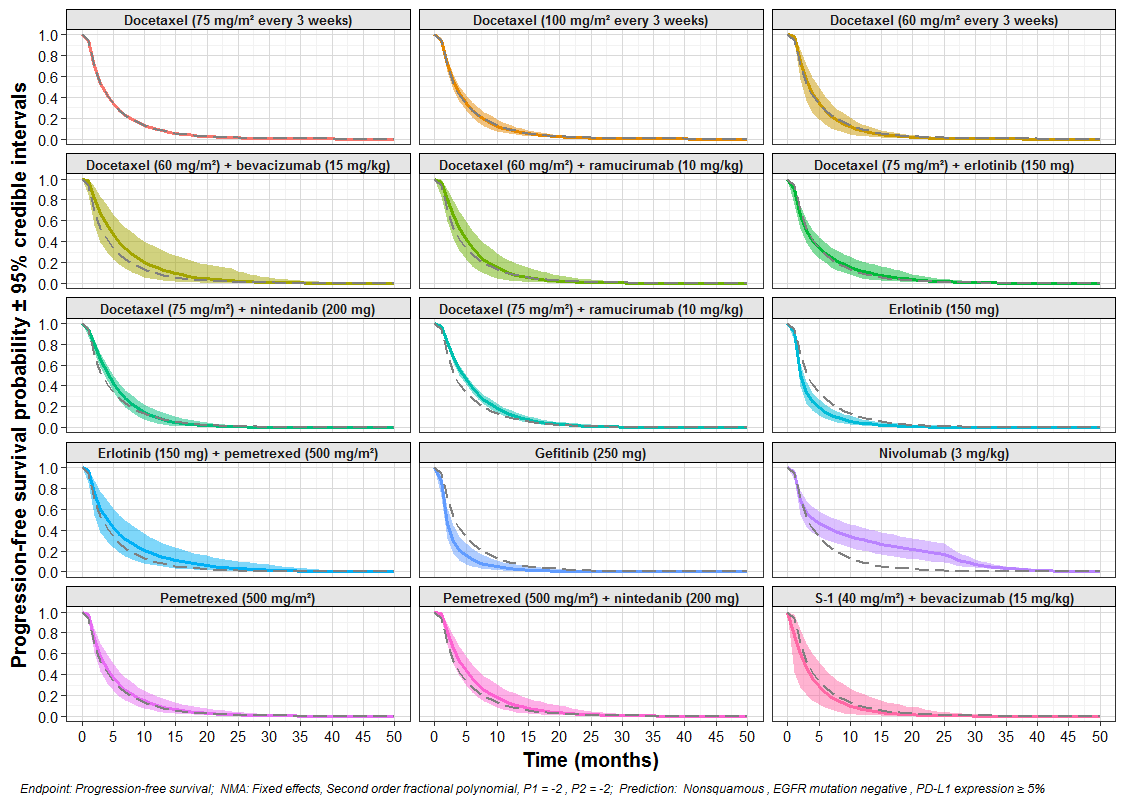
Figure S16. Probability of Progression-Free Survival Curves: Nonsquamous, PD-L1 Expression ≥ 5%, EGFR Mutation Negative

Note: Dotted line represents docetaxel (75 mg/m^2^ every 3 weeks).

Figure S17. Predicted Mean Progression-Free Survival Time (Months): Nonsquamous, PD-L1 Expression ≥ 5%, EGFR Mutation Negative


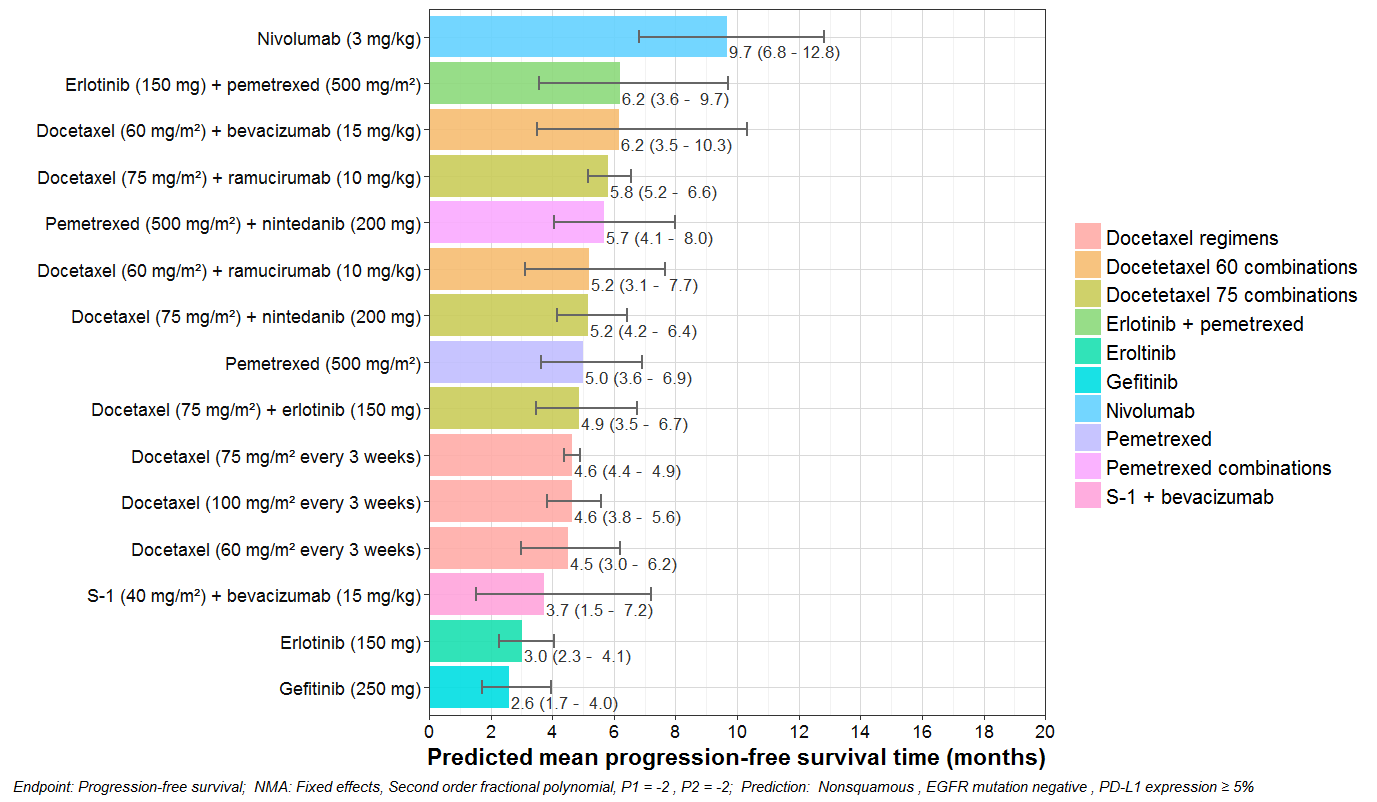

Figure S18. All Pairwise Comparisons of Mean Progression-Free Survival Times (Months): Nonsquamous, PD-L1 Expression ≥ 5%, EGFR Mutation Negative

## Results for Squamous, PD-L1 Expression ≥ 5%, EGFR Mutation Negative

Figure S19. Probability of Overall Survival Curves: Squamous, PD-L1 Expression ≥ 5%, EGFR Mutation Negative


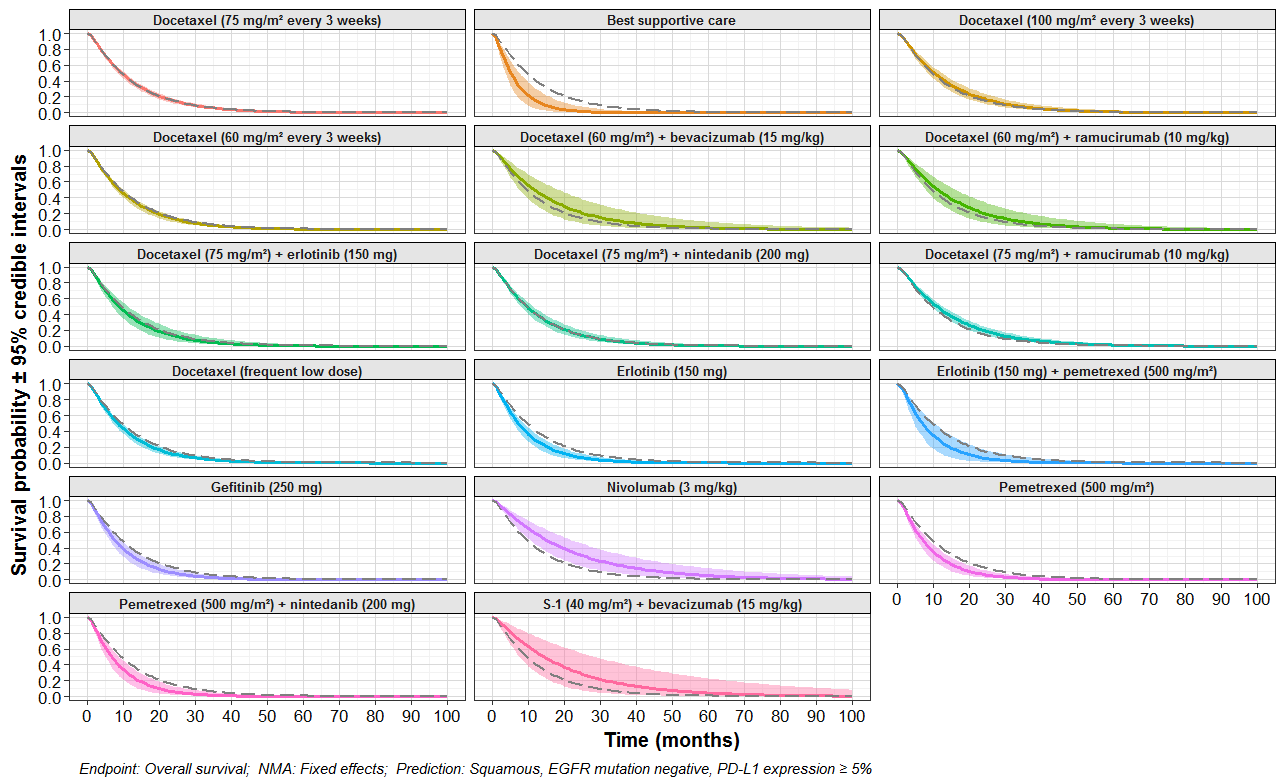


Note: Dotted line represents docetaxel (75 mg/m^2^ every 3 weeks).

Figure S20. Predicted Mean Overall Survival Time (Months): Squamous, PD-L1 Expression ≥ 5%, EGFR Mutation Negative


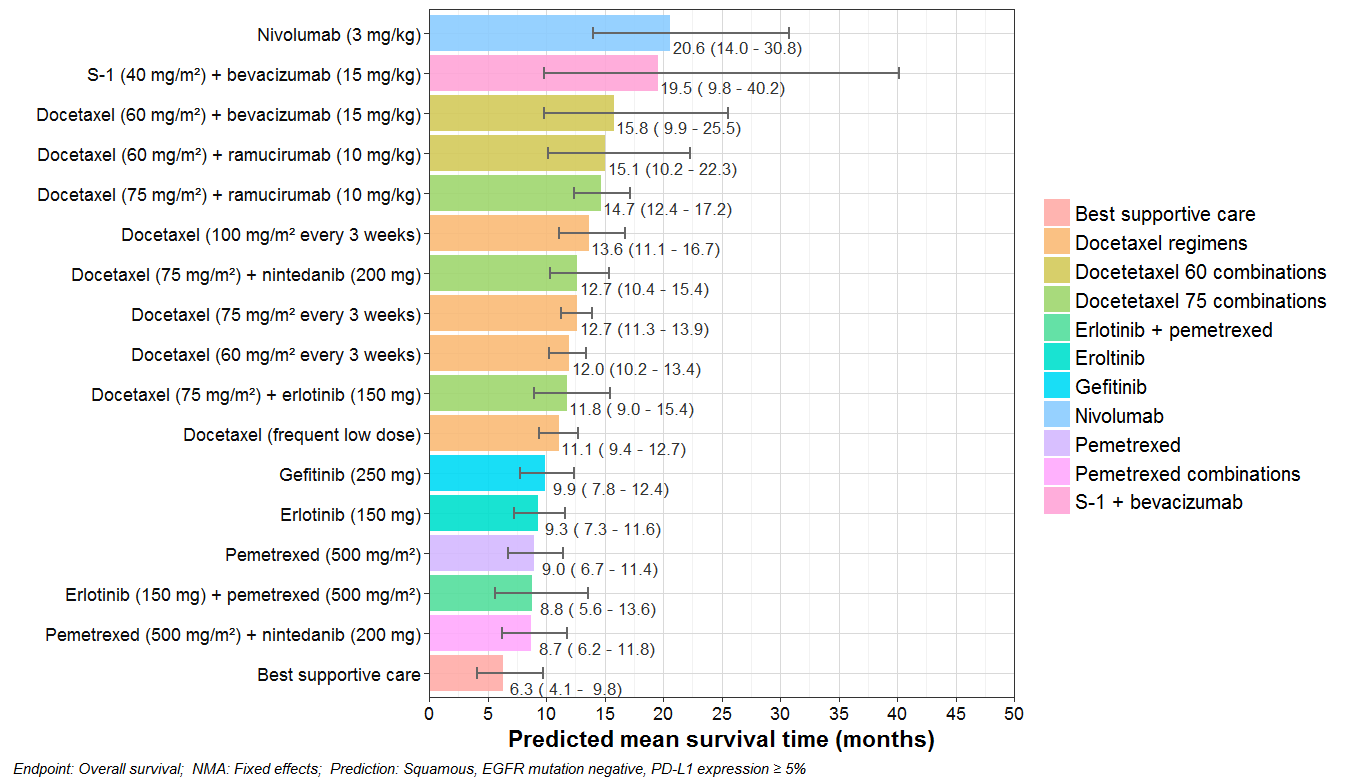

Figure S21. All Pairwise Comparisons of Mean Overall Survival Times (Months): Squamous, PD-L1 Expression ≥ 5%, EGFR Mutation Negative


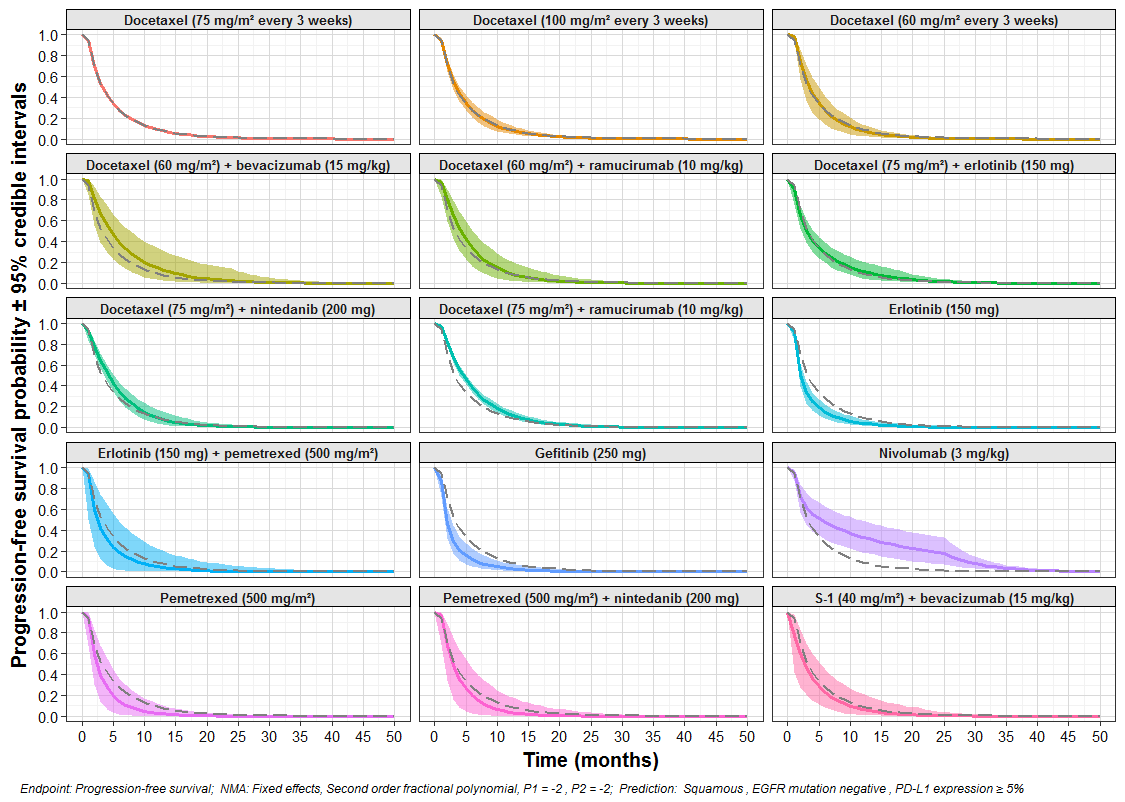
Figure S22. Probability of Progression-Free Survival Curves: Squamous, PD-L1 Expression ≥ 5%, EGFR Mutation Negative

Note: Dotted line represents docetaxel (75 mg/m^2^ every 3 weeks).

Figure S23. Predicted Mean Progression-Free Survival Time (Months): Squamous, PD-L1 Expression ≥ 5%, EGFR Mutation Negative


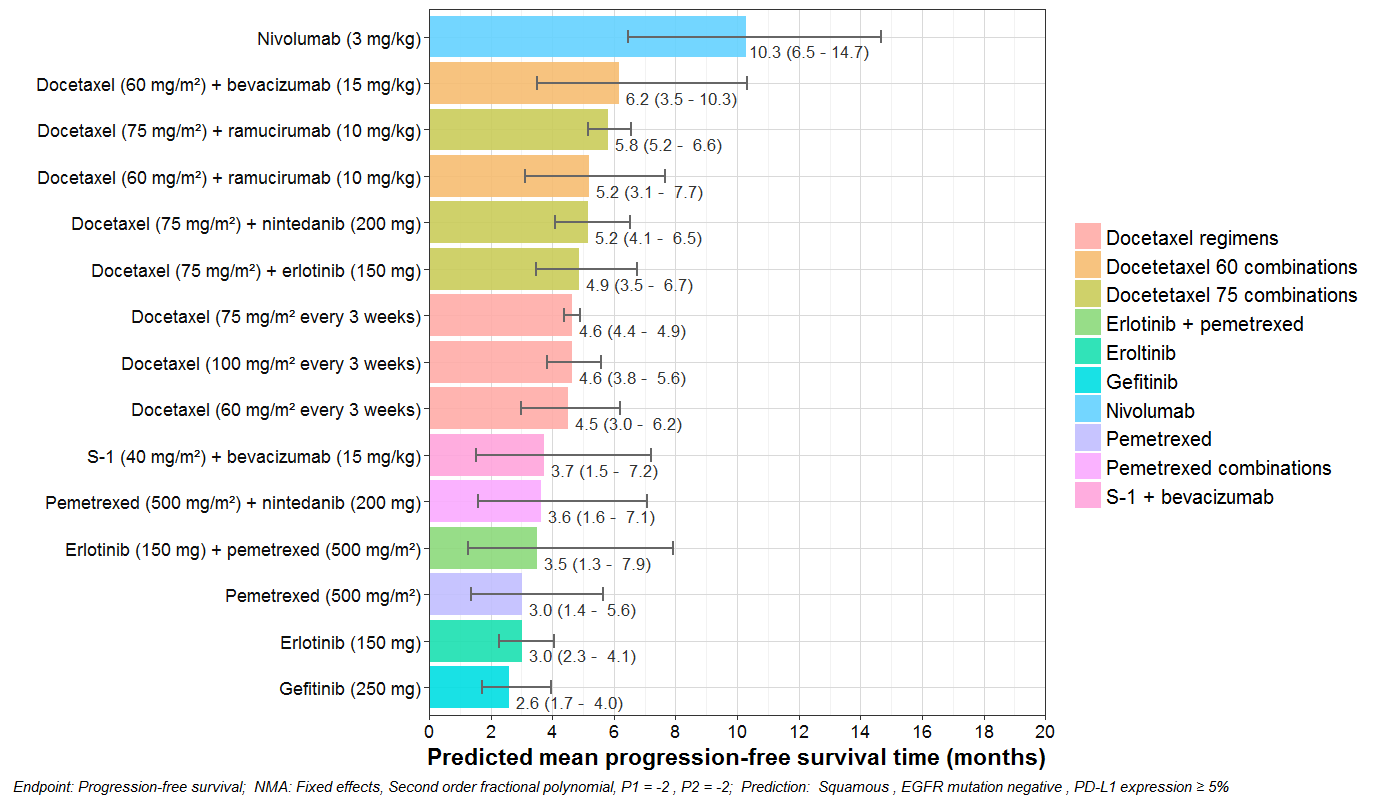

Figure S24. All Pairwise Comparisons of Mean Progression-Free Survival Times (Months): Squamous, PD-L1 Expression ≥ 5%, EGFR Mutation Negative

## Results for Nonsquamous, PD-L1 Expression < 5%, EGFR Mutation Positive


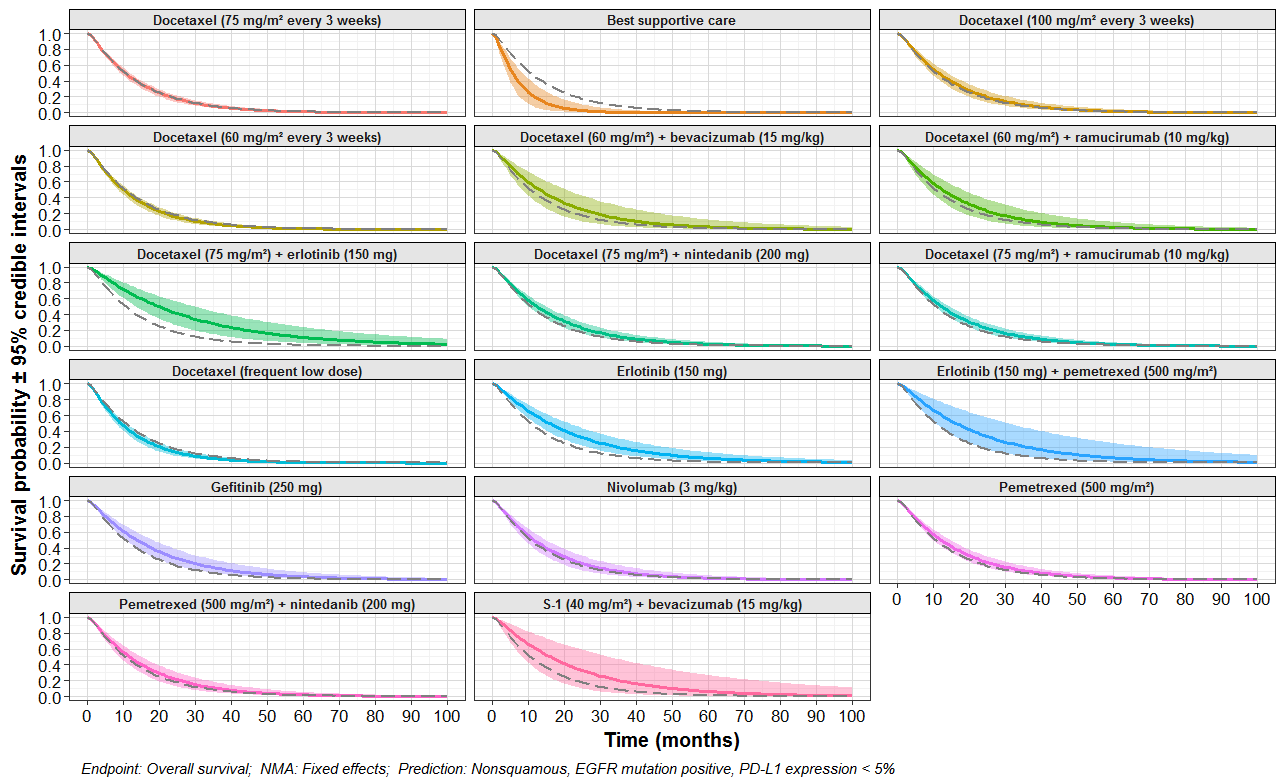
Figure S25. Probability of Overall Survival Curves: Nonsquamous, PD-L1 Expression < 5%, EGFR Mutation Positive

Note: Dotted line represents docetaxel (75 mg/m^2^ every 3 weeks).

Figure S26. Predicted Mean Overall Survival Time (Months): Nonsquamous, PD-L1 Expression < 5%, EGFR Mutation Positive


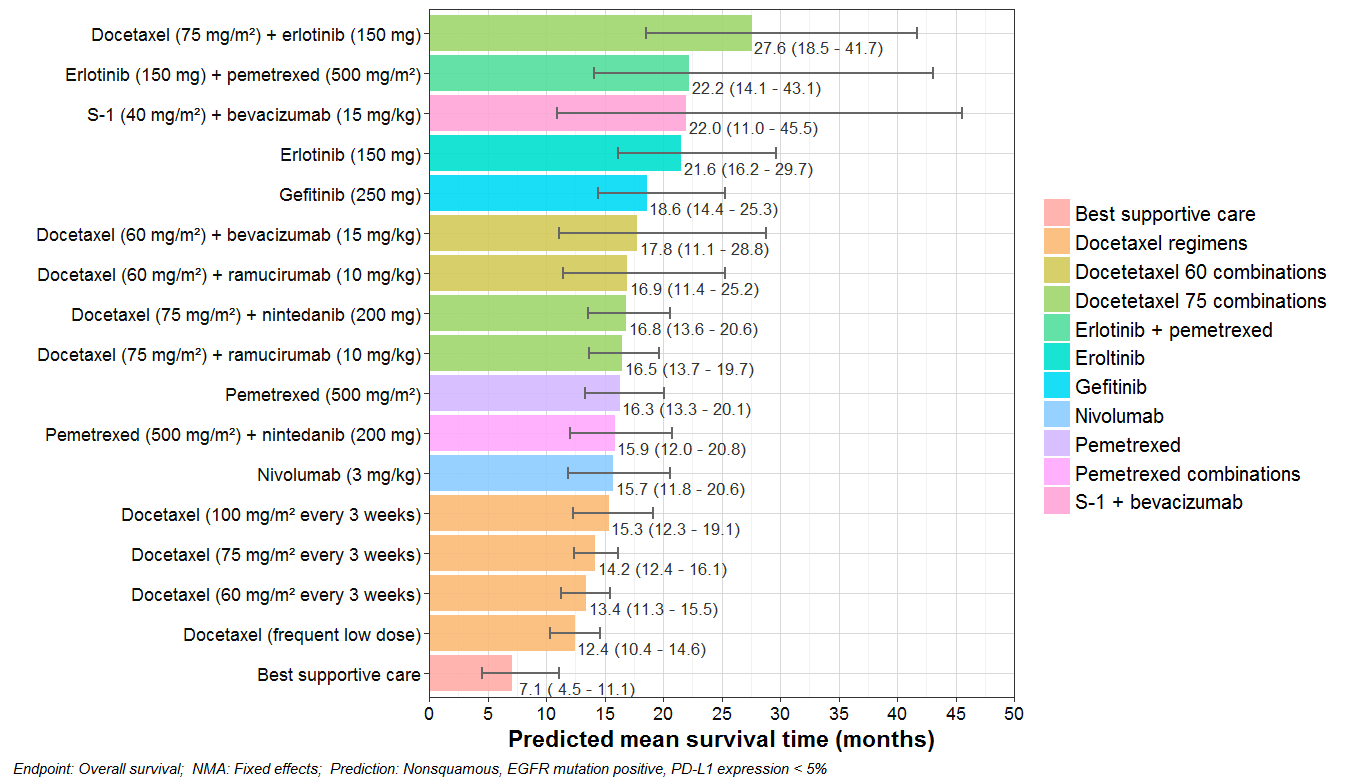

Figure S27. All Pairwise Comparisons of Mean Overall Survival Times (Months): Nonsquamous, PD-L1 Expression < 5%, EGFR Mutation Positive


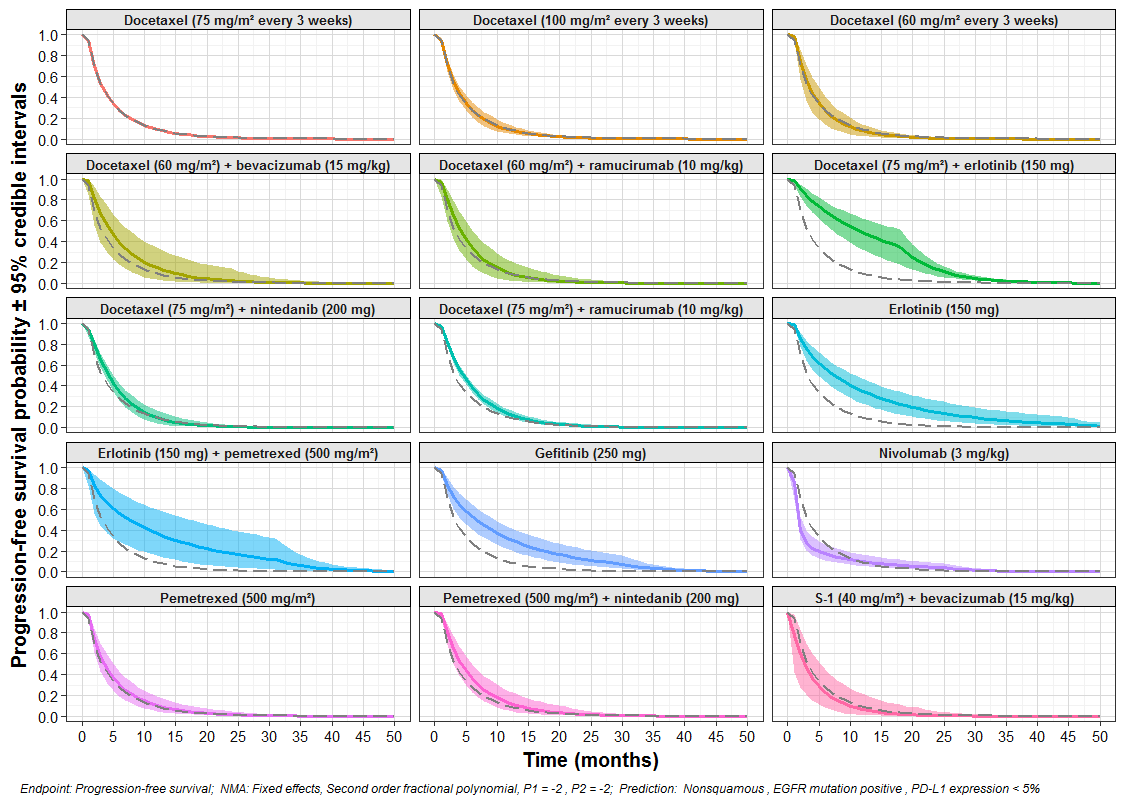
Figure S28. Probability of Progression-Free Survival Curves: Nonsquamous, PD-L1 Expression < 5%, EGFR Mutation Positive

Note: Dotted line represents docetaxel (75 mg/m^2^ every 3 weeks).

Figure S29. Predicted Mean Progression-Free Survival Time (Months): Nonsquamous, PD-L1 Expression < 5%, EGFR Mutation Positive


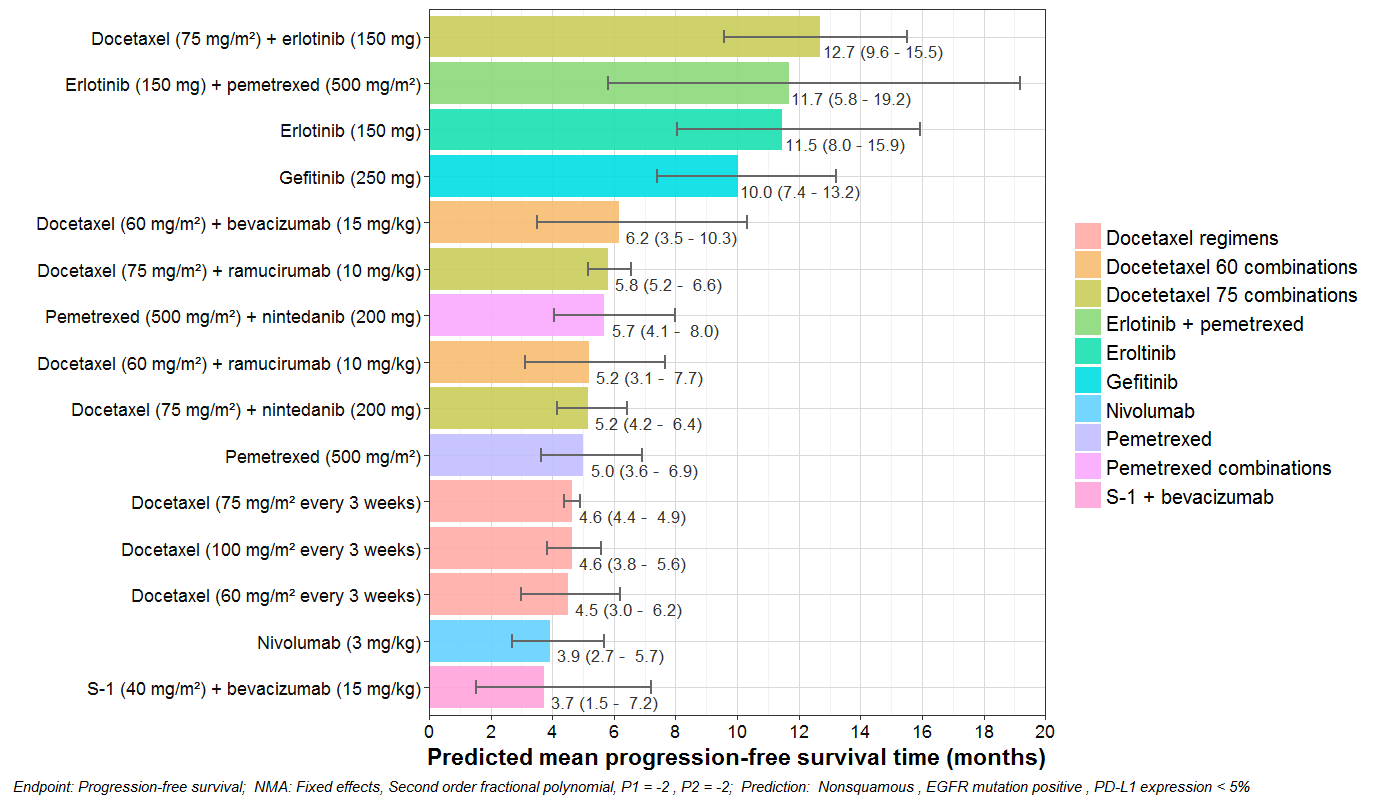

Figure S30. All Pairwise Comparisons of Mean Progression-Free Survival Times (Months): Nonsquamous, PD-L1 Expression < 5%, EGFR Mutation Positive

## Results for Squamous, PD-L1 Expression < 5%, EGFR Mutation Positive

Figure S31. Probability of Overall Survival Curves: Squamous, PD-L1 Expression < 5%, EGFR Mutation Positive


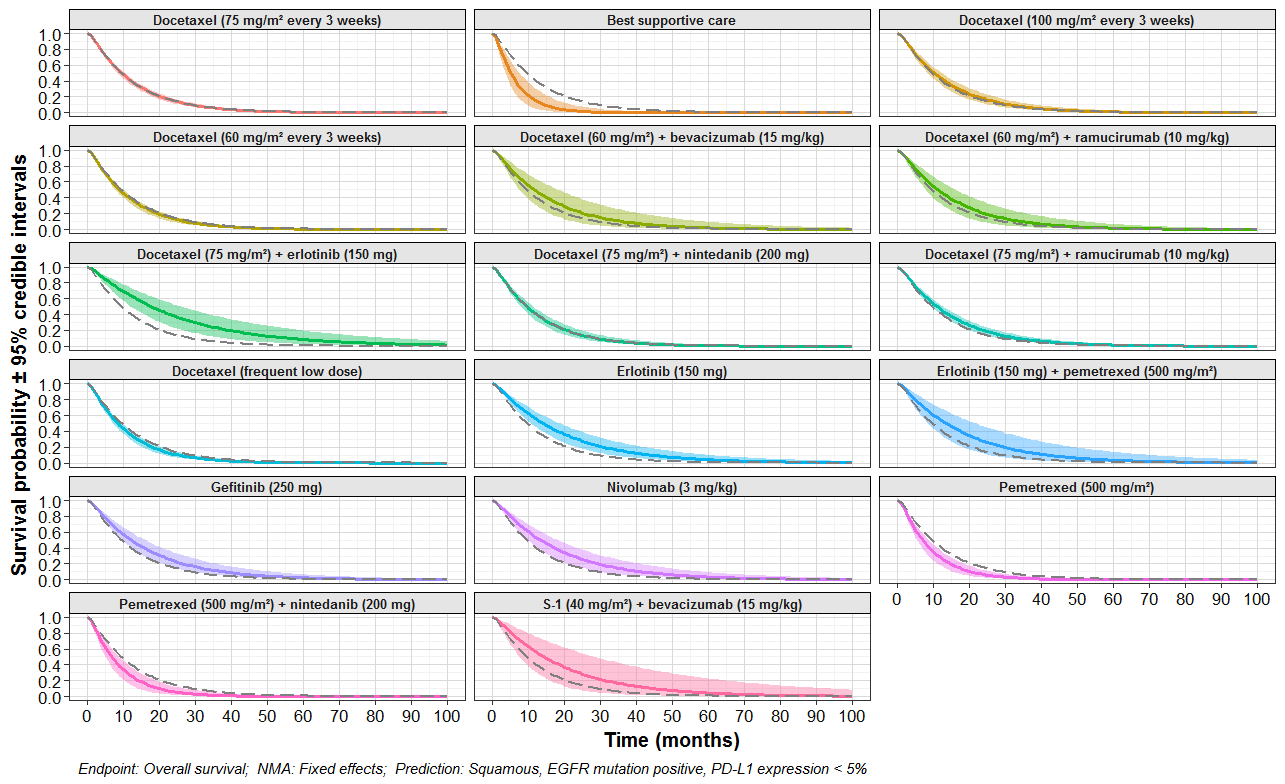


Note: Dotted line represents docetaxel (75 mg/m^2^ every 3 weeks).

Figure S32. Predicted Mean Overall Survival Time (Months): Squamous, PD-L1 Expression < 5%, EGFR Mutation Positive


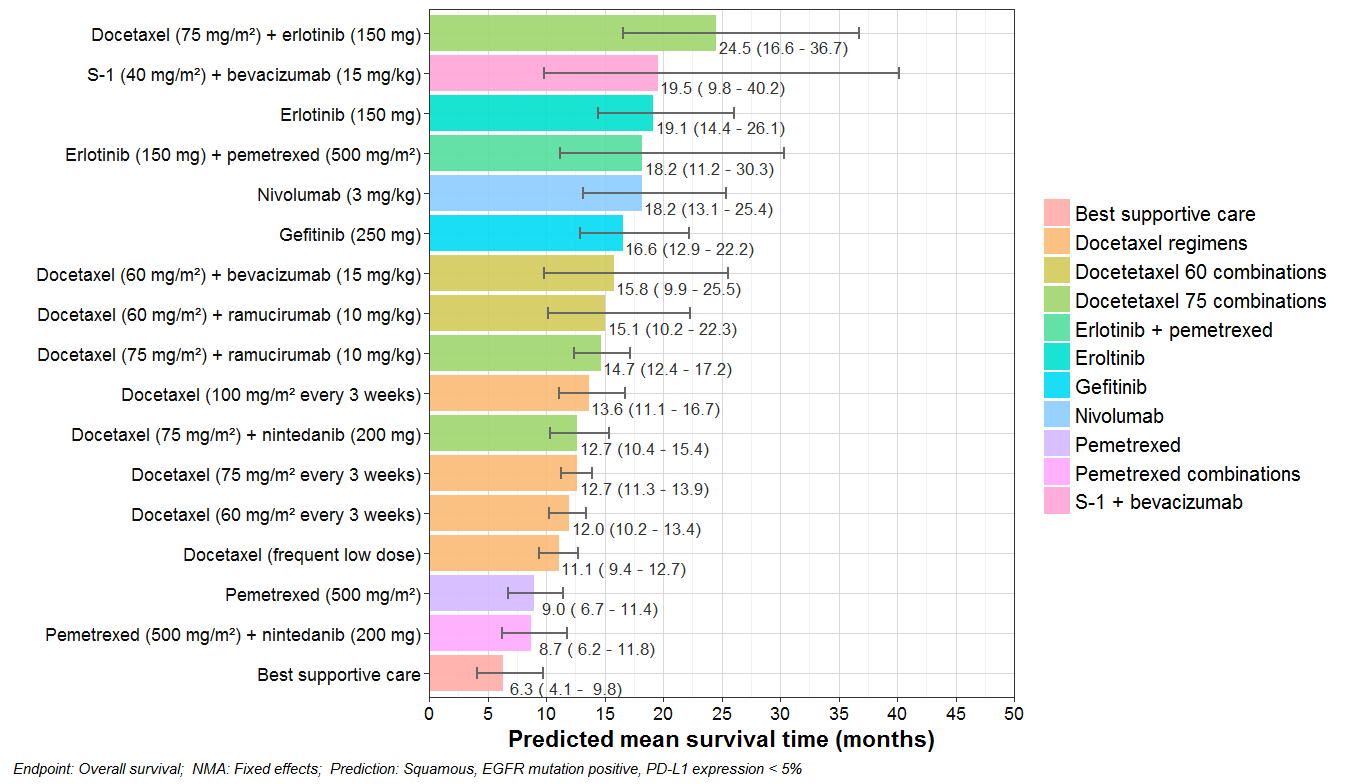

Figure S33. All Pairwise Comparisons of Mean Overall Survival Times (Months): Squamous, PD-L1 Expression < 5%, EGFR Mutation Positive


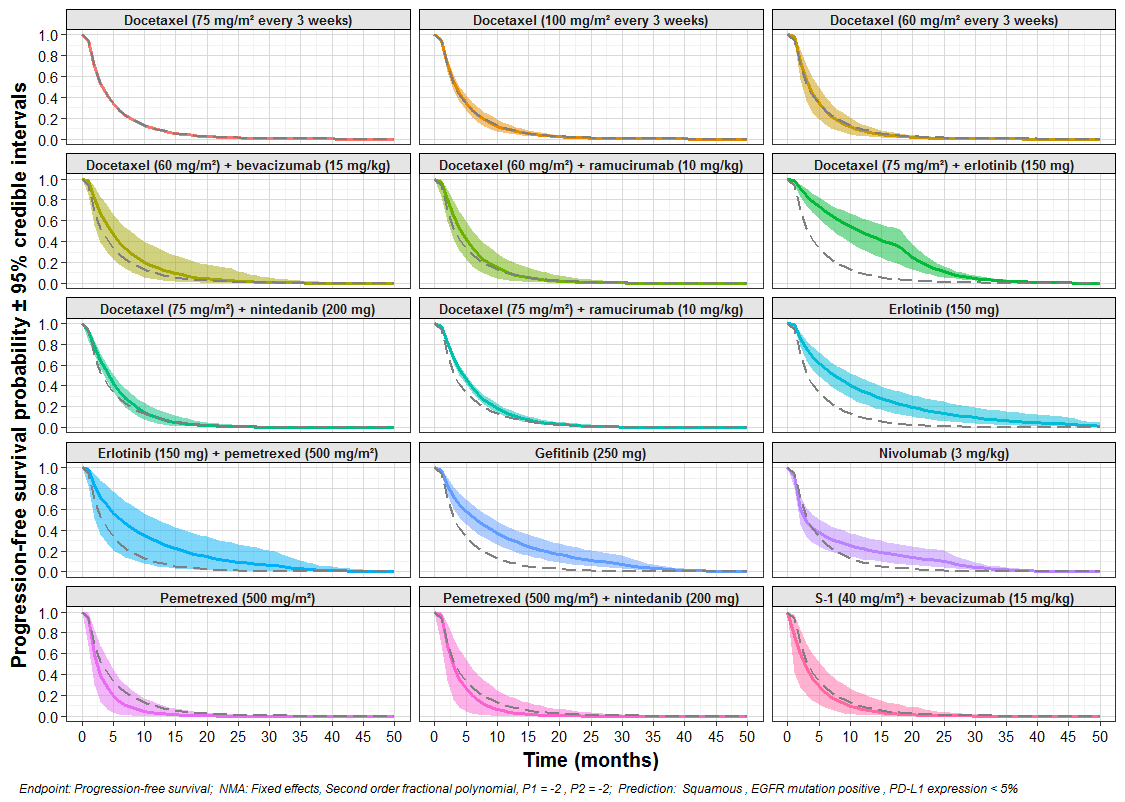
Figure S34. Probability of Progression-Free Survival Curves: Squamous, PD-L1 Expression < 5%, EGFR Mutation Positive

Note: Dotted line represents docetaxel (75 mg/m^2^ every 3 weeks).

Figure S35. Predicted Mean Progression-Free Survival Time (Months): Squamous, PD-L1 Expression < 5%, EGFR Mutation Positive


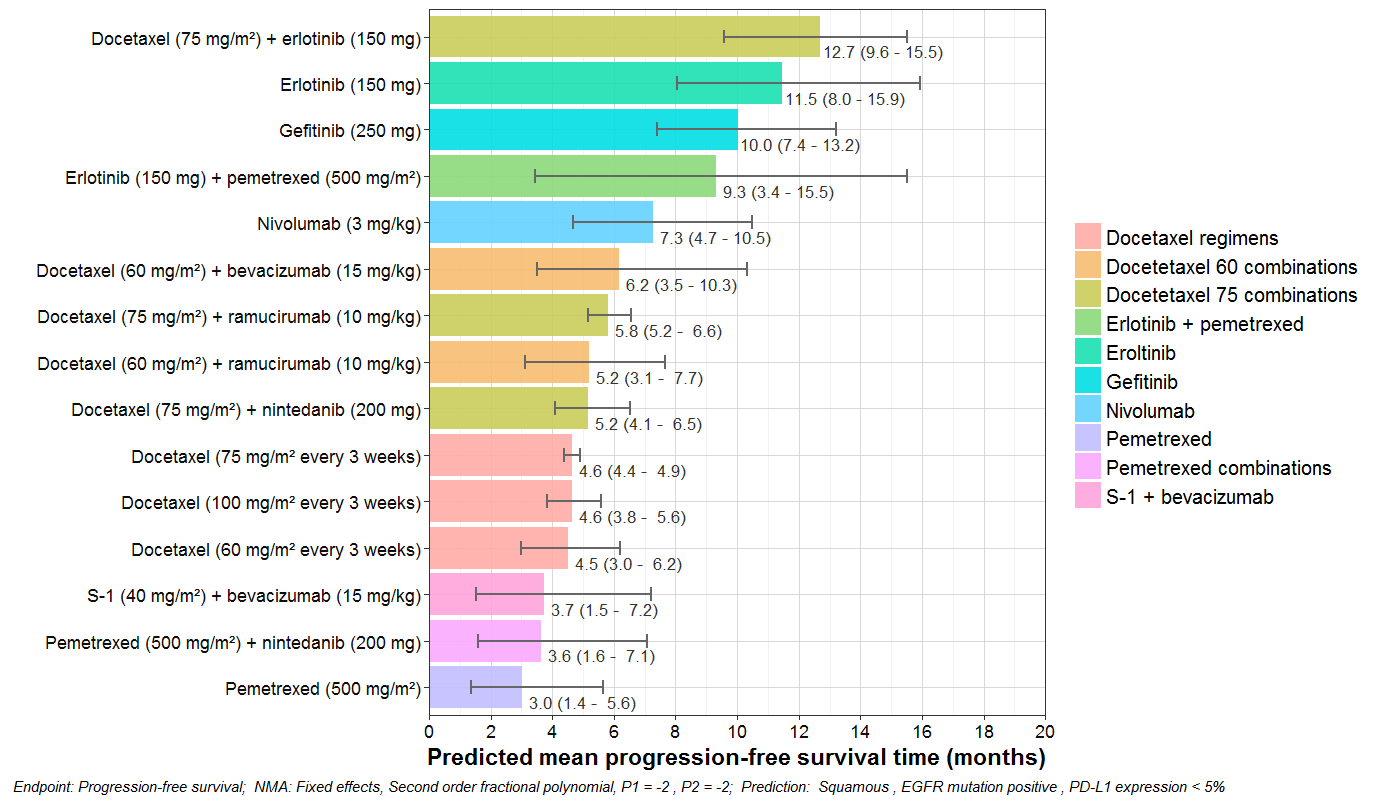

Figure S36. All Pairwise Comparisons of Mean Progression-Free Survival Times (Months): Squamous, PD-L1 Expression < 5%, EGFR Mutation Positive

## Results for Nonsquamous, PD-L1 Expression ≥ 5%, EGFR Mutation Positive

Figure S37. Probability of Overall Survival Curves: Nonsquamous, PD-L1 Expression ≥ 5%, EGFR Mutation Positive


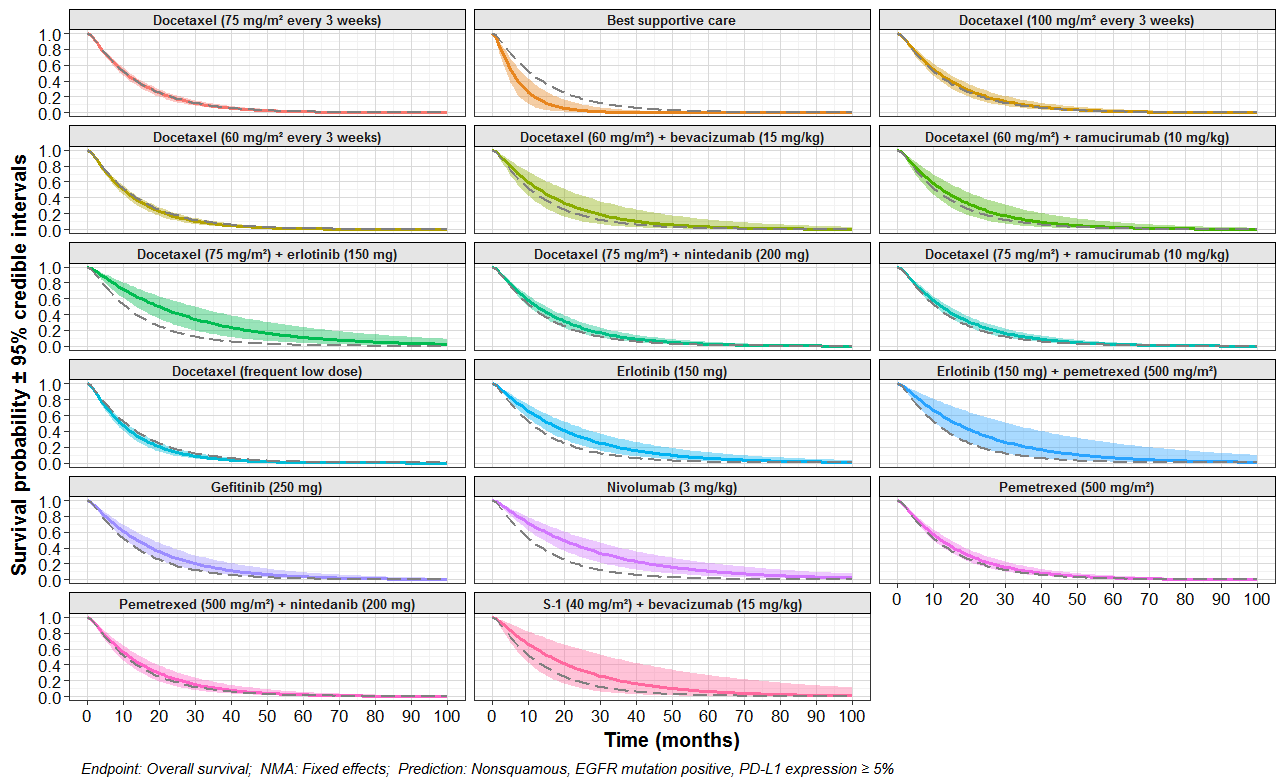


Note: Dotted line represents docetaxel (75 mg/m^2^ every 3 weeks).

Figure S38. Predicted Mean Overall Survival Time (Months): Nonsquamous, PD-L1 Expression ≥ 5%, EGFR Mutation Positive


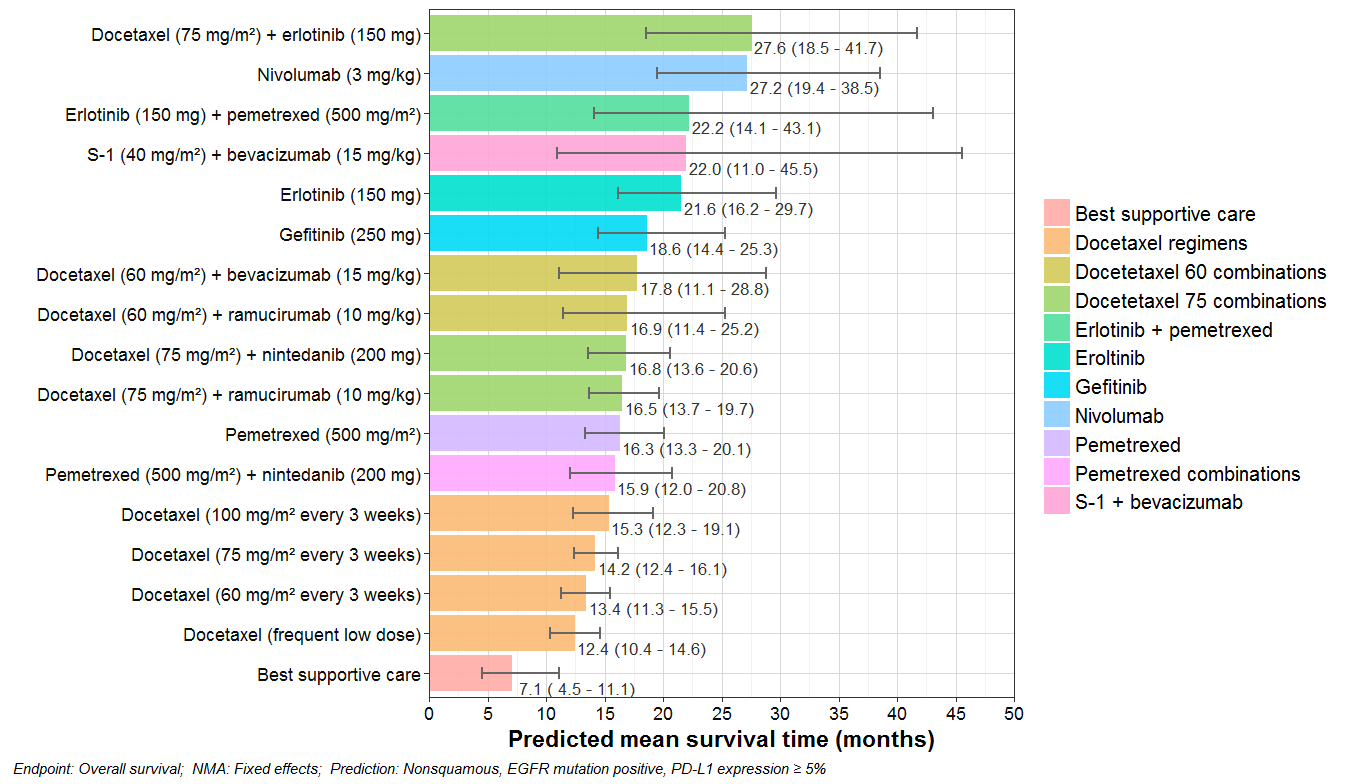

Figure S39. All Pairwise Comparisons of Mean Overall Survival Times (Months): Nonsquamous, PD-L1 Expression ≥ 5%, EGFR Mutation Positive


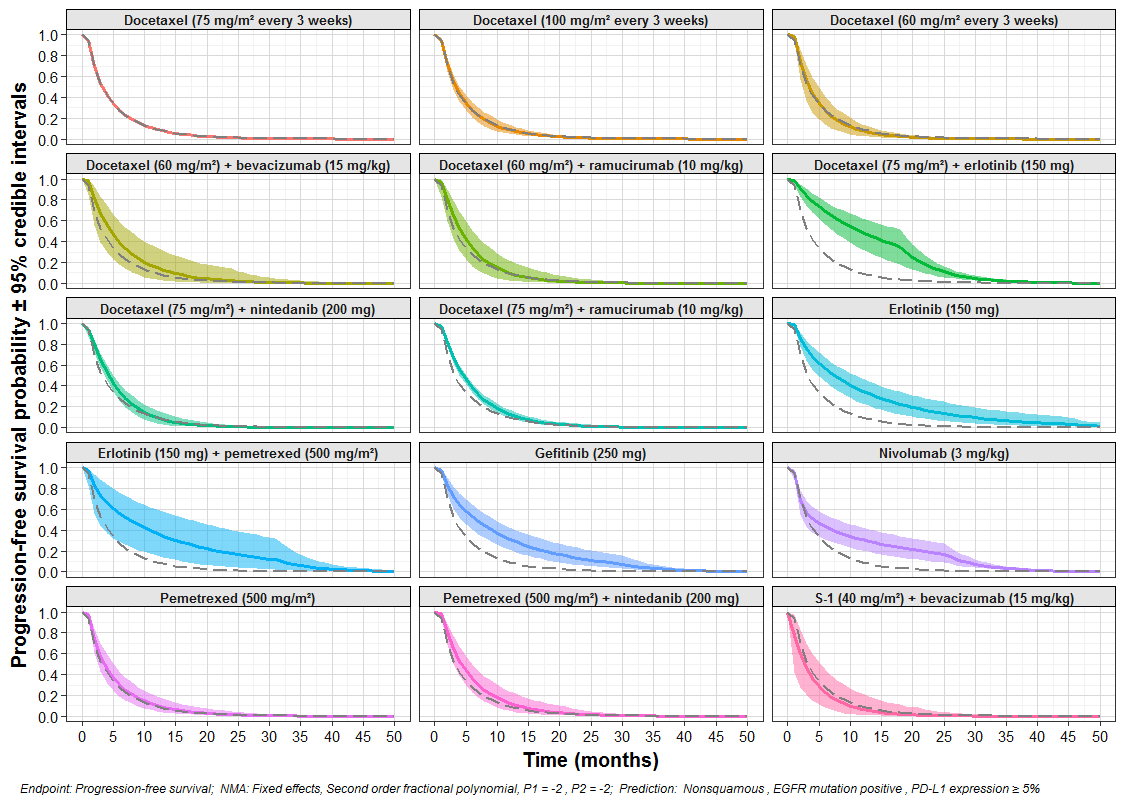
Figure S40. Probability of Progression-Free Survival Curves: Nonsquamous, PD-L1 Expression ≥ 5%, EGFR Mutation Positive

Note: Dotted line represents docetaxel (75 mg/m^2^ every 3 weeks).


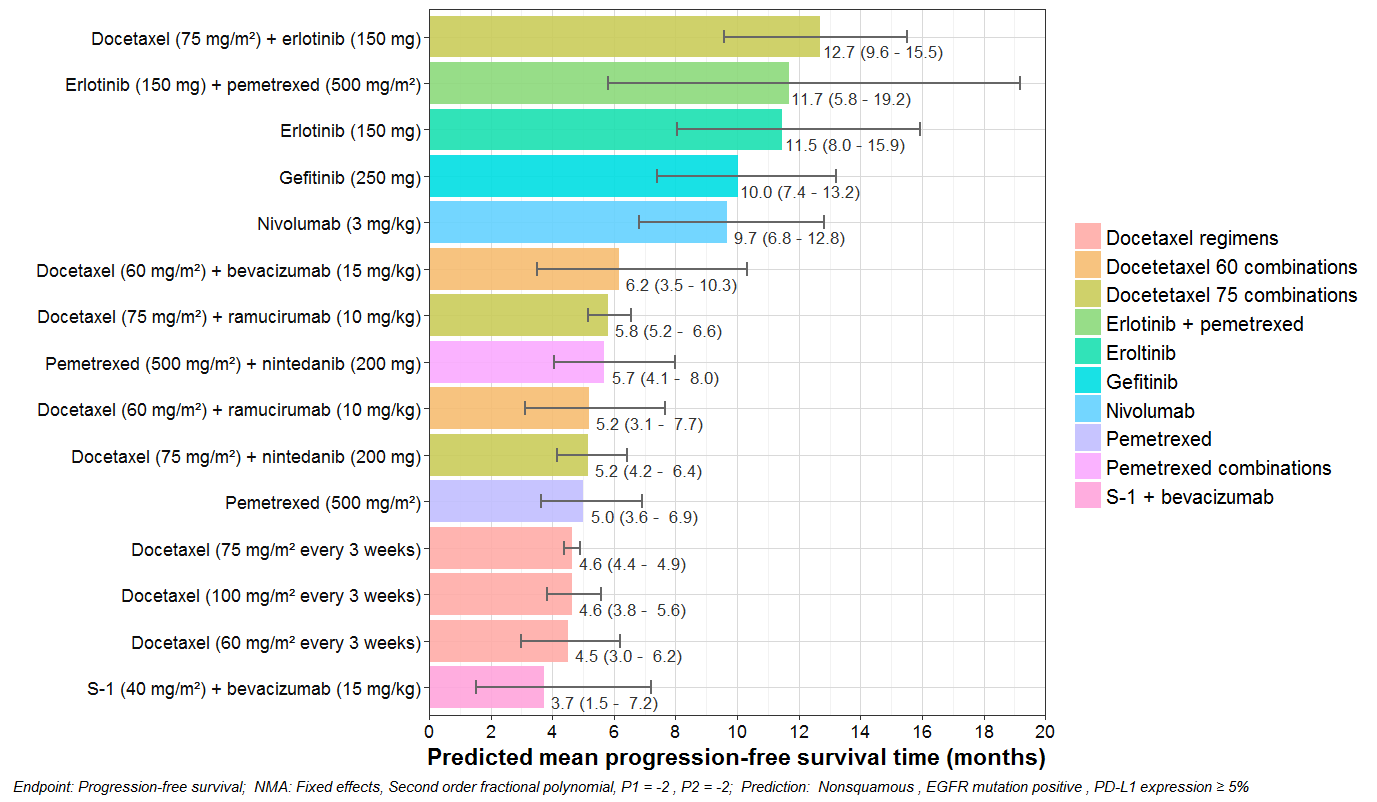
Figure S41. Predicted Mean Progression-Free Survival Time (Months): Nonsquamous, PD-L1 Expression ≥ 5%, EGFR Mutation Positive

Figure S42. All Pairwise Comparisons of Mean Progression-Free Survival Times (Months): Nonsquamous, PD-L1 Expression ≥ 5%, EGFR Mutation Positive

## Results for Squamous, PD-L1 Expression ≥ 5%, EGFR Mutation Positive

Figure S43. Probability of Overall Survival Curves: Squamous, PD-L1 Expression ≥ 5%, EGFR Mutation Positive


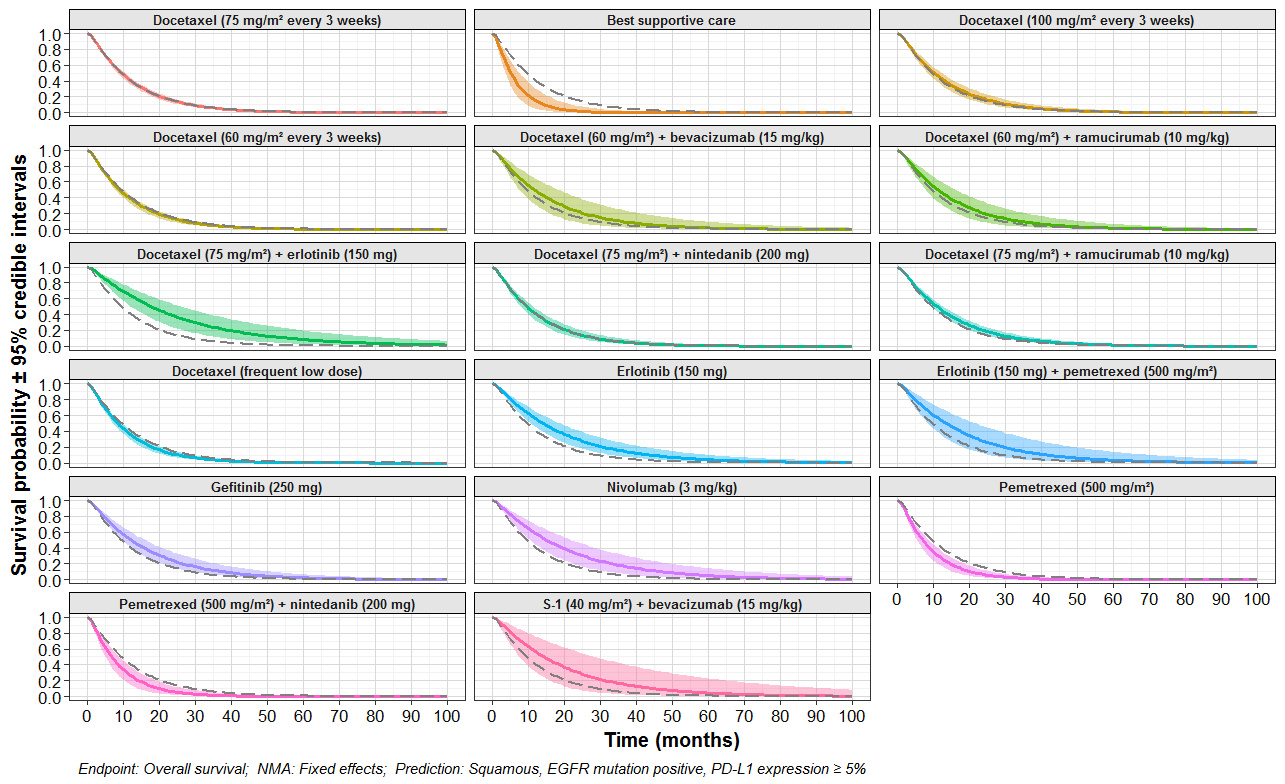


Note: Dotted line represents docetaxel (75 mg/m^2^ every 3 weeks).

Figure S44. Predicted Mean Overall Survival Time (Months): Squamous, PD-L1 Expression ≥ 5%, EGFR Mutation Positive


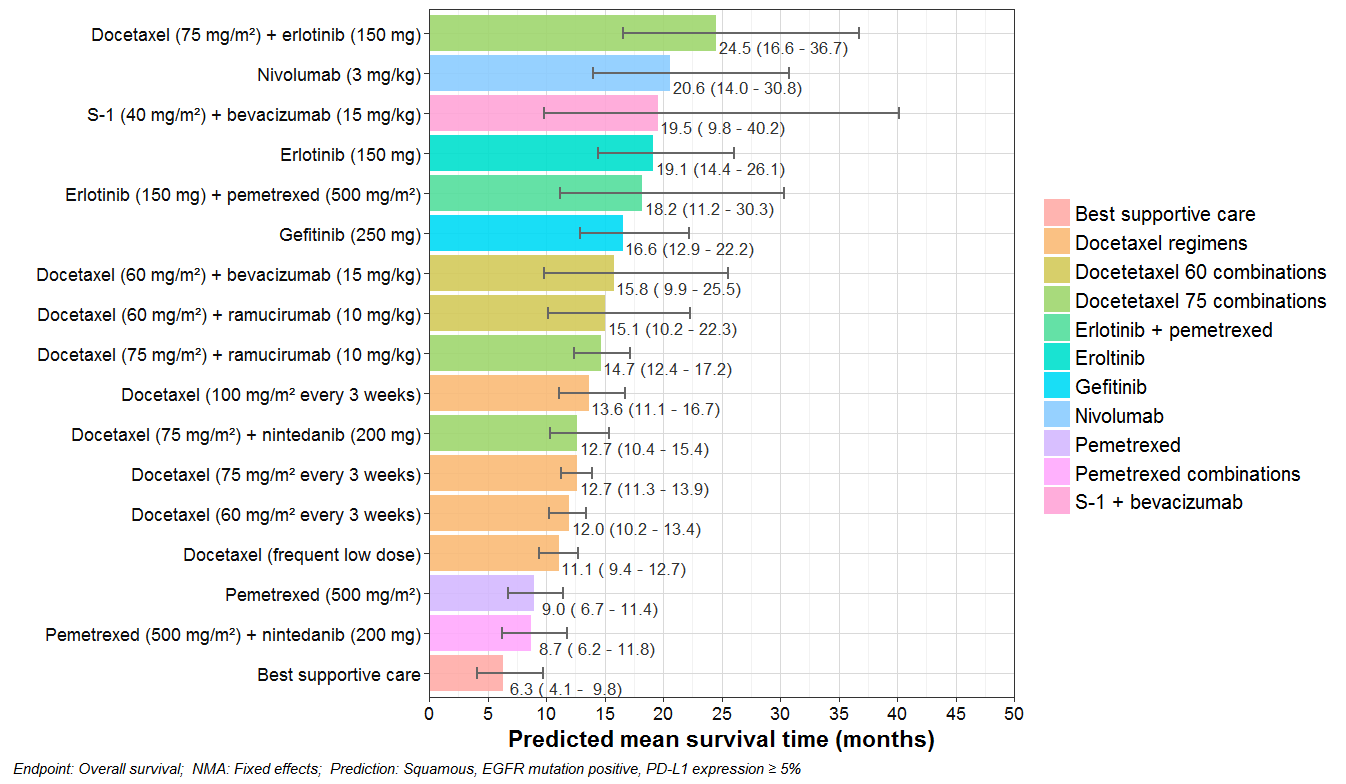

Figure S45. All Pairwise Comparisons of Mean Overall Survival Times (Months): Squamous, PD-L1 Expression ≥ 5%, EGFR Mutation Positive


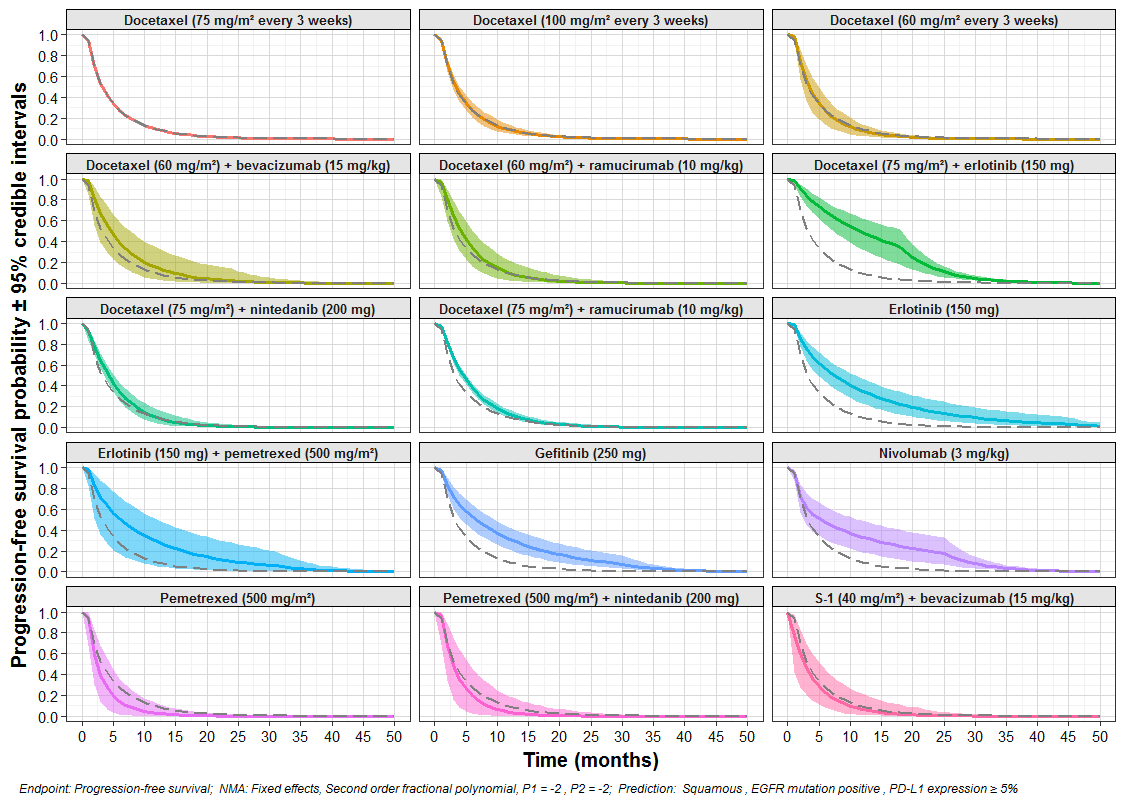
Figure S46. Probability of Progression-Free Survival Curves: Squamous, PD-L1 Expression ≥ 5%, EGFR Mutation Positive

Note: Dotted line represents docetaxel (75 mg/m^2^ every 3 weeks).


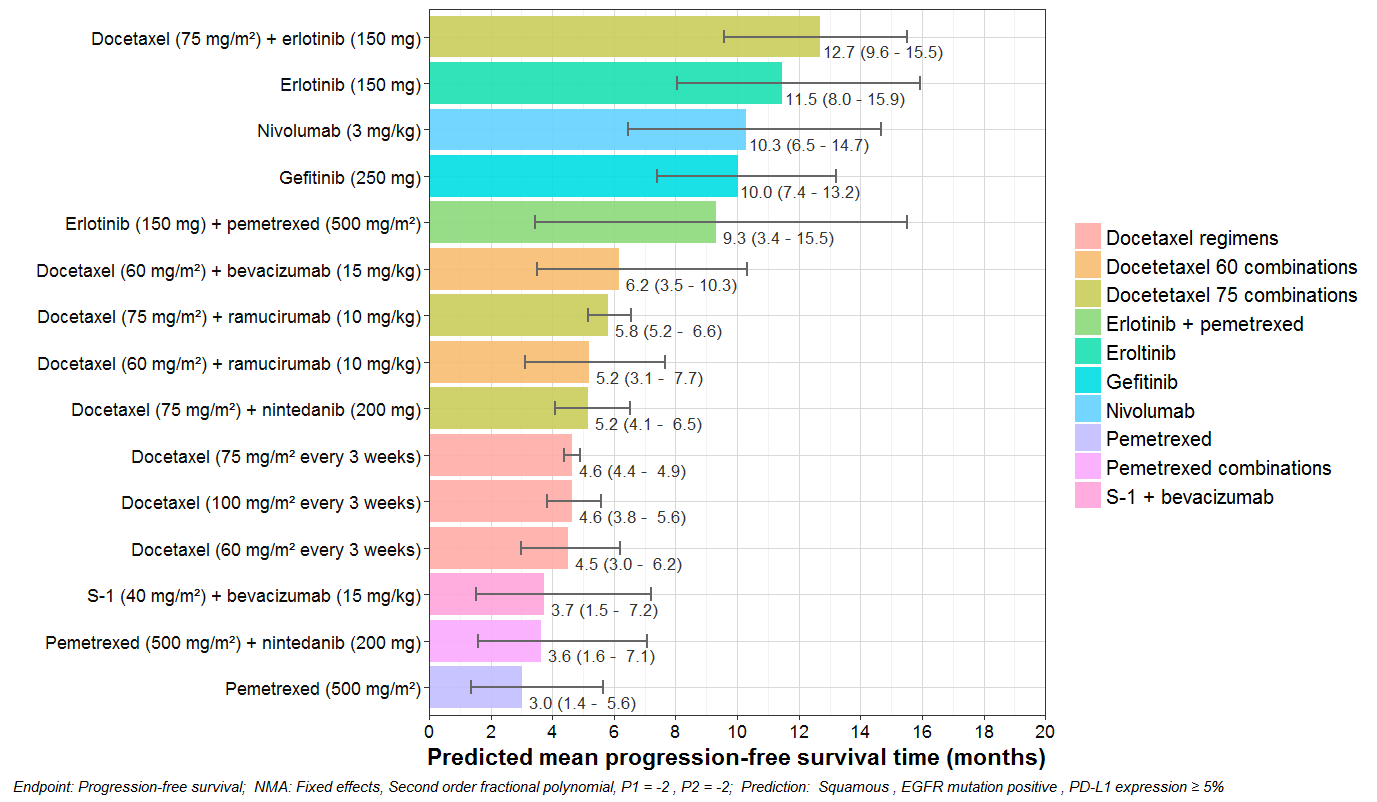
Figure S47. Predicted Mean Progression-Free Survival Time (Months): Squamous, PD-L1 Expression ≥ 5%, EGFR Mutation Positive

Figure S48. All Pairwise Comparisons of Mean Progression-Free Survival Times (Months): Squamous, PD-L1 Expression ≥ 5%, EGFR Mutation Positive

## JAGS Code Used for the Reference Treatment for Overall Survival: First-Order Fractional Polynomial Survival Model With Random Effects for Shape and Scale and Correlation Between These Parameters

Adapted from Jansen [12] for the fractional polynomial survival model and Gelman and Hill [78] for a random intercept and slope model. The model also includes two covariates, which had been centered.

cat( “

model {

for (i in 1:N) { # N number of data points in dataset

# time is expressed in months and transformed according powers of fractional polynomial P1 and P2

time_transf1[i] <- (equals(P1,0)*log(time[i]) + (1-equals(P1,0))*pow(time[i],P1))

# likelihood

# hazard over interval [t,t+dt] expressed as deaths per person-month

# r is deaths in interval, n is number at risk, h is hazard

r[i]~ dbin(p[i],n[i])

p[i] <- 1-exp(-h[i]*dt[i]) # dt=time interval cumulative hazard over interval[t,t+dt] expressed as deaths per person-month

# loop over data points

# s refers to study

log(h[i]) <- Beta.1[i] + Beta.2[i]*time_transf1[i]

Beta.1[i] <- mu.1[s[i]] + (beta.x1 * covariate.1[i]) + (beta.x2 * covariate.2[i])

Beta.2[i] <- mu.2[s[i]]

}

for(k in 1:NS) {

mu.1[k] <- B[k,1]

mu.2[k] <- B[k,2]

B[k,1:2] ~ dmnorm(B.hat[k,], Tau.B[,])

B.hat[k,1] <- mu.int.1

B.hat[k,2] <- mu.int.2

}

mu.int.1~dnorm(0,0.0001)

mu.int.2~dnorm(0,0.0001)

Tau.B[1:2,1:2] <- inverse(Sigma.B[,])

Sigma.B[1,1] <- pow(sigma.int.1,2)

sigma.int.1 ~ dunif(0,100)

Sigma.B[2,2] <- pow(sigma.int.2,2)

sigma.int.2 ~ dunif(0,100)

Sigma.B[1,2] <- rho*sigma.int.1*sigma.int.2

Sigma.B[2,1] <- Sigma.B[1,2]

rho ~ dunif(-1,1)

beta.x1 ~ dnorm(0,.0001)

beta.x2 ~ dnorm(0,.0001)

}

## JAGS Code Used for Overall Survival: Fixed-Effects Hazard Ratio Model With Hierarchical Exchangeable Structures

Adapted from Woods et al. [29] and the hierarchical exchangeable model presented by Owen et al. [31].

cat( “

# set indicator variables to be 1

# datablock required for JAGS due to re-using objects with the same name - different from WinBUGS

data{

b1 <- 1

b2 <- 1

b3 <- 1

b4 <- 1

b5 <- 1

b6 <- 1

b7 <- 1

b8 <- 1

b9 <- 1

b10 <- 1

b11 <- 1

b12 <- 1

b13 <- 1

b14 <- 1

b15 <- 1

b16 <- 1

b17 <- 1

b18 <- 1

}

model{

d[1] <- 0

#On individual study baseline effect

for(ss in 1:nStudies){

alpha[ss] ~ dnorm(0,1.0E-6)

}

#Fit data

#For hazard ratio reporting studies

for(ii in 1:LnObs ){

Lmu[ii] <- alpha[Lstudy[ii]]*multi[ii] + d[Ltx[ii]] - d[Lbase[ii]]

Lprec[ii] <- 1/pow(Lse[ii],2)

Lmean[ii] ~ dnorm(Lmu[ii],Lprec[ii])

}

# Adding in hierarchical model where class of intervention is above intervention in the hierarchy

d[2] ~ dnorm(D.d[1], prec.d) # best.supportive.care

d[3] ~ dnorm(D.d[2], prec.d) # docetaxel.100

d[4] ~ dnorm(D.d[2], prec.d) # docetaxel.60

d[5] ~ dnorm(D.d[3], prec.d) # docetaxel.60_bevacizumab

d[6] ~ dnorm(D.d[4], prec.d) # docetaxel.60_ramucirumab

d[7] ~ dnorm(D.d[5], prec.d) # docetaxel.75_erlotinib.150

d[8] ~ dnorm(D.d[6], prec.d) # docetaxel.75_nintedanib.non

d[9] ~ dnorm(D.d[6], prec.d) # docetaxel.75_nintedanib.sq

d[10] ~ dnorm(D.d[4], prec.d) # docetaxel.75_ramucirumab

d[11] ~ dnorm(D.d[2], prec.d) # docetaxel.fr

d[12] ~ dnorm(D.d[7], prec.d) # erlotinib.150_0.00.pos

d[13] ~ dnorm(D.d[7], prec.d) # erlotinib.150_0.03.pos

d[14] ~ dnorm(D.d[7], prec.d) # erlotinib.150_0.05.pos

d[15] ~ dnorm(D.d[7], prec.d) # erlotinib.150_0.09.pos

d[16] ~ dnorm(D.d[7], prec.d) # erlotinib.150_0.16.pos

d[17] ~ dnorm(D.d[7], prec.d) # erlotinib.150_0.56.pos

d[18] ~ dnorm(D.d[7], prec.d) # erlotinib.150_1.00.pos

d[19] ~ dnorm(D.d[8], prec.d) # erlotinib.150_pemetrexed.500_0.00.pos

d[20] ~ dnorm(D.d[8], prec.d) # erlotinib.150_pemetrexed.500_0.56.pos

d[21] ~ dnorm(D.d[9], prec.d) # gefitinib.250_0.00.pos

d[22] ~ dnorm(D.d[9], prec.d) # gefitinib.250_0.06.pos

d[23] ~ dnorm(D.d[9], prec.d) # gefitinib.250_0.15.pos

d[24] ~ dnorm(D.d[9], prec.d) # gefitinib.250_0.47.pos

d[25] ~ dnorm(D.d[9], prec.d) # gefitinib.250_1.00.pos

d[26] ~ dnorm(D.d[10], prec.d) # nivolumab_non.sq_high.PDL1

d[27] ~ dnorm(D.d[10], prec.d) # nivolumab_non.sq_low.PDL1

d[28] ~ dnorm(D.d[10], prec.d) # nivolumab_sq_high.PDL1

d[29] ~ dnorm(D.d[10], prec.d) # nivolumab_sq_low.PDL1

d[30] ~ dnorm(D.d[11], prec.d) # pemetrexed.500_0.00.sq

d[31] ~ dnorm(D.d[11], prec.d) # pemetrexed.500_0.19.sq

d[32] ~ dnorm(D.d[11], prec.d) # pemetrexed.500_0.23.sq

d[33] ~ dnorm(D.d[11], prec.d) # pemetrexed.500_0.25.sq

d[34] ~ dnorm(D.d[11], prec.d) # pemetrexed.500_0.26.sq

d[35] ~ dnorm(D.d[11], prec.d) # pemetrexed.500_1.00.sq

d[36] ~ dnorm(D.d[12], prec.d) # pemetrexed.500_nintedanib

d[37] ~ dnorm(D.d[13], prec.d) # S1_bevacizumab

# Placing ordering constraints

# constraints for erlotinib and EGFR +ve

gamma1 <- step(d[12] - d[13])

gamma2 <- step(d[13] - d[14])

gamma3 <- step(d[14] - d[15])

gamma4 <- step(d[15] - d[16])

gamma5 <- step(d[16] - d[17])

gamma6 <- step(d[17] - d[18])

# constraints for gefitinib and EGFR +ve

gamma7 <- step(d[21] - d[22])

gamma8 <- step(d[22] - d[23])

gamma9 <- step(d[23] - d[24])

gamma10 <- step(d[24] - d[25])

# constraints for pemetrexed and squamous

gamma11 <- step(d[35] - d[34])

gamma12 <- step(d[34] - d[33])

gamma13 <- step(d[33] - d[32])

gamma14 <- step(d[32] - d[31])

gamma15 <- step(d[31] - d[30])

# constraints for erlotinib_pemetrexed and EGFR +ve

gamma16 <- step(d[19] - d[20])

# constraints for docetaxel

gamma17 <- step(d[11] - d[4])

gamma18 <- step(d[4]) # docetaxel.60 versus reference

# define binomial prior distributions

b1 ~ dbern(gamma1)

b2 ~ dbern(gamma2)

b3 ~ dbern(gamma3)

b4 ~ dbern(gamma4)

b5 ~ dbern(gamma5)

b6 ~ dbern(gamma6)

b7 ~ dbern(gamma7)

b8 ~ dbern(gamma8)

b9 ~ dbern(gamma9)

b10 ~ dbern(gamma10)

b11 ~ dbern(gamma11)

b12 ~ dbern(gamma12)

b13 ~ dbern(gamma13)

b14 ~ dbern(gamma14)

b15 ~ dbern(gamma15)

b16 ~ dbern(gamma16)

b17 ~ dbern(gamma17)

b18 ~ dbern(gamma18)

prec.d <- 1/(sd.d*sd.d)

sd.d ~ dunif(0,5)

for (i in 1:13){

D.d[i]~dnorm(0.0,0.001) } # vague priors on class effects

hr[1] <- 1

# Calculate HRs

hr[1] <- 1

for (hh in 2:nTx) {

hr[hh] <- exp(d[hh])

}

}

## JAGS Code Used for Overall Survival: Random-Effects Hazard Ratio Model With Hierarchical Exchangeable Structures

Adapted from Woods et al. [29] and the hierarchical exchangeable model presented by Owen et al. [31].

cat( “

# set indicator variables to be 1

# datablock required for JAGS due to re-using objects with the same name - different from WinBUGS

data{

b1 <- 1

b2 <- 1

b3 <- 1

b4 <- 1

b5 <- 1

b6 <- 1

b7 <- 1

b8 <- 1

b9 <- 1

b10 <- 1

b11 <- 1

b12 <- 1

b13 <- 1

b14 <- 1

b15 <- 1

b16 <- 1

b17 <- 1

b18 <- 1

}

model{

#Define Prior Distributions

#on random tx effect variance

sd ~ dunif(0,5)

reTau <- 2/pow(sd,2)

#On individual study baseline effect

for(ss in 1:nStudies){

alpha[ss] ~ dnorm(0,1.0E-6)

}

#Define random effect

for (ss in 1:nStudies){

for(tt in 1:nTx){

re[ss,tt]~dnorm(0,reTau)

}

}

#Fit data

#For hazard ratio reporting studies

for(ii in 1:LnObs ){

Lmu[ii] <- alpha[Lstudy[ii]]*multi[ii] + re[Lstudy[ii],Ltx[ii]] -re[Lstudy[ii],Lbase[ii]] + d[Ltx[ii]] - d[Lbase[ii]]

Lprec[ii] <- 1/pow(Lse[ii],2)

Lmean[ii] ~ dnorm(Lmu[ii],Lprec[ii])

}

# Adding in hierarchical model where class of intervention is above intervention in the hierarchy

d[2] ~ dnorm(D.d[1], prec.d) # best.supportive.care

d[3] ~ dnorm(D.d[2], prec.d) # docetaxel.100

d[4] ~ dnorm(D.d[2], prec.d) # docetaxel.60

d[5] ~ dnorm(D.d[3], prec.d) # docetaxel.60_bevacizumab

d[6] ~ dnorm(D.d[4], prec.d) # docetaxel.60_ramucirumab

d[7] ~ dnorm(D.d[5], prec.d) # docetaxel.75_erlotinib.150

d[8] ~ dnorm(D.d[6], prec.d) # docetaxel.75_nintedanib.non

d[9] ~ dnorm(D.d[6], prec.d) # docetaxel.75_nintedanib.sq

d[10] ~ dnorm(D.d[4], prec.d) # docetaxel.75_ramucirumab

d[11] ~ dnorm(D.d[2], prec.d) # docetaxel.fr

d[12] ~ dnorm(D.d[7], prec.d) # erlotinib.150_0.00.pos

d[13] ~ dnorm(D.d[7], prec.d) # erlotinib.150_0.03.pos

d[14] ~ dnorm(D.d[7], prec.d) # erlotinib.150_0.05.pos

d[15] ~ dnorm(D.d[7], prec.d) # erlotinib.150_0.09.pos

d[16] ~ dnorm(D.d[7], prec.d) # erlotinib.150_0.16.pos

d[17] ~ dnorm(D.d[7], prec.d) # erlotinib.150_0.56.pos

d[18] ~ dnorm(D.d[7], prec.d) # erlotinib.150_1.00.pos

d[19] ~ dnorm(D.d[8], prec.d) # erlotinib.150_pemetrexed.500_0.00.pos

d[20] ~ dnorm(D.d[8], prec.d) # erlotinib.150_pemetrexed.500_0.56.pos

d[21] ~ dnorm(D.d[9], prec.d) # gefitinib.250_0.00.pos

d[22] ~ dnorm(D.d[9], prec.d) # gefitinib.250_0.06.pos

d[23] ~ dnorm(D.d[9], prec.d) # gefitinib.250_0.15.pos

d[24] ~ dnorm(D.d[9], prec.d) # gefitinib.250_0.52.pos

d[25] ~ dnorm(D.d[9], prec.d) # gefitinib.250_1.00.pos

d[26] ~ dnorm(D.d[10], prec.d) # nivolumab_non.sq_high.PDL1

d[27] ~ dnorm(D.d[10], prec.d) # nivolumab_non.sq_low.PDL1

d[28] ~ dnorm(D.d[10], prec.d) # nivolumab_sq_high.PDL1

d[29] ~ dnorm(D.d[10], prec.d) # nivolumab_sq_low.PDL1

d[30] ~ dnorm(D.d[11], prec.d) # pemetrexed.500_0.00.sq

d[31] ~ dnorm(D.d[11], prec.d) # pemetrexed.500_0.19.sq

d[32] ~ dnorm(D.d[11], prec.d) # pemetrexed.500_0.23.sq

d[33] ~ dnorm(D.d[11], prec.d) # pemetrexed.500_0.25.sq

d[34] ~ dnorm(D.d[11], prec.d) # pemetrexed.500_0.26.sq

d[35] ~ dnorm(D.d[11], prec.d) # pemetrexed.500_1.00.sq

d[36] ~ dnorm(D.d[12], prec.d) # pemetrexed.500_nintedanib

d[37] ~ dnorm(D.d[13], prec.d) # S1_bevacizumab

# Placing ordering constraints

# constraints for erlotinib and EGFR +ve

gamma1 <- step(d[12] - d[13])

gamma2 <- step(d[13] - d[14])

gamma3 <- step(d[14] - d[15])

gamma4 <- step(d[15] - d[16])

gamma5 <- step(d[16] - d[17])

gamma6 <- step(d[17] - d[18])

# constraints for gefitinib and EGFR +ve

gamma7 <- step(d[21] - d[22])

gamma8 <- step(d[22] - d[23])

gamma9 <- step(d[23] - d[24])

gamma10 <- step(d[24] - d[25])

# constraints for pemetrexed and squamous

gamma11 <- step(d[35] - d[34])

gamma12 <- step(d[34] - d[33])

gamma13 <- step(d[33] - d[32])

gamma14 <- step(d[32] - d[31])

gamma15 <- step(d[31] - d[30])

# constraints for erlotinib_pemetrexed and EGFR +ve

gamma16 <- step(d[19] - d[20])

# constraints for docetaxel and EGFR +ve

gamma17 <- step(d[11] - d[4])

gamma18 <- step(d[4]) # docetaxel.60 versus reference

# define binomial prior distributions

b1 ~ dbern(gamma1)

b2 ~ dbern(gamma2)

b3 ~ dbern(gamma3)

b4 ~ dbern(gamma4)

b5 ~ dbern(gamma5)

b6 ~ dbern(gamma6)

b7 ~ dbern(gamma7)

b8 ~ dbern(gamma8)

b9 ~ dbern(gamma9)

b10 ~ dbern(gamma10)

b11 ~ dbern(gamma11)

b12 ~ dbern(gamma12)

b13 ~ dbern(gamma13)

b14 ~ dbern(gamma14)

b15 ~ dbern(gamma15)

b16 ~ dbern(gamma16)

b17 ~ dbern(gamma17)

b18 ~ dbern(gamma18)

prec.d <- 1/(sd.d*sd.d)

sd.d ~ dunif(0,5)

for (i in 1:13){

D.d[i]~dnorm(0.0,0.001) } # vague priors on class effects

hr[1] <- 1

# Calculate HRs

for (hh in 2:nTx) {

hr[hh] <- exp(d[hh])

}

}

## JAGS Code Used for Progression-Free Survival: Second-Order Fixed-Effects Fractional Polynomial Survival Model With Hierarchical Exchangeable Structures

Adapted from Jansen [12] for fractional polynomial survival NMA and the hierarchical exchangeable model presented by Owen et al. [31].

cat( “

# set indicator variables to be 1

# datablock required for JAGS due to re-using objects with the same name - different from WinBUGS

data{

b1 <- 1

b2 <- 1

b3 <- 1

b4 <- 1

b5 <- 1

b6 <- 1

b7 <- 1

b8 <- 1

b9 <- 1

b10 <- 1

b11 <- 1

b12 <- 1

}

model {

for (i in 1:N) { # N number of data points in dataset

# time is expressed in months and transformed according powers of fractional polynomial P1 and P2

time_transf1[i] <- (equals(P1,0)*log(time[i]) + (1-equals(P1,0))*pow(time[i],P1))

time_transf2[i] <- ((1-equals(P2,P1))*(equals(P2,0)*log(time[i]) + (1-equals(P2,0))*pow(time[i],P2)) +

equals(P2,P1)*(equals(P2,0)*log(time[i])*log(time[i]) + (1-equals(P2,0))*pow(time[i],P2) *log(time[i])))

# likelihood

# hazard over interval [t,t+dt] expressed as deaths per person-month

# r is deaths in interval, n is number at risk, h is hazard

r[i]~ dbin(p[i],n[i])

p[i] <- 1-exp(-h[i]*dt[i]) # dt=time interval cumulative hazard over interval[t,t+dt] expressed as deaths per person-month

# fixed effects model

# loop over datapoints

# s refers to study, t is intervention t, b is comparator

log(h[i]) <- Beta[i,1] + Beta[i,2]*time_transf1[i] + Beta[i,3]*time_transf2[i]

Beta[i,1] <- mu[s[i],1] + d[t[i],1] - d[b[i],1]

Beta[i,2] <- mu[s[i],2] + d[t[i],2] - d[b[i],2]

Beta[i,3] <- mu[s[i],3] + d[t[i],3] - d[b[i],3]

}

# Adding in hierarchical model where class of intervention is above intervention in the hierarchy

d[2,1] ~ dnorm(D.d[1,1], prec.d) # docetaxel.100

d[3,1] ~ dnorm(D.d[1,1], prec.d) # docetaxel.60

d[4,1] ~ dnorm(D.d[2,1], prec.d) # docetaxel.60_bevacizumab

d[5,1] ~ dnorm(D.d[3,1], prec.d) # docetaxel.60_ramucirumab

d[6,1] ~ dnorm(D.d[4,1], prec.d) # docetaxel.75_erlotinib.150

d[7,1] ~ dnorm(D.d[5,1], prec.d) # docetaxel.75_nintedanib.non

d[8,1] ~ dnorm(D.d[5,1], prec.d) # docetaxel.75_nintedanib.sq

d[9,1] ~ dnorm(D.d[3,1], prec.d) # docetaxel.75_ramucirumab

d[10,1] ~ dnorm(D.d[6,1], prec.d) # erlotinib.150_0.00.pos

d[11,1] ~ dnorm(D.d[6,1], prec.d) # erlotinib.150_0.03.pos

d[12,1] ~ dnorm(D.d[6,1], prec.d) # erlotinib.150_0.05.pos

d[13,1] ~ dnorm(D.d[6,1], prec.d) # erlotinib.150_0.16.pos

d[14,1] ~ dnorm(D.d[6,1], prec.d) # erlotinib.150_0.56.pos

d[15,1] ~ dnorm(D.d[6,1], prec.d) # erlotinib.150_1.00.pos

d[16,1] ~ dnorm(D.d[7,1], prec.d) # erlotinib.150_pemetrexed.500.egfr.0.00

d[17,1] ~ dnorm(D.d[7,1], prec.d) # erlotinib.150_pemetrexed.500.egfr.0.56

d[18,1] ~ dnorm(D.d[8,1], prec.d) # gefitinib.250_0.00.pos

d[19,1] ~ dnorm(D.d[8,1], prec.d) # gefitinib.250_0.15.pos

d[20,1] ~ dnorm(D.d[8,1], prec.d) # gefitinib.250_0.52.pos

d[21,1] ~ dnorm(D.d[8,1], prec.d) # gefitinib.250_1.00.pos

d[22,1] ~ dnorm(D.d[9,1], prec.d) # nivolumab_non.sq_high.PDL1

d[23,1] ~ dnorm(D.d[9,1], prec.d) # nivolumab_non.sq_low.PDL1

d[24,1] ~ dnorm(D.d[9,1], prec.d) # nivolumab_sq_high.PDL1

d[25,1] ~ dnorm(D.d[9,1], prec.d) # nivolumab_sq_low.PDL1

d[26,1] ~ dnorm(D.d[10,1], prec.d) # pemetrexed.500_0.00.sq

d[27,1] ~ dnorm(D.d[10,1], prec.d) # pemetrexed.500_0.26.sq

d[28,1] ~ dnorm(D.d[10,1], prec.d) # pemetrexed.500_0.28.sq

d[29,1] ~ dnorm(D.d[11,1], prec.d) # pemetrexed.500_nintedanib

d[30,1] ~ dnorm(D.d[12,1], prec.d) # S1_bevacizumab

# Adding in hierarchical model where class of intervention is above intervention in the hierarchy

d[2,2] ~ dnorm(D.d[1,2], prec.d2) # docetaxel.100

d[3,2] ~ dnorm(D.d[1,2], prec.d2) # docetaxel.60

d[4,2] ~ dnorm(D.d[2,2], prec.d2) # docetaxel.60_bevacizumab

d[5,2] ~ dnorm(D.d[3,2], prec.d2) # docetaxel.60_ramucirumab

d[6,2] ~ dnorm(D.d[4,2], prec.d2) # docetaxel.75_erlotinib.150

d[7,2] ~ dnorm(D.d[5,2], prec.d2) # docetaxel.75_nintedanib.non

d[8,2] ~ dnorm(D.d[5,2], prec.d2) # docetaxel.75_nintedanib.sq

d[9,2] ~ dnorm(D.d[3,2], prec.d2) # docetaxel.75_ramucirumab

d[10,2] ~ dnorm(D.d[6,2], prec.d2) # erlotinib.150_0.00.pos

d[11,2] ~ dnorm(D.d[6,2], prec.d2) # erlotinib.150_0.03.pos

d[12,2] ~ dnorm(D.d[6,2], prec.d2) # erlotinib.150_0.05.pos

d[13,2] ~ dnorm(D.d[6,2], prec.d2) # erlotinib.150_0.16.pos

d[14,2] ~ dnorm(D.d[6,2], prec.d2) # erlotinib.150_0.56.pos

d[15,2] ~ dnorm(D.d[6,2], prec.d2) # erlotinib.150_1.00.pos

d[16,2] ~ dnorm(D.d[7,2], prec.d2) # erlotinib.150_pemetrexed.500.egfr.0.00

d[17,2] ~ dnorm(D.d[7,2], prec.d2) # erlotinib.150_pemetrexed.500.egfr.0.56

d[18,2] ~ dnorm(D.d[8,2], prec.d2) # gefitinib.250_0.00.pos

d[19,2] ~ dnorm(D.d[8,2], prec.d2) # gefitinib.250_0.15.pos

d[20,2] ~ dnorm(D.d[8,2], prec.d2) # gefitinib.250_0.47.pos

d[21,2] ~ dnorm(D.d[8,2], prec.d2) # gefitinib.250_1.00.pos

d[22,2] ~ dnorm(D.d[9,2], prec.d2) # nivolumab_non.sq_high.PDL1

d[23,2] ~ dnorm(D.d[9,2], prec.d2) # nivolumab_non.sq_low.PDL1

d[24,2] ~ dnorm(D.d[9,2], prec.d2) # nivolumab_sq_high.PDL1

d[25,2] ~ dnorm(D.d[9,2], prec.d2) # nivolumab_sq_low.PDL1

d[26,2] ~ dnorm(D.d[10,2], prec.d2) # pemetrexed.500_0.00.sq

d[27,2] ~ dnorm(D.d[10,2], prec.d2) # pemetrexed.500_0.26.sq

d[28,2] ~ dnorm(D.d[10,2], prec.d2) # pemetrexed.500_0.28.sq

d[29,2] ~ dnorm(D.d[11,2], prec.d2) # pemetrexed.500_nintedanib

d[30,2] ~ dnorm(D.d[12,2], prec.d2) # S1_bevacizumab

# Adding in hierarchical model where class of intervention is above intervention in the hierarchy

d[2,3] ~ dnorm(D.d[1,3], prec.d3) # docetaxel.100

d[3,3] ~ dnorm(D.d[1,3], prec.d3) # docetaxel.60

d[4,3] ~ dnorm(D.d[2,3], prec.d3) # docetaxel.60_bevacizumab

d[5,3] ~ dnorm(D.d[3,3], prec.d3) # docetaxel.60_ramucirumab

d[6,3] ~ dnorm(D.d[4,3], prec.d3) # docetaxel.75_erlotinib.150

d[7,3] ~ dnorm(D.d[5,3], prec.d3) # docetaxel.75_nintedanib.non

d[8,3] ~ dnorm(D.d[5,3], prec.d3) # docetaxel.75_nintedanib.sq

d[9,3] ~ dnorm(D.d[3,3], prec.d3) # docetaxel.75_ramucirumab

d[10,3] ~ dnorm(D.d[6,3], prec.d3) # erlotinib.150_0.00.pos

d[11,3] ~ dnorm(D.d[6,3], prec.d3) # erlotinib.150_0.03.pos

d[12,3] ~ dnorm(D.d[6,3], prec.d3) # erlotinib.150_0.05.pos

d[13,3] ~ dnorm(D.d[6,3], prec.d3) # erlotinib.150_0.16.pos

d[14,3] ~ dnorm(D.d[6,3], prec.d3) # erlotinib.150_0.56.pos

d[15,3] ~ dnorm(D.d[6,3], prec.d3) # erlotinib.150_1.00.pos

d[16,3] ~ dnorm(D.d[7,3], prec.d3) # erlotinib.150_pemetrexed.500.egfr.0.00

d[17,3] ~ dnorm(D.d[7,3], prec.d3) # erlotinib.150_pemetrexed.500.egfr.0.56

d[18,3] ~ dnorm(D.d[8,3], prec.d3) # gefitinib.250_0.00.pos

d[19,3] ~ dnorm(D.d[8,3], prec.d3) # gefitinib.250_0.15.pos

d[20,3] ~ dnorm(D.d[8,3], prec.d3) # gefitinib.250_0.52.pos

d[21,3] ~ dnorm(D.d[8,3], prec.d3) # gefitinib.250_1.00.pos

d[22,3] ~ dnorm(D.d[9,3], prec.d3) # nivolumab_non.sq_high.PDL1

d[23,3] ~ dnorm(D.d[9,3], prec.d3) # nivolumab_non.sq_low.PDL1

d[24,3] ~ dnorm(D.d[9,3], prec.d3) # nivolumab_sq_high.PDL1

d[25,3] ~ dnorm(D.d[9,3], prec.d3) # nivolumab_sq_low.PDL1

d[26,3] ~ dnorm(D.d[10,3], prec.d3) # pemetrexed.500_0.00.sq

d[27,3] ~ dnorm(D.d[10,3], prec.d3) # pemetrexed.500_0.26.sq

d[28,3] ~ dnorm(D.d[10,3], prec.d3) # pemetrexed.500_0.28.sq

d[29,3] ~ dnorm(D.d[11,3], prec.d3) # pemetrexed.500_nintedanib

d[30,3] ~ dnorm(D.d[12,3], prec.d3) # S1_bevacizumab

# Placing ordering constraints on scale

# constraints for erlotinib and EGFR +ve

gamma1 <- step(d[10,1] - d[11,1])

gamma2 <- step(d[11,1] - d[12,1])

gamma3 <- step(d[12,1] - d[13,1])

gamma4 <- step(d[13,1] - d[14,1])

gamma5 <- step(d[14,1] - d[15,1])

# constraints for erlotinib + pemetrexed and EGFR +ve

gamma6 <- step(d[16,1] - d[17,1])

# constraints for gefitinib and EGFR +ve

gamma7 <- step(d[18,1] - d[19,1])

gamma8 <- step(d[19,1] - d[20,1])

gamma9 <- step(d[20,1] - d[21,1])

# constraints for pemetrexed and squamous

gamma10 <- step(d[28,1] - d[27,1])

gamma11 <- step(d[27,1] - d[26,1])

# constraints for docetaxel

gamma12 <- step(d[3,1] - d[1,1])

# define binomial prior distributions

b1 ~ dbern(gamma1)

b2 ~ dbern(gamma2)

b3 ~ dbern(gamma3)

b4 ~ dbern(gamma4)

b5 ~ dbern(gamma5)

b6 ~ dbern(gamma6)

b7 ~ dbern(gamma7)

b8 ~ dbern(gamma8)

b9 ~ dbern(gamma9)

b10 ~ dbern(gamma10)

b11 ~ dbern(gamma11)

b12 ~ dbern(gamma12)

prec.d <- 1/(sd.d*sd.d)

sd.d ~ dunif(0,5)

prec.d2 <- 1/(sd.d2*sd.d2)

sd.d2 ~ dunif(0,5)

prec.d3 <- 1/(sd.d3*sd.d3)

sd.d3 ~ dunif(0,5)

for (i in 1:12){

D.d[i,1:3] ~ dmnorm(mean[1:3],prec2) # vague priors on class effects

}

d[1,1] <- 0

d[1,2] <- 0

d[1,3] <- 0

# priors

for(k in 1:NS){

mu[k, 1:3] ~ dmnorm(mean[1:3],prec2[,]) # priors

}

}

# In R

prec2 <- structure(.Data=c(0.0001, 0, 0, 0, 0.0001, 0, 0, 0, 0.0001),.Dim = c(3, 3))

## JAGS Code Used for Progression-Free Survival: Second-Order Random Scale-Effects Fractional Polynomial Survival Model With Hierarchical Exchangeable Structures

Adapted from Jansen [12] for fractional polynomial survival NMA and the hierarchical exchangeable model presented by Owen et al. [31].

cat( “

# set indicator variables to be 1

# datablock required for JAGS due to re-using objects with the same name - different from WinBUGS

data{

b1 <- 1

b2 <- 1

b3 <- 1

b4 <- 1

b5 <- 1

b6 <- 1

b7 <- 1

b8 <- 1

b9 <- 1

b10 <- 1

b11 <- 1

b12 <- 1

}

model {

for (i in 1:N) { # N number of datapoints in dataset

# time is expressed in months and transformed according powers of fractional polynomial P1 and P2

time_transf1[i] <- (equals(P1,0)*log(time[i]) + (1-equals(P1,0))*pow(time[i],P1))

time_transf2[i] <- ((1-equals(P2,P1))*(equals(P2,0)*log(time[i]) + (1-equals(P2,0))*pow(time[i],P2)) +

equals(P2,P1)*(equals(P2,0)*log(time[i])*log(time[i]) + (1-equals(P2,0))*pow(time[i],P2) *log(time[i])))

# likelihood

# hazard over interval [t,t+dt] expressed as deaths per person-month

# r is deaths in interval, n is number at risk, h is hazard

r[i]~ dbin(p[i],n[i])

p[i] <- 1-exp(-h[i]*dt[i]) # dt=time interval cumulative hazard over interval[t,t+dt] expressed as deaths per person-month

# random effects model

# loop over datapoints

# s refers to study, t is intervention t, b is comparator

log(h[i]) <- Beta[i,1] + Beta[i,2]*time_transf1[i] + Beta[i,3]*time_transf2[i]

Beta[i,1] <- mu[s[i],1] + delta[s[i]] * (1-equals(t[i],b[i]))

Beta[i,2] <- mu[s[i],2] + d[t[i],2] - d[b[i],2] # fixed for d1

Beta[i,3] <- mu[s[i],3] + d[t[i],3] - d[b[i],3] # fixed for d2

}

# loop over studies - used in random effects model

# NS is number of studies

# ts is intervention t, bs is comparator

for(m in 1:NS){

delta[m] ~ dnorm(md[m], tau)

md[m] <- d[ts[m],1]-d[bs[m],1] # random d0

}

sd ~ dunif(0,5)

tau <- 1/(sd*sd)

# Adding in hierarchical model where class of intervention is above intervention in the hierarchy

d[2,1] ~ dnorm(D.d[1,1], prec.d) # docetaxel.100

d[3,1] ~ dnorm(D.d[1,1], prec.d) # docetaxel.60

d[4,1] ~ dnorm(D.d[2,1], prec.d) # docetaxel.60_bevacizumab

d[5,1] ~ dnorm(D.d[3,1], prec.d) # docetaxel.60_ramucirumab

d[6,1] ~ dnorm(D.d[4,1], prec.d) # docetaxel.75_erlotinib.150

d[7,1] ~ dnorm(D.d[5,1], prec.d) # docetaxel.75_nintedanib.non

d[8,1] ~ dnorm(D.d[5,1], prec.d) # docetaxel.75_nintedanib.sq

d[9,1] ~ dnorm(D.d[3,1], prec.d) # docetaxel.75_ramucirumab

d[10,1] ~ dnorm(D.d[6,1], prec.d) # erlotinib.150_0.00.pos

d[11,1] ~ dnorm(D.d[6,1], prec.d) # erlotinib.150_0.03.pos

d[12,1] ~ dnorm(D.d[6,1], prec.d) # erlotinib.150_0.05.pos

d[13,1] ~ dnorm(D.d[6,1], prec.d) # erlotinib.150_0.16.pos

d[14,1] ~ dnorm(D.d[6,1], prec.d) # erlotinib.150_0.56.pos

d[15,1] ~ dnorm(D.d[6,1], prec.d) # erlotinib.150_1.00.pos

d[16,1] ~ dnorm(D.d[7,1], prec.d) # erlotinib.150_pemetrexed.500.egfr.0.00

d[17,1] ~ dnorm(D.d[7,1], prec.d) # erlotinib.150_pemetrexed.500.egfr.0.56

d[18,1] ~ dnorm(D.d[8,1], prec.d) # gefitinib.250_0.00.pos

d[19,1] ~ dnorm(D.d[8,1], prec.d) # gefitinib.250_0.15.pos

d[20,1] ~ dnorm(D.d[8,1], prec.d) # gefitinib.250_0.52.pos

d[21,1] ~ dnorm(D.d[8,1], prec.d) # gefitinib.250_1.00.pos

d[22,1] ~ dnorm(D.d[9,1], prec.d) # nivolumab_non.sq_high.PDL1

d[23,1] ~ dnorm(D.d[9,1], prec.d) # nivolumab_non.sq_low.PDL1

d[24,1] ~ dnorm(D.d[9,1], prec.d) # nivolumab_sq_high.PDL1

d[25,1] ~ dnorm(D.d[9,1], prec.d) # nivolumab_sq_low.PDL1

d[26,1] ~ dnorm(D.d[10,1], prec.d) # pemetrexed.500_0.00.sq

d[27,1] ~ dnorm(D.d[10,1], prec.d) # pemetrexed.500_0.26.sq

d[28,1] ~ dnorm(D.d[10,1], prec.d) # pemetrexed.500_0.28.sq

d[29,1] ~ dnorm(D.d[11,1], prec.d) # pemetrexed.500_nintedanib

d[30,1] ~ dnorm(D.d[12,1], prec.d) # S1_bevacizumab

# Adding in hierarchical model where class of intervention is above intervention in the hierarchy

d[2,2] ~ dnorm(D.d[1,2], prec.d2) # docetaxel.100

d[3,2] ~ dnorm(D.d[1,2], prec.d2) # docetaxel.60

d[4,2] ~ dnorm(D.d[2,2], prec.d2) # docetaxel.60_bevacizumab

d[5,2] ~ dnorm(D.d[3,2], prec.d2) # docetaxel.60_ramucirumab

d[6,2] ~ dnorm(D.d[4,2], prec.d2) # docetaxel.75_erlotinib.150

d[7,2] ~ dnorm(D.d[5,2], prec.d2) # docetaxel.75_nintedanib.non

d[8,2] ~ dnorm(D.d[5,2], prec.d2) # docetaxel.75_nintedanib.sq

d[9,2] ~ dnorm(D.d[3,2], prec.d2) # docetaxel.75_ramucirumab

d[10,2] ~ dnorm(D.d[6,2], prec.d2) # erlotinib.150_0.00.pos

d[11,2] ~ dnorm(D.d[6,2], prec.d2) # erlotinib.150_0.03.pos

d[12,2] ~ dnorm(D.d[6,2], prec.d2) # erlotinib.150_0.05.pos

d[13,2] ~ dnorm(D.d[6,2], prec.d2) # erlotinib.150_0.16.pos

d[14,2] ~ dnorm(D.d[6,2], prec.d2) # erlotinib.150_0.56.pos

d[15,2] ~ dnorm(D.d[6,2], prec.d2) # erlotinib.150_1.00.pos

d[16,2] ~ dnorm(D.d[7,2], prec.d2) # erlotinib.150_pemetrexed.500.egfr.0.00

d[17,2] ~ dnorm(D.d[7,2], prec.d2) # erlotinib.150_pemetrexed.500.egfr.0.56

d[18,2] ~ dnorm(D.d[8,2], prec.d2) # gefitinib.250_0.00.pos

d[19,2] ~ dnorm(D.d[8,2], prec.d2) # gefitinib.250_0.15.pos

d[20,2] ~ dnorm(D.d[8,2], prec.d2) # gefitinib.250_0.52.pos

d[21,2] ~ dnorm(D.d[8,2], prec.d2) # gefitinib.250_1.00.pos

d[22,2] ~ dnorm(D.d[9,2], prec.d2) # nivolumab_non.sq_high.PDL1

d[23,2] ~ dnorm(D.d[9,2], prec.d2) # nivolumab_non.sq_low.PDL1

d[24,2] ~ dnorm(D.d[9,2], prec.d2) # nivolumab_sq_high.PDL1

d[25,2] ~ dnorm(D.d[9,2], prec.d2) # nivolumab_sq_low.PDL1

d[26,2] ~ dnorm(D.d[10,2], prec.d2) # pemetrexed.500_0.00.sq

d[27,2] ~ dnorm(D.d[10,2], prec.d2) # pemetrexed.500_0.26.sq

d[28,2] ~ dnorm(D.d[10,2], prec.d2) # pemetrexed.500_0.28.sq

d[29,2] ~ dnorm(D.d[11,2], prec.d2) # pemetrexed.500_nintedanib

d[30,2] ~ dnorm(D.d[12,2], prec.d2) # S1_bevacizumab

# Adding in hierarchical model where class of intervention is above intervention in the hierarchy

d[2,3] ~ dnorm(D.d[1,3], prec.d3) # docetaxel.100

d[3,3] ~ dnorm(D.d[1,3], prec.d3) # docetaxel.60

d[4,3] ~ dnorm(D.d[2,3], prec.d3) # docetaxel.60_bevacizumab

d[5,3] ~ dnorm(D.d[3,3], prec.d3) # docetaxel.60_ramucirumab

d[6,3] ~ dnorm(D.d[4,3], prec.d3) # docetaxel.75_erlotinib.150

d[7,3] ~ dnorm(D.d[5,3], prec.d3) # docetaxel.75_nintedanib.non

d[8,3] ~ dnorm(D.d[5,3], prec.d3) # docetaxel.75_nintedanib.sq

d[9,3] ~ dnorm(D.d[3,3], prec.d3) # docetaxel.75_ramucirumab

d[10,3] ~ dnorm(D.d[6,3], prec.d3) # erlotinib.150_0.00.pos

d[11,3] ~ dnorm(D.d[6,3], prec.d3) # erlotinib.150_0.03.pos

d[12,3] ~ dnorm(D.d[6,3], prec.d3) # erlotinib.150_0.05.pos

d[13,3] ~ dnorm(D.d[6,3], prec.d3) # erlotinib.150_0.16.pos

d[14,3] ~ dnorm(D.d[6,3], prec.d3) # erlotinib.150_0.56.pos

d[15,3] ~ dnorm(D.d[6,3], prec.d3) # erlotinib.150_1.00.pos

d[16,3] ~ dnorm(D.d[7,3], prec.d3) # erlotinib.150_pemetrexed.500.egfr.0.00

d[17,3] ~ dnorm(D.d[7,3], prec.d3) # erlotinib.150_pemetrexed.500.egfr.0.56

d[18,3] ~ dnorm(D.d[8,3], prec.d3) # gefitinib.250_0.00.pos

d[19,3] ~ dnorm(D.d[8,3], prec.d3) # gefitinib.250_0.15.pos

d[20,3] ~ dnorm(D.d[8,3], prec.d3) # gefitinib.250_0.52.pos

d[21,3] ~ dnorm(D.d[8,3], prec.d3) # gefitinib.250_1.00.pos

d[22,3] ~ dnorm(D.d[9,3], prec.d3) # nivolumab_non.sq_high.PDL1

d[23,3] ~ dnorm(D.d[9,3], prec.d3) # nivolumab_non.sq_low.PDL1

d[24,3] ~ dnorm(D.d[9,3], prec.d3) # nivolumab_sq_high.PDL1

d[25,3] ~ dnorm(D.d[9,3], prec.d3) # nivolumab_sq_low.PDL1

d[26,3] ~ dnorm(D.d[10,3], prec.d3) # pemetrexed.500_0.00.sq

d[27,3] ~ dnorm(D.d[10,3], prec.d3) # pemetrexed.500_0.26.sq

d[28,3] ~ dnorm(D.d[10,3], prec.d3) # pemetrexed.500_0.28.sq

d[29,3] ~ dnorm(D.d[11,3], prec.d3) # pemetrexed.500_nintedanib

d[30,3] ~ dnorm(D.d[12,3], prec.d3) # S1_bevacizumab

# Placing ordering constraints on increasing EGFR pos for erlotinib

# constraints for erlotinib and EGFR +ve

gamma1 <- step(d[10,1] - d[11,1])

gamma2 <- step(d[11,1] - d[12,1])

gamma3 <- step(d[12,1] - d[13,1])

gamma4 <- step(d[13,1] - d[14,1])

gamma5 <- step(d[14,1] - d[15,1])

# constraints for erlotinib + pemetrexedand EGFR +ve

gamma6 <- step(d[16,1] - d[17,1])

# constraints for gefitinib and EGFR +ve

gamma7 <- step(d[18,1] - d[19,1])

gamma8 <- step(d[19,1] - d[20,1])

gamma9 <- step(d[20,1] - d[21,1])

# constraints for pemetrexed and squamous

gamma10 <- step(d[28,1] - d[27,1])

gamma11 <- step(d[27,1] - d[26,1])

# constraints for docetaxel

gamma12 <- step(d[3,1] - d[1,1])

# define binomial prior distributions

b1 ~ dbern(gamma1)

b2 ~ dbern(gamma2)

b3 ~ dbern(gamma3)

b4 ~ dbern(gamma4)

b5 ~ dbern(gamma5)

b6 ~ dbern(gamma6)

b7 ~ dbern(gamma7)

b8 ~ dbern(gamma8)

b9 ~ dbern(gamma9)

b10 ~ dbern(gamma10)

b11 ~ dbern(gamma11)

b12 ~ dbern(gamma12)

prec.d <- 1/(sd.d*sd.d)

sd.d ~ dunif(0,5)

prec.d2 <- 1/(sd.d2*sd.d2)

sd.d2 ~ dunif(0,5)

prec.d3 <- 1/(sd.d3*sd.d3)

sd.d3 ~ dunif(0,5)

for (i in 1:12){

D.d[i,1:3] ~ dmnorm(mean[1:3],prec2) # vague priors on class effects

}

# priors

# NT is number of treatments

d[1,1] <- 0

d[1,2] <- 0

d[1,3] <- 0

for(k in 1:NS){

mu[k,1:3] ~ dmnorm(mean[1:3],prec2[,])

}

}

# In R

prec2 <- structure(.Data=c(0.0001, 0, 0, 0, 0.0001, 0, 0, 0, 0.0001),.Dim = c(3, 3))

## JAGS Code Used for Progression-Free Survival: Second-Order Random Scale- and Shape- Effects Fractional Polynomial Survival Model With Hierarchical Exchangeable Structures

Adapted from Jansen [12] for fractional polynomial survival NMA and the hierarchical exchangeable model presented by Owen et al. [31].

cat( “

# set indicator variables to be 1

# datablock required for JAGS due to re-using objects with the same name - different from WinBUGS

data{

b1 <- 1

b2 <- 1

b3 <- 1

b4 <- 1

b5 <- 1

b6 <- 1

b7 <- 1

b8 <- 1

b9 <- 1

b10 <- 1

b11 <- 1

b12 <- 1

b13 <- 1

b14 <- 1

b15 <- 1

}

model {

for (i in 1:N) { # N number of datapoints in dataset

# time is expressed in months and transformed according powers of fractional polynomial P1 and P2

time_transf1[i] <- (equals(P1,0)*log(time[i]) + (1-equals(P1,0))*pow(time[i],P1))

time_transf2[i] <- ((1-equals(P2,P1))*(equals(P2,0)*log(time[i]) + (1-equals(P2,0))*pow(time[i],P2)) +

equals(P2,P1)*(equals(P2,0)*log(time[i])*log(time[i]) + (1-equals(P2,0))*pow(time[i],P2) *log(time[i])))

# likelihood

# hazard over interval [t,t+dt] expressed as deaths per person-month

# r is deaths in interval, n is number at risk, h is hazard

r[i]~ dbin(p[i],n[i])

p[i] <- 1-exp(-h[i]*dt[i]) # dt=time interval cumulative hazard over interval[t,t+dt] expressed as deaths per person-month

# random effects model

# loop over datapoints

# s refers to study, t is intervention t, b is comparator

log(h[i]) <- Beta[i,1] + Beta[i,2]*time_transf1[i] + Beta[i,3]*time_transf2[i]

Beta[i,1] <- mu[s[i],1] + delta[s[i],1] * (1-equals(t[i],b[i]))

Beta[i,2] <- mu[s[i],2] + delta[s[i],2] * (1-equals(t[i],b[i]))

Beta[i,3] <- mu[s[i],3] + delta[s[i],3] * (1-equals(t[i],b[i]))

}

# loop over studies - used in random effects model

# NS is number of studies

# ts is intervention t, bs is comparator

for(m in 1:NS){

delta[m,1:3] ~ dmnorm(md[m,1:3],omega[1:3,1:3])

md[m,1] <- d[ts[m],1]-d[bs[m],1]

md[m,2] <- d[ts[m],2]-d[bs[m],2]

md[m,3] <- d[ts[m],3]-d[bs[m],3]

}

# Adding in hierarchical model where class of intervention is above intervention in the hierarchy

d[2,1] ~ dnorm(D.d[1,1], prec.d) # best.supportive.care

d[3,1] ~ dnorm(D.d[2,1], prec.d) # docetaxel.100

d[4,1] ~ dnorm(D.d[2,1], prec.d) # docetaxel.60

d[5,1] ~ dnorm(D.d[3,1], prec.d) # docetaxel.60_bevacizumab

d[6,1] ~ dnorm(D.d[4,1], prec.d) # docetaxel.60_ramucirumab

d[7,1] ~ dnorm(D.d[5,1], prec.d) # docetaxel.75_erlotinib.150

d[8,1] ~ dnorm(D.d[6,1], prec.d) # docetaxel.75_nintedanib.non

d[9,1] ~ dnorm(D.d[6,1], prec.d) # docetaxel.75_nintedanib.sq

d[10,1] ~ dnorm(D.d[4,1], prec.d) # docetaxel.75_ramucirumab

d[11,1] ~ dnorm(D.d[2,1], prec.d) # docetaxel.fr

d[12,1] ~ dnorm(D.d[7,1], prec.d) # erlotinib.150_0.00.pos

d[13,1] ~ dnorm(D.d[7,1], prec.d) # erlotinib.150_0.03.pos

d[14,1] ~ dnorm(D.d[7,1], prec.d) # erlotinib.150_0.05.pos

d[15,1] ~ dnorm(D.d[7,1], prec.d) # erlotinib.150_0.13.pos

d[16,1] ~ dnorm(D.d[7,1], prec.d) # erlotinib.150_0.16.pos

d[17,1] ~ dnorm(D.d[7,1], prec.d) # erlotinib.150_0.56.pos

d[18,1] ~ dnorm(D.d[7,1], prec.d) # erlotinib.150_0.80.pos

d[19,1] ~ dnorm(D.d[8,1], prec.d) # erlotinib.150_pemetrexed.500.egfr.0.00

d[20,1] ~ dnorm(D.d[8,1], prec.d) # erlotinib.150_pemetrexed.500.egfr.0.56

d[21,1] ~ dnorm(D.d[9,1], prec.d) # gefitinib.250_0.00.pos

d[22,1] ~ dnorm(D.d[9,1], prec.d) # gefitinib.250_0.15.pos

d[23,1] ~ dnorm(D.d[9,1], prec.d) # gefitinib.250_0.52.pos

d[24,1] ~ dnorm(D.d[9,1], prec.d) # gefitinib.250_0.81.pos

d[25,1] ~ dnorm(D.d[10,1], prec.d) # nivolumab_non.sq_high.PDL1

d[26,1] ~ dnorm(D.d[10,1], prec.d) # nivolumab_non.sq_low.PDL1

d[27,1] ~ dnorm(D.d[10,1], prec.d) # nivolumab_sq_high.PDL1

d[28,1] ~ dnorm(D.d[10,1], prec.d) # nivolumab_sq_low.PDL1

d[29,1] ~ dnorm(D.d[11,1], prec.d) # pemetrexed.500_0.00.sq

d[30,1] ~ dnorm(D.d[11,1], prec.d) # pemetrexed.500_0.23.sq

d[31,1] ~ dnorm(D.d[11,1], prec.d) # pemetrexed.500_0.26.sq

d[32,1] ~ dnorm(D.d[11,1], prec.d) # pemetrexed.500_1.00.sq

d[33,1] ~ dnorm(D.d[12,1], prec.d) # pemetrexed.500_nintedanib

d[34,1] ~ dnorm(D.d[13,1], prec.d) # S1_bevacizumab

# Adding in hierarchical model where class of intervention is above intervention in the hierarchy

d[2,2] ~ dnorm(D.d[1,2], prec.d2) # best.supportive.care

d[3,2] ~ dnorm(D.d[2,2], prec.d2) # docetaxel.100

d[4,2] ~ dnorm(D.d[2,2], prec.d2) # docetaxel.60

d[5,2] ~ dnorm(D.d[3,2], prec.d2) # docetaxel.60_bevacizumab

d[6,2] ~ dnorm(D.d[4,2], prec.d2) # docetaxel.60_ramucirumab

d[7,2] ~ dnorm(D.d[5,2], prec.d2) # docetaxel.75_erlotinib.150

d[8,2] ~ dnorm(D.d[6,2], prec.d2) # docetaxel.75_nintedanib.non

d[9,2] ~ dnorm(D.d[6,2], prec.d2) # docetaxel.75_nintedanib.sq

d[10,2] ~ dnorm(D.d[4,2], prec.d2) # docetaxel.75_ramucirumab

d[11,2] ~ dnorm(D.d[2,2], prec.d2) # docetaxel.fr

d[12,2] ~ dnorm(D.d[7,2], prec.d2) # erlotinib.150_0.00.pos

d[13,2] ~ dnorm(D.d[7,2], prec.d2) # erlotinib.150_0.03.pos

d[14,2] ~ dnorm(D.d[7,2], prec.d2) # erlotinib.150_0.05.pos

d[15,2] ~ dnorm(D.d[7,2], prec.d2) # erlotinib.150_0.13.pos

d[16,2] ~ dnorm(D.d[7,2], prec.d2) # erlotinib.150_0.16.pos

d[17,2] ~ dnorm(D.d[7,2], prec.d2) # erlotinib.150_0.56.pos

d[18,2] ~ dnorm(D.d[7,2], prec.d2) # erlotinib.150_0.80.pos

d[19,2] ~ dnorm(D.d[8,2], prec.d2) # erlotinib.150_pemetrexed.500.egfr.0.00

d[20,2] ~ dnorm(D.d[8,2], prec.d2) # erlotinib.150_pemetrexed.500.egfr.0.56

d[21,2] ~ dnorm(D.d[9,2], prec.d2) # gefitinib.250_0.00.pos

d[22,2] ~ dnorm(D.d[9,2], prec.d2) # gefitinib.250_0.15.pos

d[23,2] ~ dnorm(D.d[9,2], prec.d2) # gefitinib.250_0.52.pos

d[24,2] ~ dnorm(D.d[9,2], prec.d2) # gefitinib.250_0.81.pos

d[25,2] ~ dnorm(D.d[10,2], prec.d2) # nivolumab_non.sq_high.PDL1

d[26,2] ~ dnorm(D.d[10,2], prec.d2) # nivolumab_non.sq_low.PDL1

d[27,2] ~ dnorm(D.d[10,2], prec.d2) # nivolumab_sq_high.PDL1

d[28,2] ~ dnorm(D.d[10,2], prec.d2) # nivolumab_sq_low.PDL1

d[29,2] ~ dnorm(D.d[11,2], prec.d2) # pemetrexed.500_0.00.sq

d[30,2] ~ dnorm(D.d[11,2], prec.d2) # pemetrexed.500_0.23.sq

d[31,2] ~ dnorm(D.d[11,2], prec.d2) # pemetrexed.500_0.26.sq

d[32,2] ~ dnorm(D.d[11,2], prec.d2) # pemetrexed.500_1.00.sq

d[33,2] ~ dnorm(D.d[12,2], prec.d2) # pemetrexed.500_nintedanib

d[34,2] ~ dnorm(D.d[13,2], prec.d2) # S1_bevacizumab

# Adding in hierarchical model where class of intervention is above intervention in the hierarchy

d[2,3] ~ dnorm(D.d[1,2], prec.d3) # best.supportive.care

d[3,3] ~ dnorm(D.d[2,2], prec.d3) # docetaxel.100

d[4,3] ~ dnorm(D.d[2,2], prec.d3) # docetaxel.60

d[5,3] ~ dnorm(D.d[3,2], prec.d3) # docetaxel.60_bevacizumab

d[6,3] ~ dnorm(D.d[4,2], prec.d3) # docetaxel.60_ramucirumab

d[7,3] ~ dnorm(D.d[5,2], prec.d3) # docetaxel.75_erlotinib.150

d[8,3] ~ dnorm(D.d[6,2], prec.d3) # docetaxel.75_nintedanib.non

d[9,3] ~ dnorm(D.d[6,2], prec.d3) # docetaxel.75_nintedanib.sq

d[10,3] ~ dnorm(D.d[4,2], prec.d3) # docetaxel.75_ramucirumab

d[11,3] ~ dnorm(D.d[2,2], prec.d3) # docetaxel.fr

d[12,3] ~ dnorm(D.d[7,2], prec.d3) # erlotinib.150_0.00.pos

d[13,3] ~ dnorm(D.d[7,2], prec.d3) # erlotinib.150_0.03.pos

d[14,3] ~ dnorm(D.d[7,2], prec.d3) # erlotinib.150_0.05.pos

d[15,3] ~ dnorm(D.d[7,2], prec.d3) # erlotinib.150_0.13.pos

d[16,3] ~ dnorm(D.d[7,2], prec.d3) # erlotinib.150_0.16.pos

d[17,3] ~ dnorm(D.d[7,2], prec.d3) # erlotinib.150_0.56.pos

d[18,3] ~ dnorm(D.d[7,2], prec.d3) # erlotinib.150_0.80.pos

d[19,3] ~ dnorm(D.d[8,2], prec.d3) # erlotinib.150_pemetrexed.500.egfr.0.00

d[20,3] ~ dnorm(D.d[8,2], prec.d3) # erlotinib.150_pemetrexed.500.egfr.0.56

d[21,3] ~ dnorm(D.d[9,2], prec.d3) # gefitinib.250_0.00.pos

d[22,3] ~ dnorm(D.d[9,2], prec.d3) # gefitinib.250_0.15.pos

d[23,3] ~ dnorm(D.d[9,2], prec.d3) # gefitinib.250_0.52.pos

d[24,3] ~ dnorm(D.d[9,2], prec.d3) # gefitinib.250_0.81.pos

d[25,3] ~ dnorm(D.d[10,2], prec.d3) # nivolumab_non.sq_high.PDL1

d[26,3] ~ dnorm(D.d[10,2], prec.d3) # nivolumab_non.sq_low.PDL1

d[27,3] ~ dnorm(D.d[10,2], prec.d3) # nivolumab_sq_high.PDL1

d[28,3] ~ dnorm(D.d[10,2], prec.d3) # nivolumab_sq_low.PDL1

d[29,3] ~ dnorm(D.d[11,2], prec.d3) # pemetrexed.500_0.00.sq

d[30,3] ~ dnorm(D.d[11,2], prec.d3) # pemetrexed.500_0.23.sq

d[31,3] ~ dnorm(D.d[11,2], prec.d3) # pemetrexed.500_0.26.sq

d[32,3] ~ dnorm(D.d[11,2], prec.d3) # pemetrexed.500_1.00.sq

d[33,3] ~ dnorm(D.d[12,2], prec.d3) # pemetrexed.500_nintedanib

d[34,3] ~ dnorm(D.d[13,2], prec.d3) # S1_bevacizumab

# Placing ordering constraints

# constraints for erlotinib and EGFR +ve

gamma1 <- step(d[12,1] - d[13,1])

gamma2 <- step(d[13,1] - d[14,1])

gamma3 <- step(d[14,1] - d[15,1])

gamma4 <- step(d[15,1] - d[16,1])

gamma5 <- step(d[16,1] - d[17,1])

gamma6 <- step(d[17,1] - d[18,1])

# constraints for erlotinib + pemetrexedand EGFR +ve

gamma7 <- step(d[19,1] - d[20,1])

# constraints for gefitinib and EGFR +ve

gamma8 <- step(d[21,1] - d[22,1])

gamma9 <- step(d[22,1] - d[23,1])

gamma10 <- step(d[23,1] - d[24,1])

# constraints for pemetrexed and squamous

gamma11 <- step(d[32,1] - d[31,1])

gamma12 <- step(d[31,1] - d[30,1])

gamma13 <- step(d[30,1] - d[29,1])

# constraints for docetaxel

gamma14 <- step(d[11,1] - d[4,1])

gamma15 <- step(d[4,1] - d[1,1])

# define binomial prior distributions

b1 ~ dbern(gamma1)

b2 ~ dbern(gamma2)

b3 ~ dbern(gamma3)

b4 ~ dbern(gamma4)

b5 ~ dbern(gamma5)

b6 ~ dbern(gamma6)

b7 ~ dbern(gamma7)

b8 ~ dbern(gamma8)

b9 ~ dbern(gamma9)

b10 ~ dbern(gamma10)

b11 ~ dbern(gamma11)

b12 ~ dbern(gamma12)

b13 ~ dbern(gamma13)

b14 ~ dbern(gamma14)

b15 ~ dbern(gamma15)

prec.d <- 1/(sd.d*sd.d)

sd.d ~ dunif(0,5)

prec.d2 <- 1/(sd.d2*sd.d2)

sd.d2 ~ dunif(0,5)

prec.d3 <- 1/(sd.d3*sd.d3)

sd.d3 ~ dunif(0,5)

for (i in 1:13){

D.d[i,1:3] ~ dmnorm(mean[1:3],prec2) # vague priors on class effects

}

# priors

# NT is number of treatments

d[1,1] <- 0

d[1,2] <- 0

d[1,3] <- 0

for(k in 1:NS){

mu[k,1:3] ~ dmnorm(mean[1:3],prec2[,])

}

omega[1:3, 1:3] ~ dwish(R[1:3,1:3],3)

# output SD based on estimated covariance matrix

sigma.theta[1:3,1:3] <- inverse(omega[1:3,1:3])

sd[1] <- sqrt(sigma.theta[1,1])

sd[2] <- sqrt(sigma.theta[2,2])

sd[3] <- sqrt(sigma.theta[3,3])

}

# In R

prec2 <- structure(.Data=c(0.0001, 0, 0, 0, 0.0001, 0, 0, 0, 0.0001),.Dim = c(3, 3))
